# Supplementary material for: Genotype-by-environment interactions shape ubiquitin-proteasome system activity
Source: bioRxiv. 2024 Nov 21:2024.11.21.624644. Preprint. [Version 1] doi: 10.1101/2024.11.21.624644 (PMC11601593; doi:10.1101/2024.11.21.624644)
Supplement: Supplement 3 [file media-3.pdf]

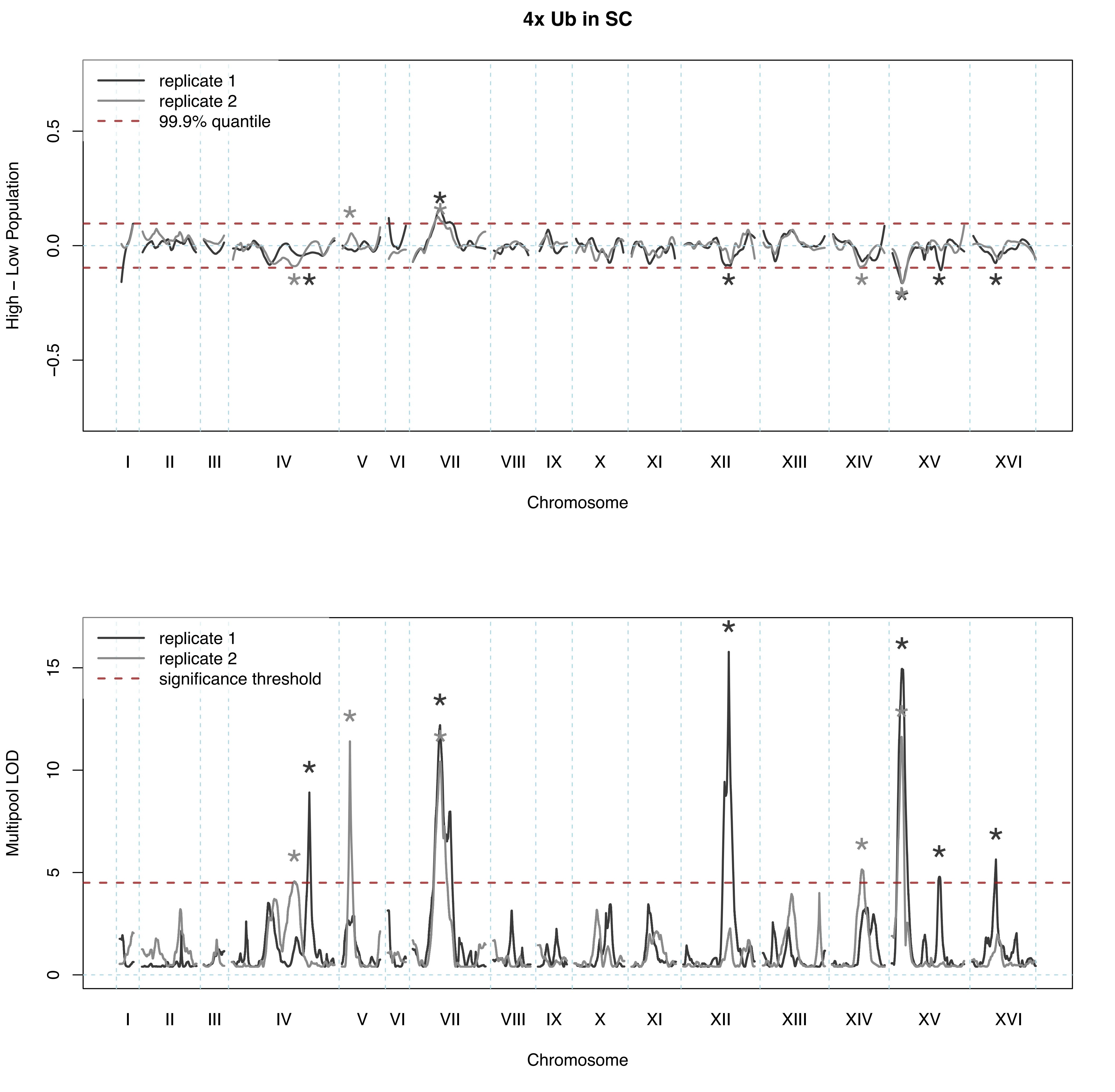
4x Ub in SC
replicate 1
replicate 2
99.9% quantile
High – Low Population
0.5
0.0
−0.5
I II III IV V VI VII VIII IX X XI XII XIII XIV XV XVI
Chromosome
replicate 1
replicate 2
significance threshold
Multipool LOD
15
10
5
0
I II III IV V VI VII VIII IX X XI XII XIII XIV XV XVI
Chromosome

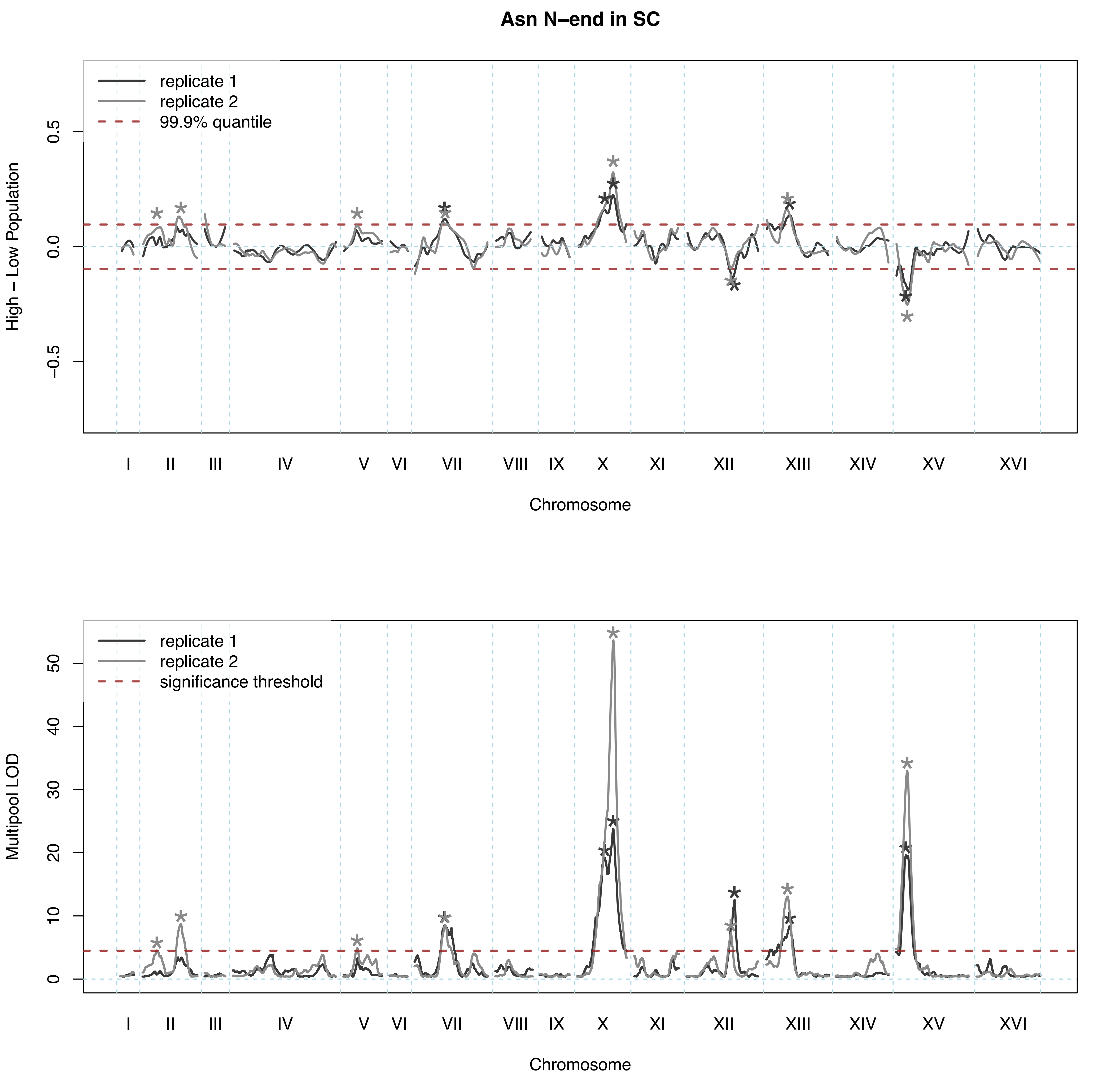
Asn N–end in SC
replicate 1
replicate 2
99.9% quantile
High – Low Population
0.5
0.0
−0.5
I II III IV V VI VII VIII IX X XI XII XIII XIV XV XVI
Chromosome
replicate 1
replicate 2
significance threshold
Multipool LOD
50
40
30
20
10
0
I II III IV V VI VII VIII IX X XI XII XIII XIV XV XVI
Chromosome

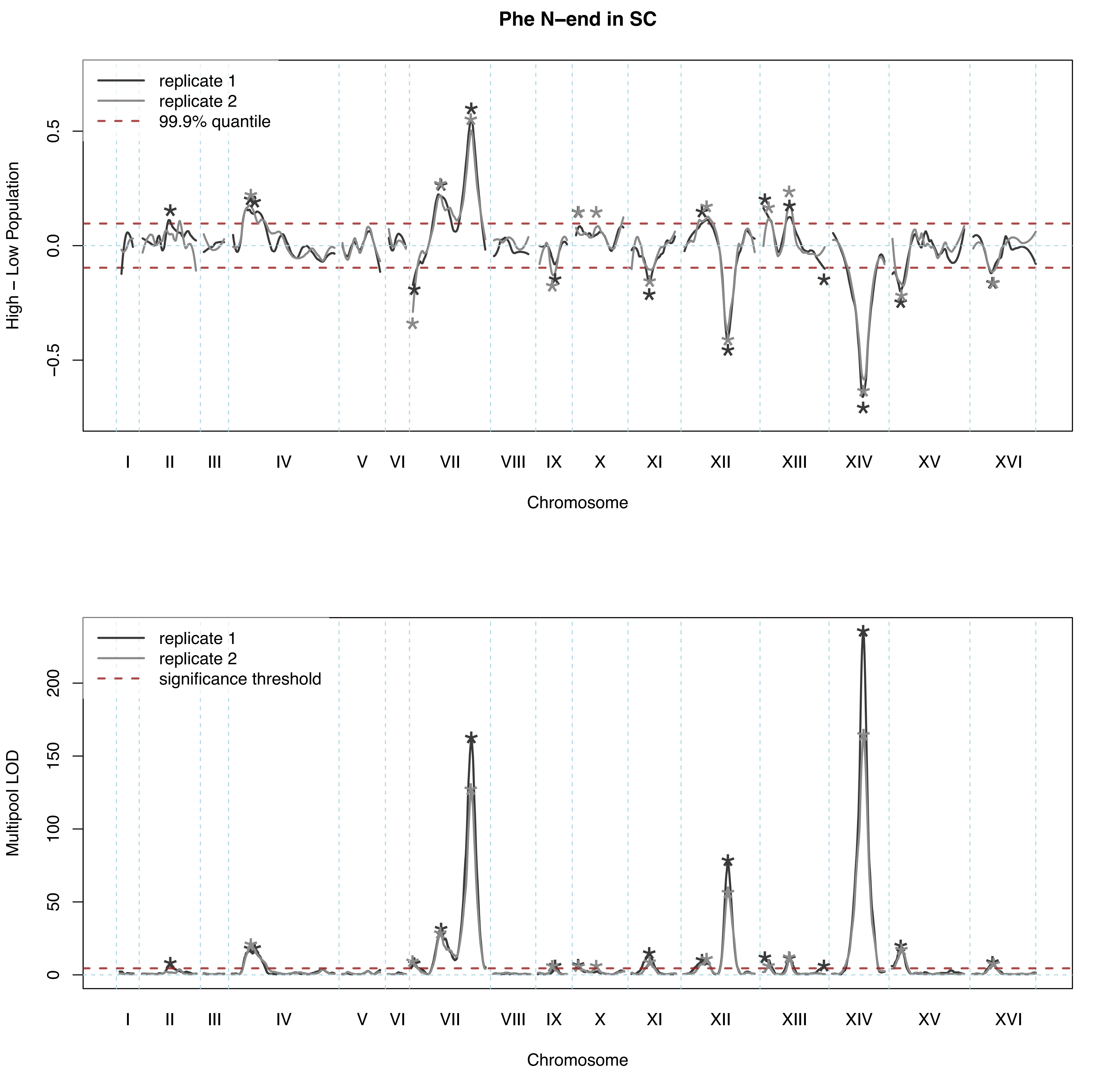
Phe N–end in SC
replicate 1
replicate 2
99.9% quantile
High – Low Population
0.5
0.0
−0.5
I
II
III
IV
V
VI
VII
VIII
IX
X
XI
XII
XIII
XIV
XV
XVI
Chromosome
replicate 1
replicate 2
significance threshold
Multipool LOD
200
150
100
50
0
I
II
III
IV
V
VI
VII
VIII
IX
X
XI
XII
XIII
XIV
XV
XVI
Chromosome

# rpn4 degron in SC

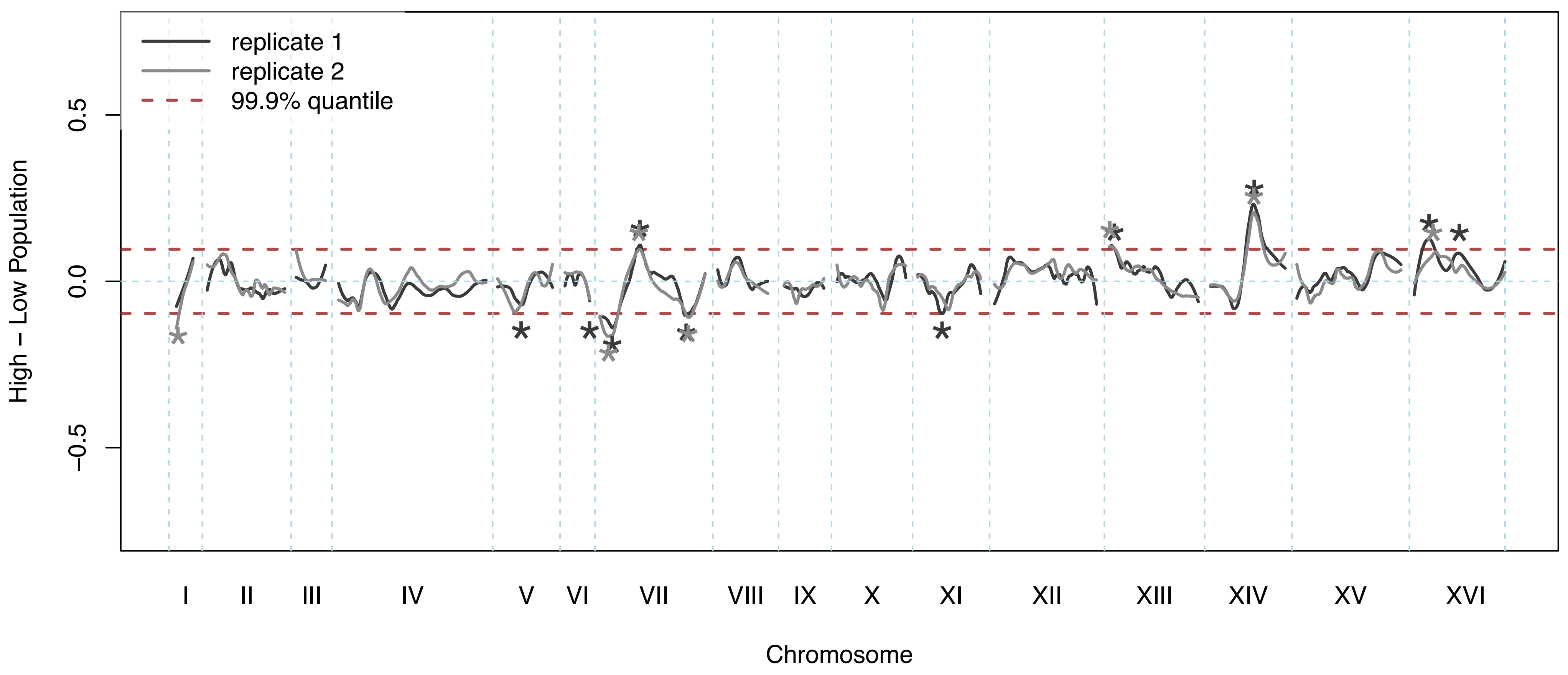


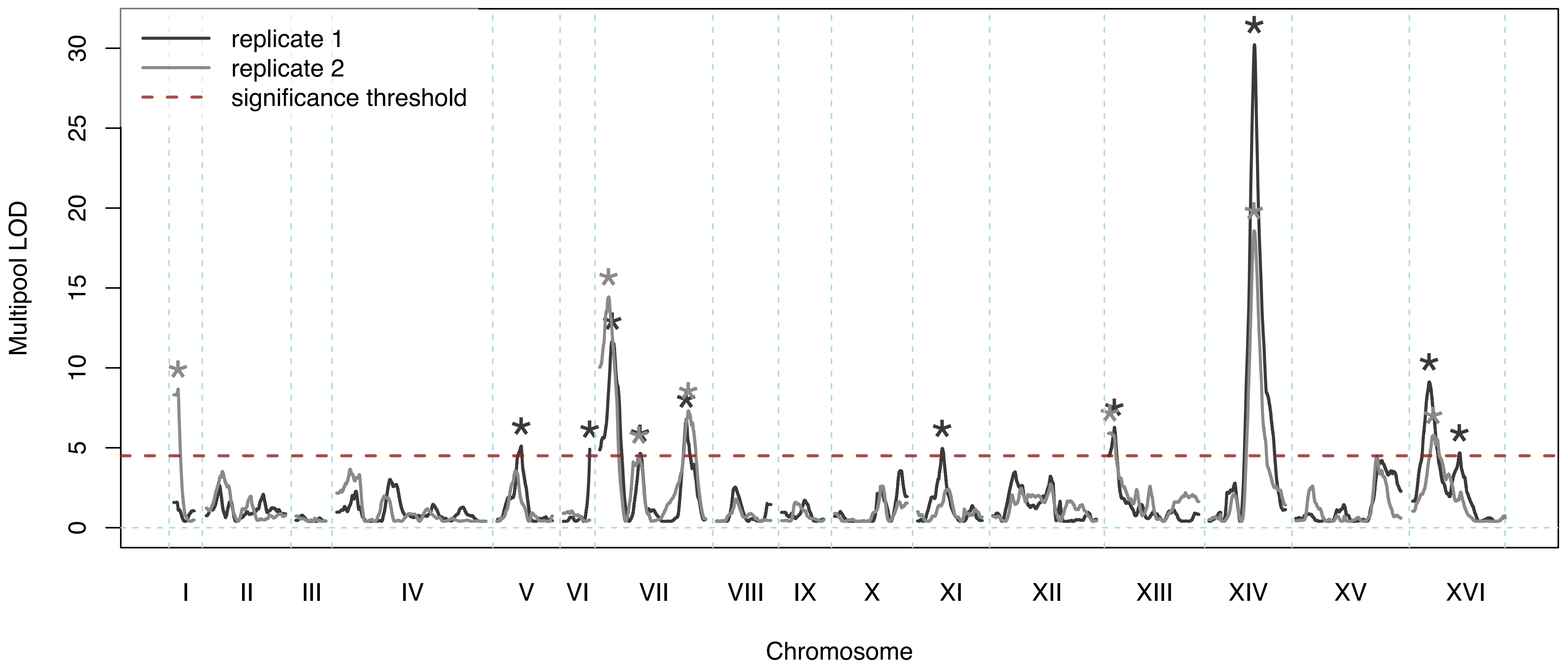

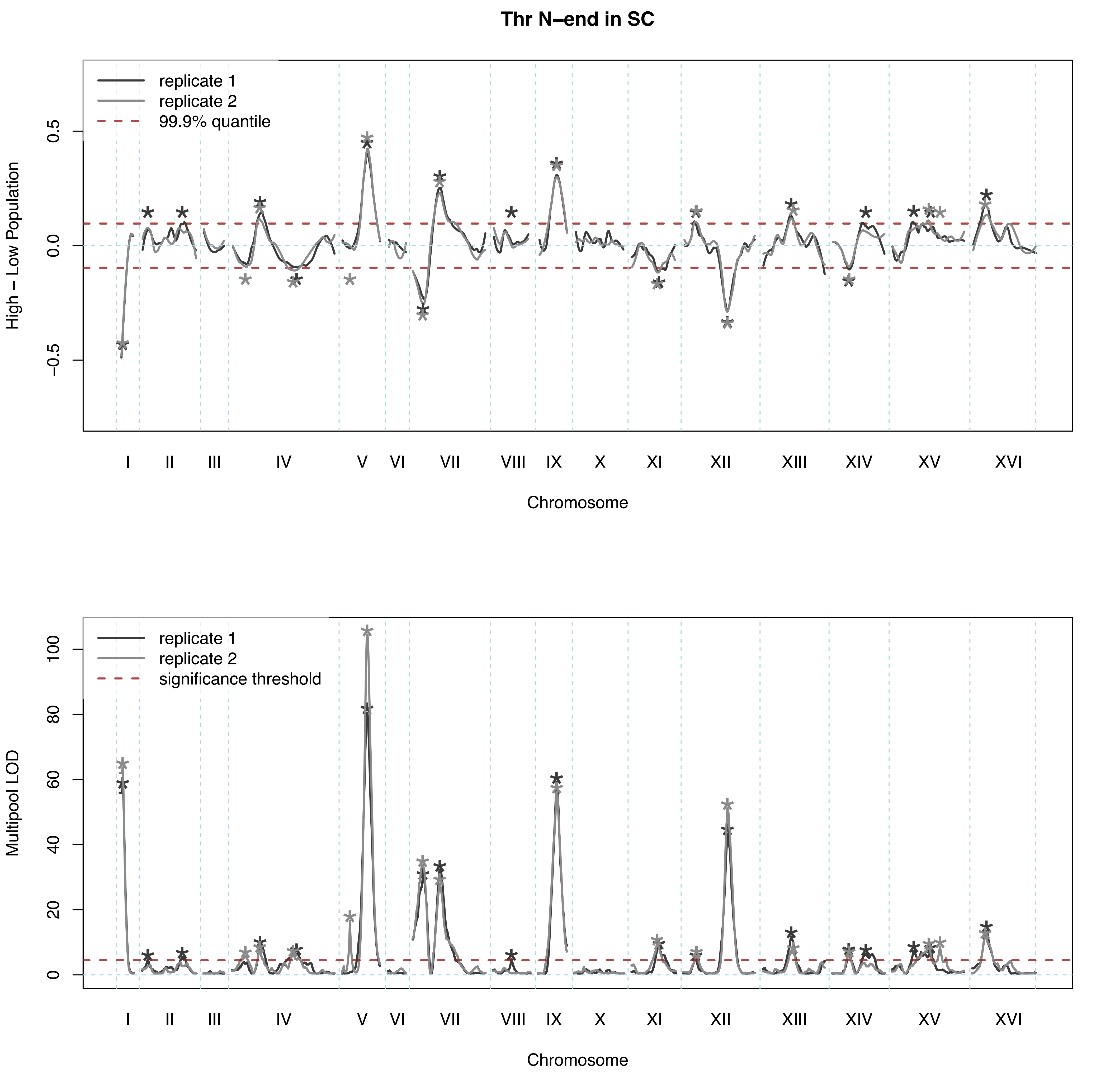

Thr N–end in SC
replicate 1
replicate 2
99.9% quantile
High – Low Population
0.5
0.0
−0.5
I
II
III
IV
V
VI
VII
VIII
IX
X
XI
XII
XIII
XIV
XV
XVI
Chromosome
replicate 1
replicate 2
significance threshold
Multipool LOD
100
80
60
40
20
0
I
II
III
IV
V
VI
VII
VIII
IX
X
XI
XII
XIII
XIV
XV
XVI
Chromosome

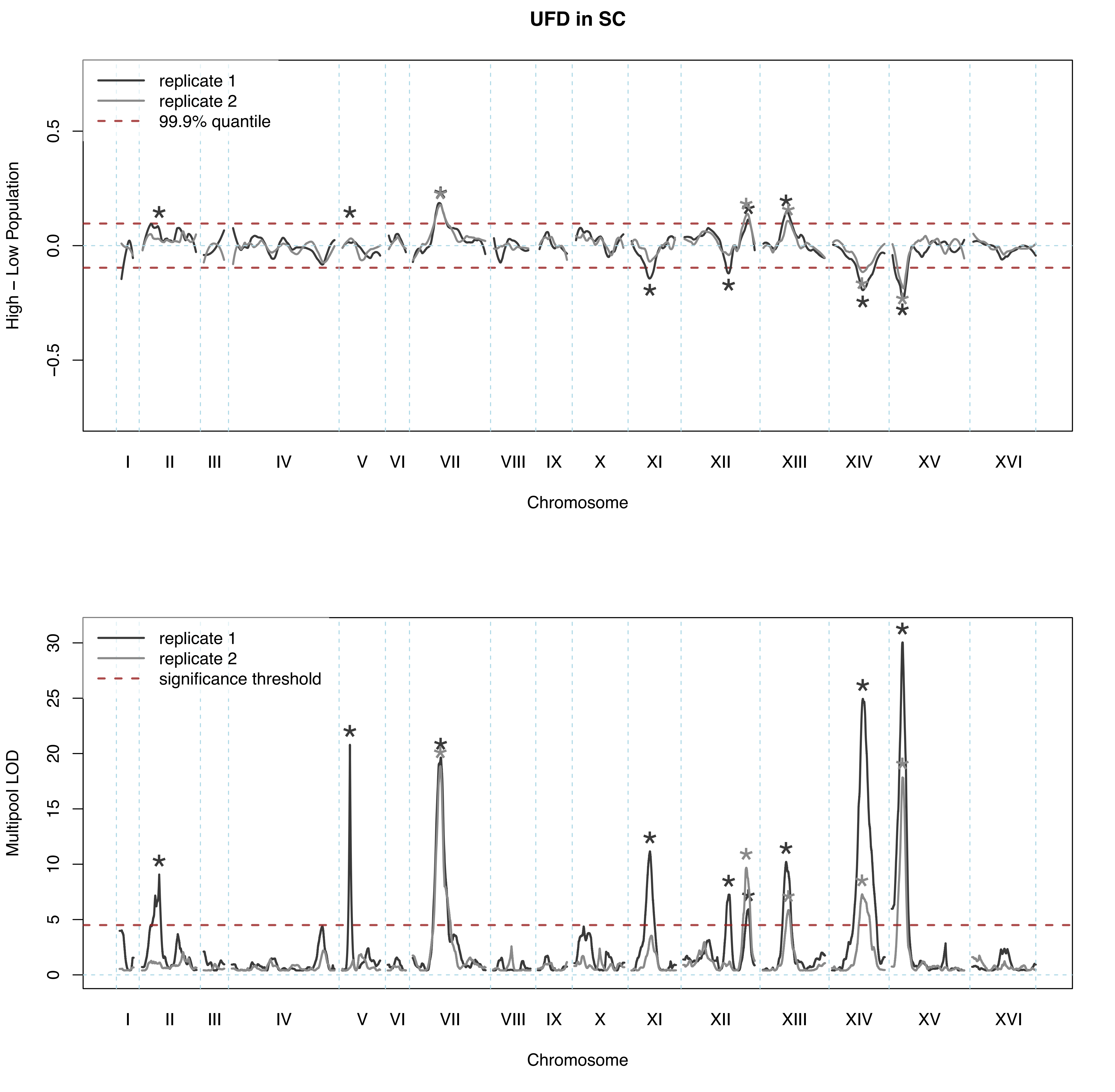

UFD in SC
replicate 1
replicate 2
99.9% quantile
High – Low Population
0.5
0.0
−0.5
I
II
III
IV
V
VI
VII
VIII
IX
X
XI
XII
XIII
XIV
XV
XVI
Chromosome
replicate 1
replicate 2
significance threshold
Multipool LOD
30
25
20
15
10
5
0
I
II
III
IV
V
VI
VII
VIII
IX
X
XI
XII
XIII
XIV
XV
XVI
Chromosome

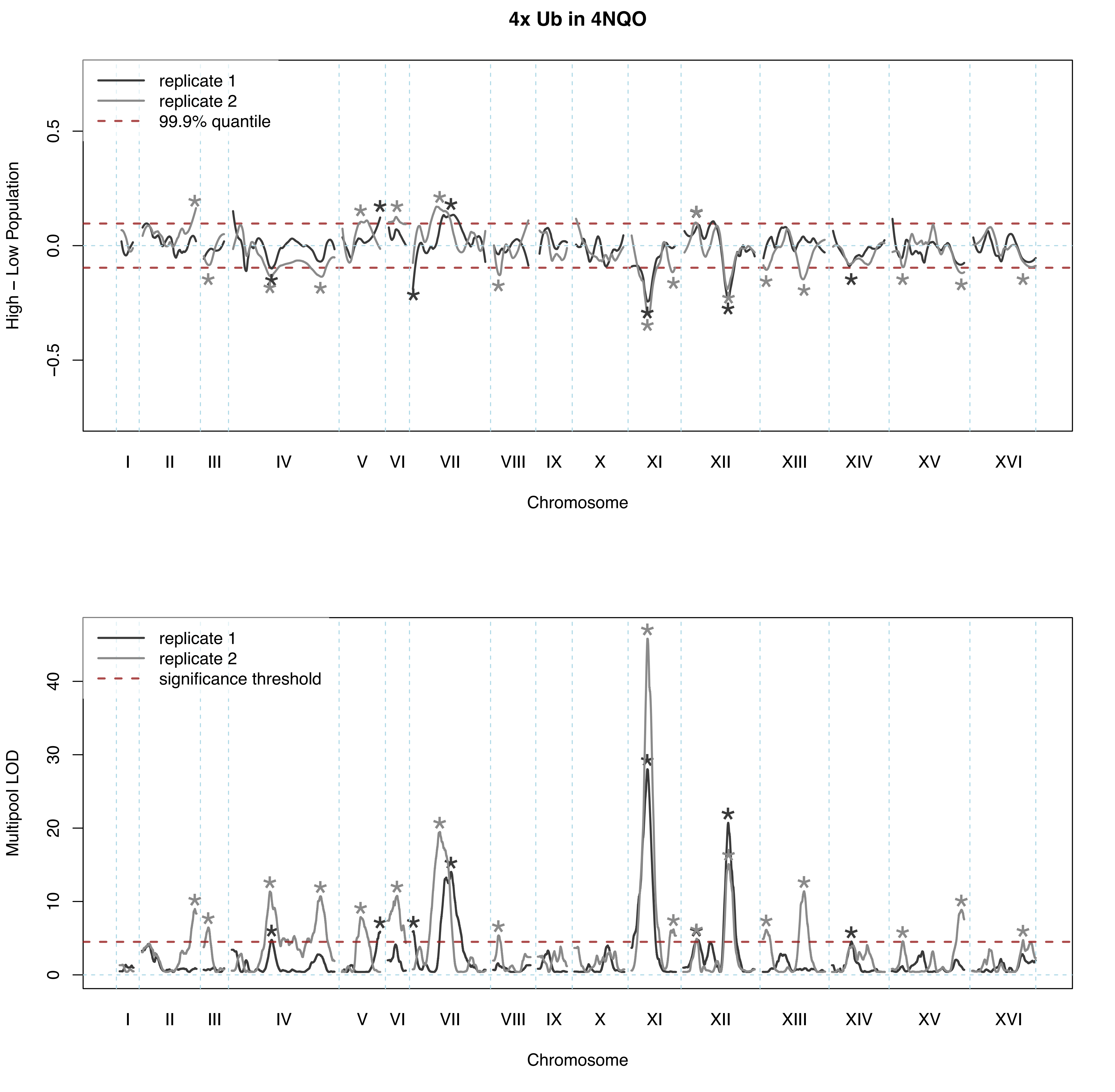

4x Ub in 4NQO
replicate 1
replicate 2
99.9% quantile
0.5
0.0
−0.5
High − Low Population
I
II
III
IV
V
VI
VII
VIII
IX
X
XI
XII
XIII
XIV
XV
XVI
Chromosome
replicate 1
replicate 2
significance threshold
40
30
20
10
0
Multipool LOD
I
II
III
IV
V
VI
VII
VIII
IX
X
XI
XII
XIII
XIV
XV
XVI
Chromosome

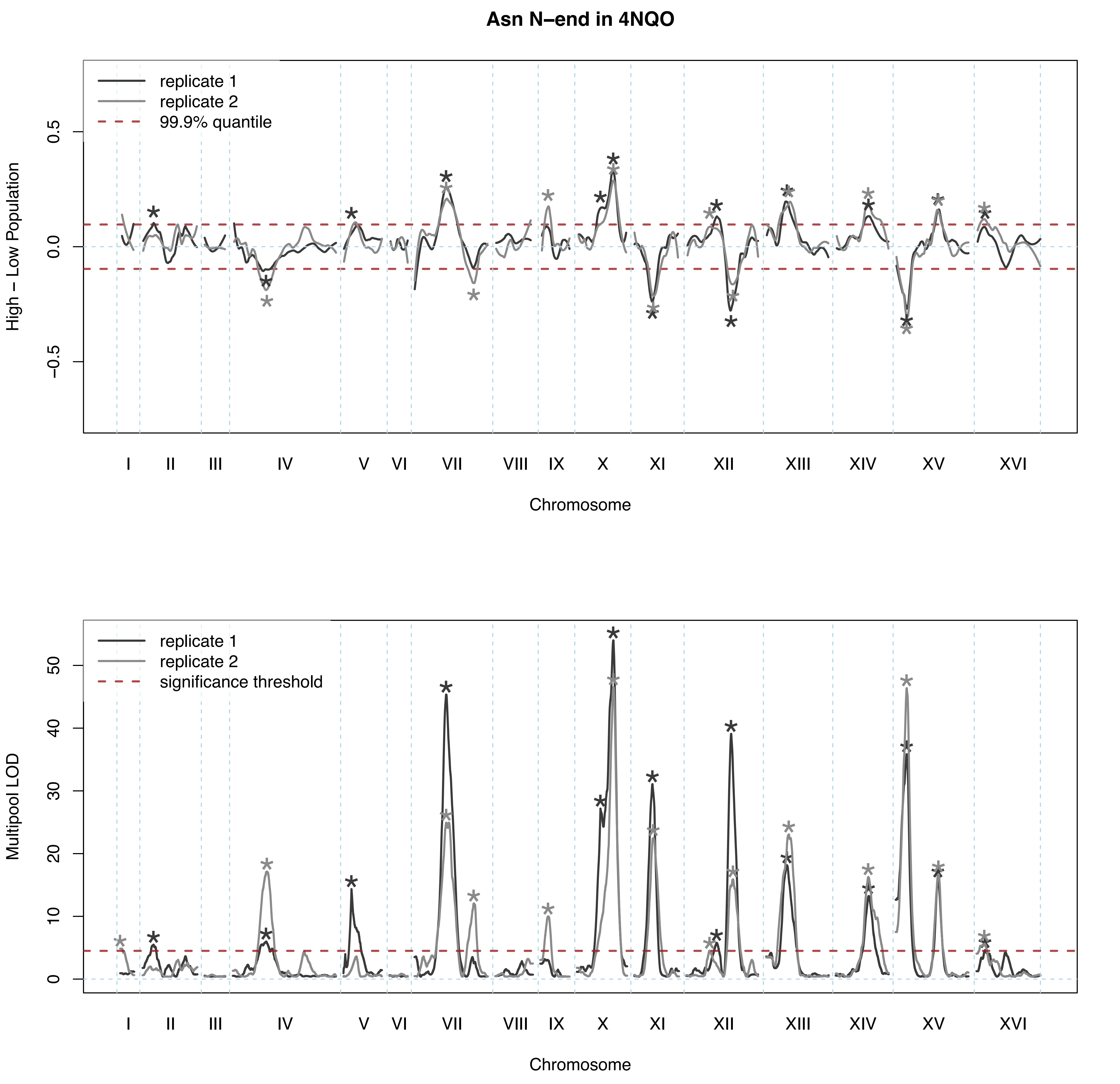
Asn N−end in 4NQO
replicate 1
replicate 2
99.9% quantile
High − Low Population
0.5
0.0
−0.5
I
II
III
IV
V
VI
VII
VIII
IX
X
XI
XII
XIII
XIV
XV
XVI
Chromosome
replicate 1
replicate 2
significance threshold
Multipool LOD
0
10
20
30
40
50
Chromosome

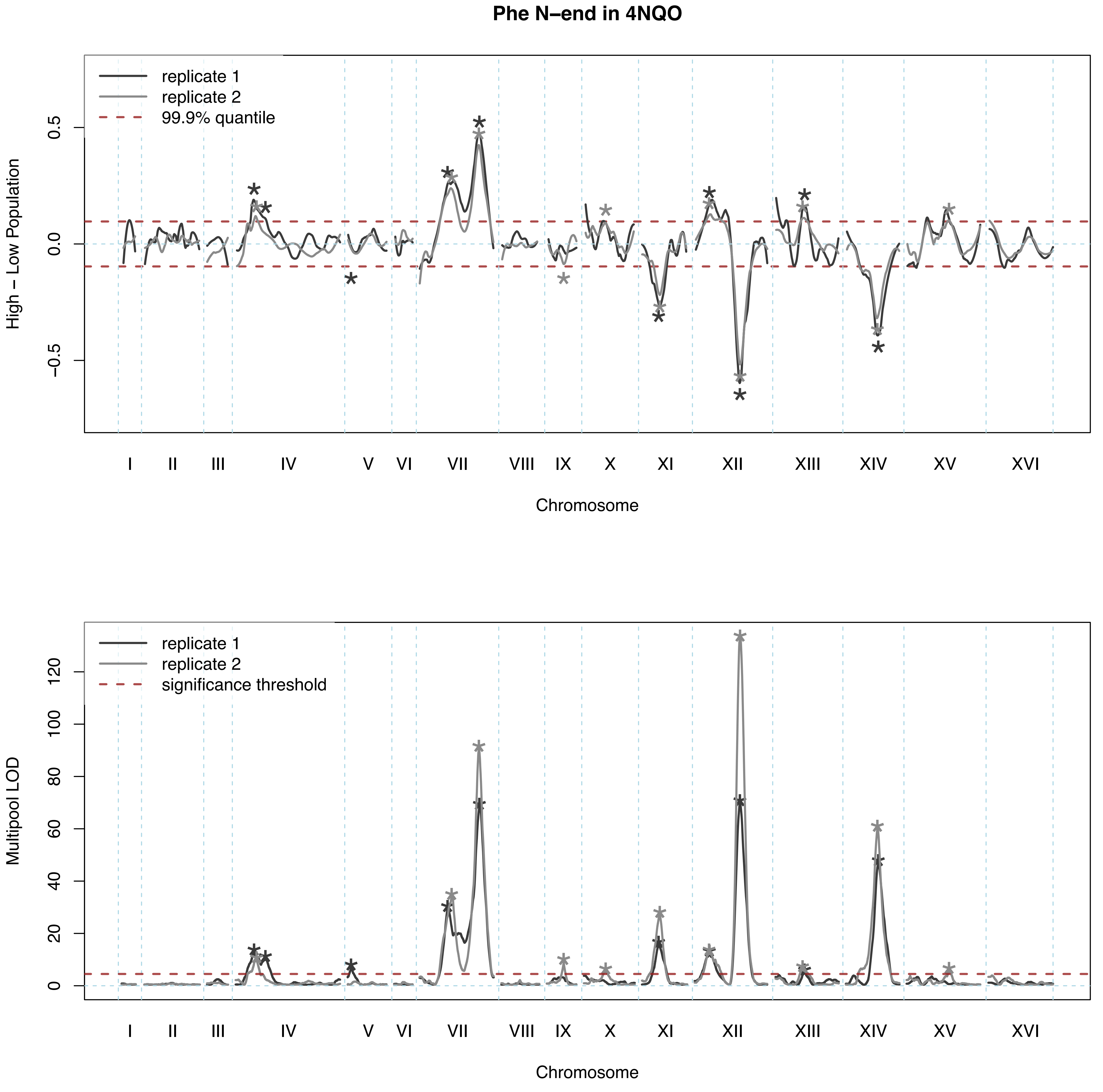
Phe N–end in 4NQO
replicate 1
replicate 2
99.9% quantile
High – Low Population
0.5
0.0
−0.5
I
II
III
IV
V
VI
VII
VIII
IX
X
XI
XII
XIII
XIV
XV
XVI
Chromosome
replicate 1
replicate 2
significance threshold
Multipool LOD
120
100
80
60
40
20
0
I
II
III
IV
V
VI
VII
VIII
IX
X
XI
XII
XIII
XIV
XV
XVI
Chromosome

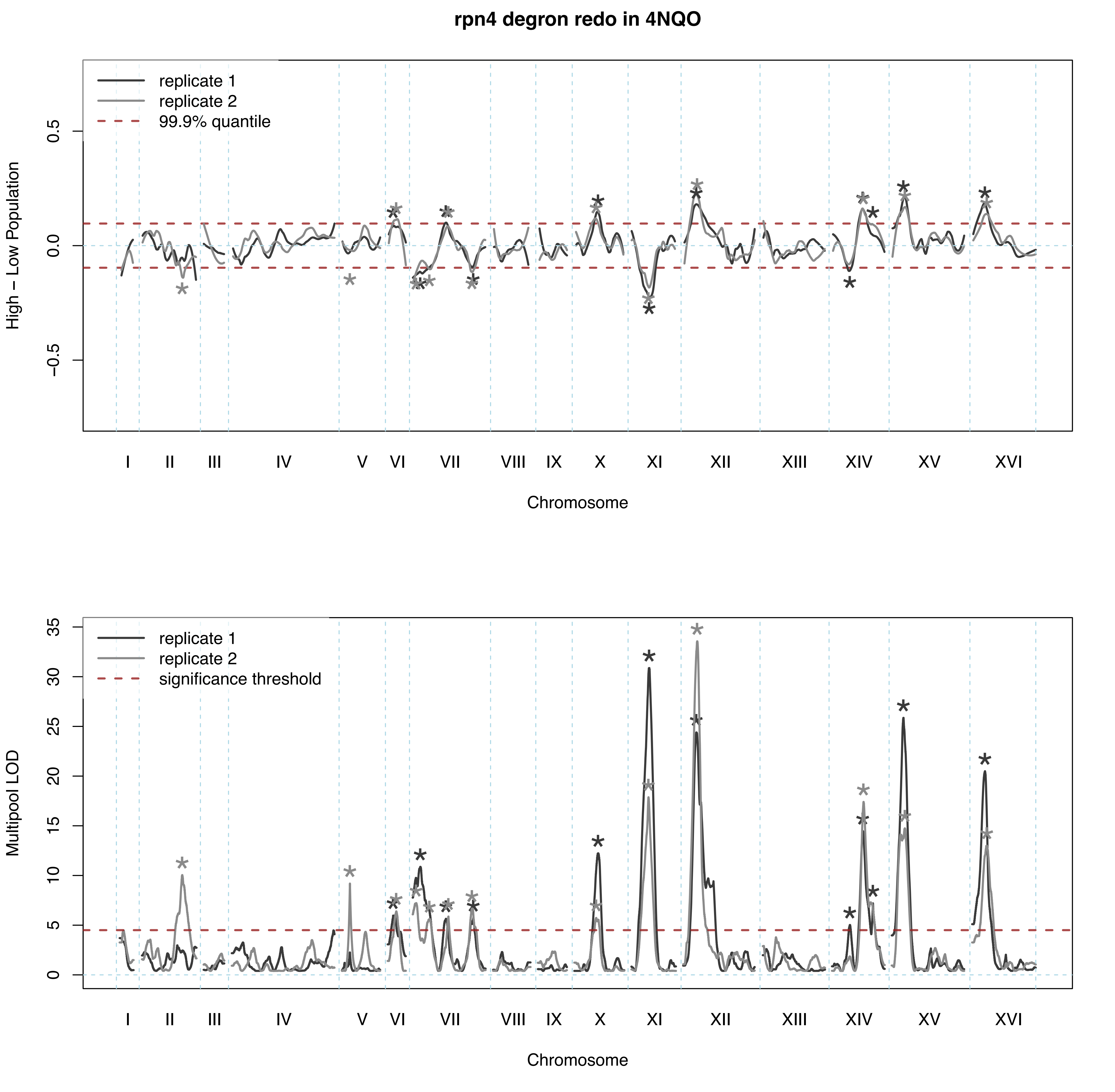

rpn4 degron redo in 4NQO
replicate 1
replicate 2
99.9% quantile
High – Low Population
0.5
0.0
−0.5
I
II
III
IV
V
VI
VII
VIII
IX
X
XI
XII
XIII
XIV
XV
XVI
Chromosome
replicate 1
replicate 2
significance threshold
Multipool LOD
35
30
25
20
15
10
5
0
I
II
III
IV
V
VI
VII
VIII
IX
X
XI
XII
XIII
XIV
XV
XVI
Chromosome

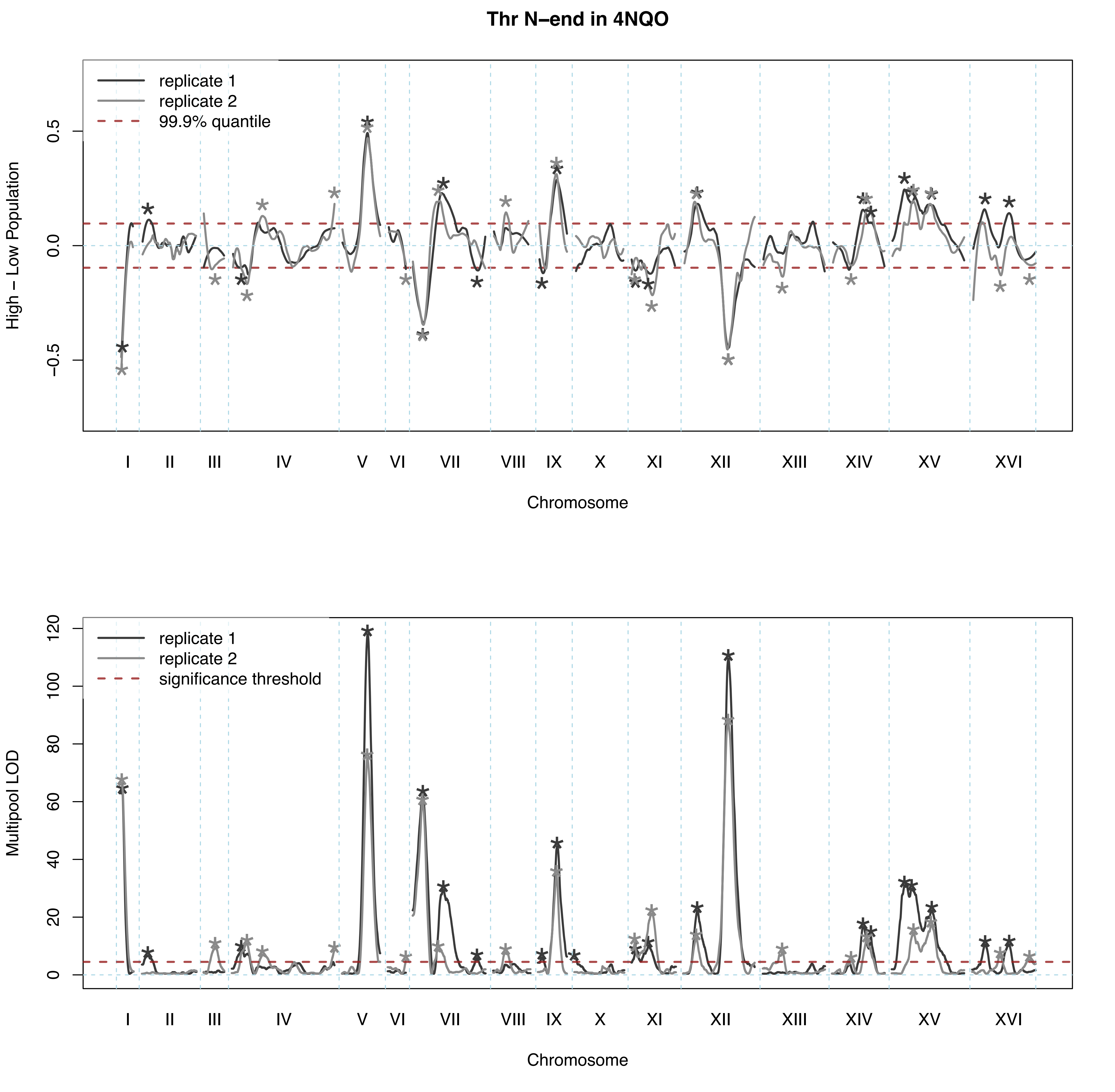

Thr N–end in 4NQO
replicate 1
replicate 2
99.9% quantile
High – Low Population
0.5
0.0
−0.5
I
II
III
IV
V
VI
VII
VIII
IX
X
XI
XII
XIII
XIV
XV
XVI
Chromosome
replicate 1
replicate 2
significance threshold
Multipool LOD
0
20
40
60
80
100
120
I
II
III
IV
V
VI
VII
VIII
IX
X
XI
XII
XIII
XIV
XV
XVI
Chromosome

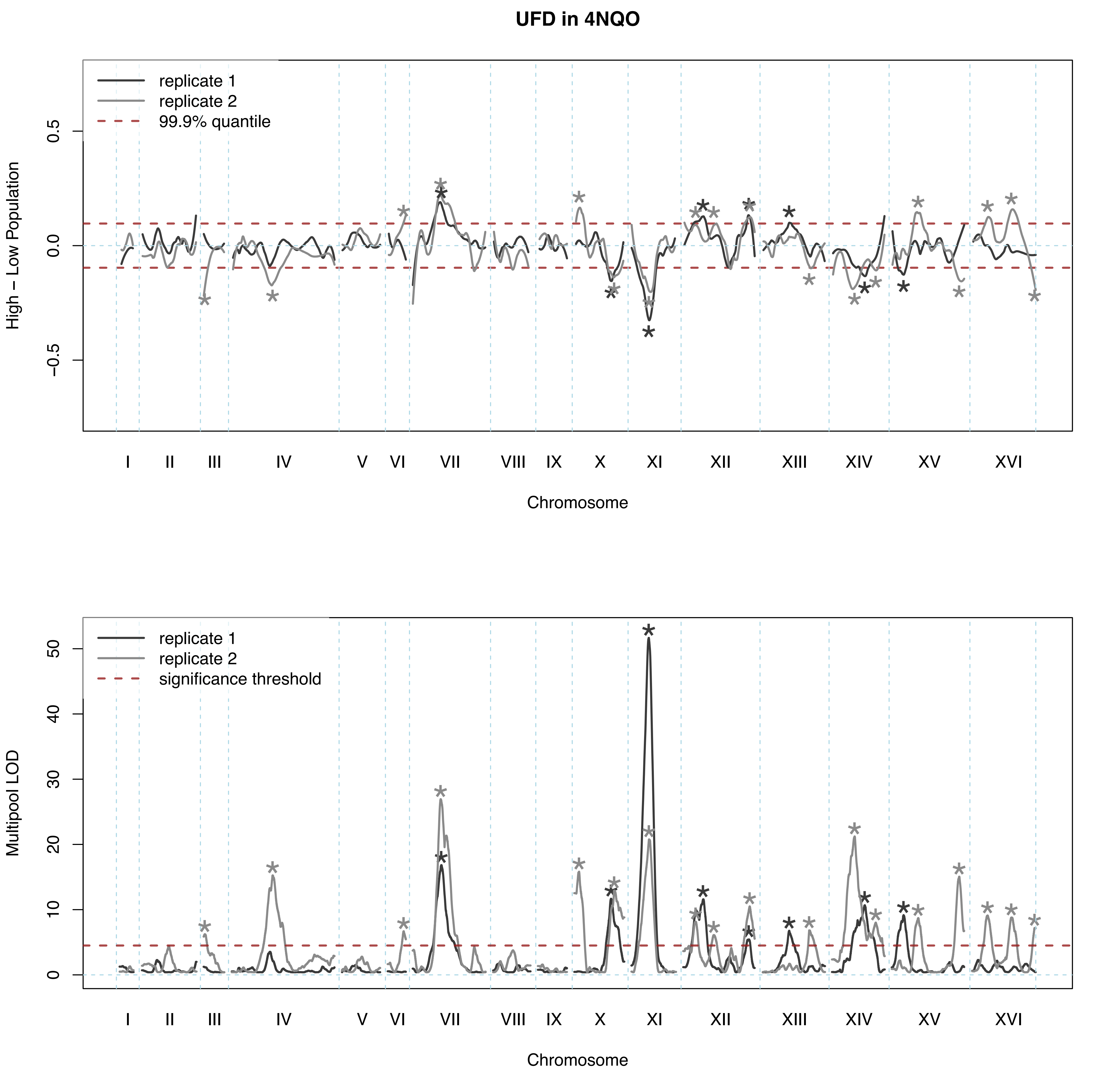

UFD in 4NQO
replicate 1
replicate 2
99.9% quantile
High – Low Population
0.5
0.0
−0.5
I
II
III
IV
V
VI
VII
VIII
IX
X
XI
XII
XIII
XIV
XV
XVI
Chromosome
replicate 1
replicate 2
significance threshold
Multipool LOD
50
40
30
20
10
0
Chromosome

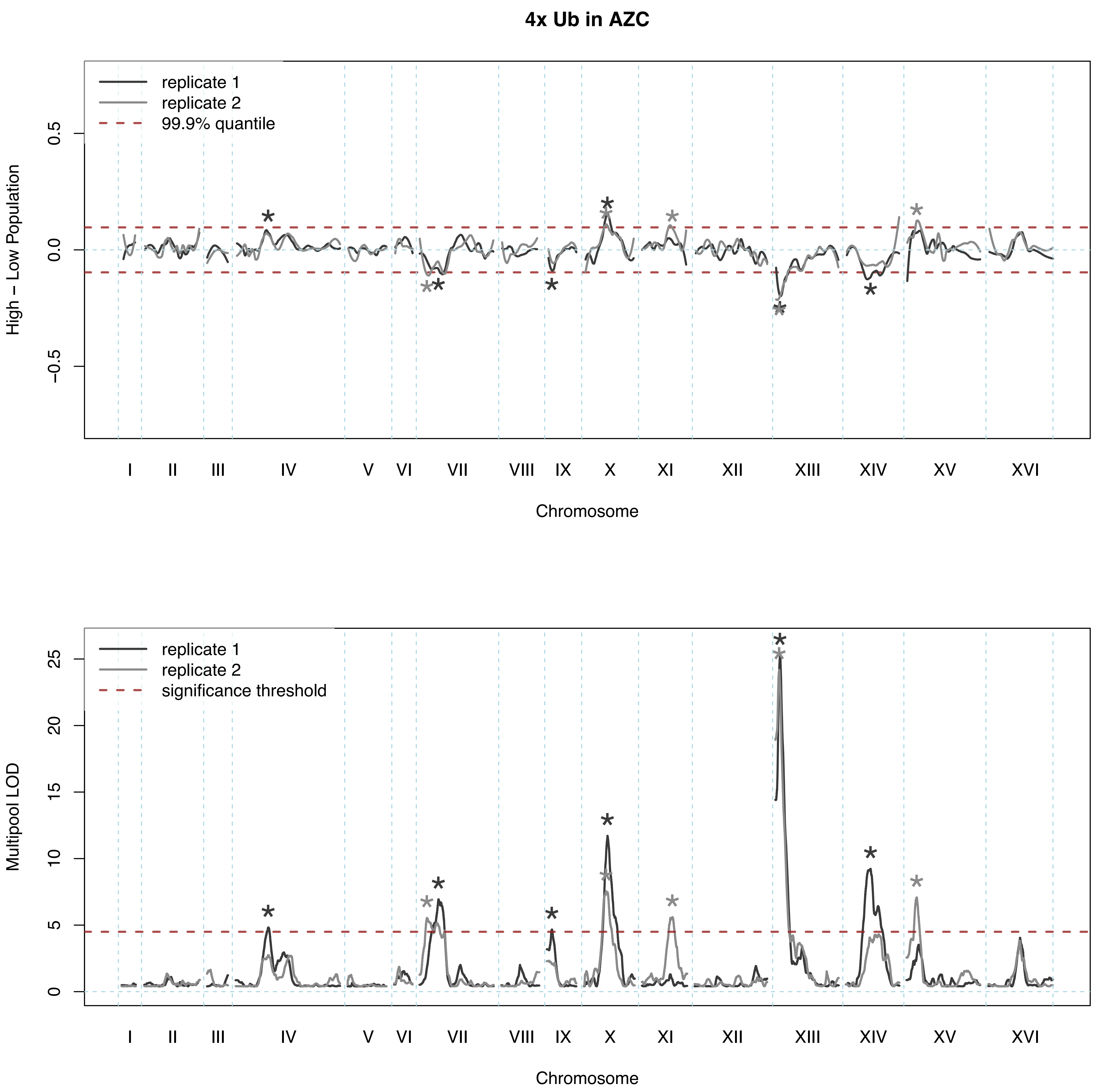
4x Ub in AZC
replicate 1
replicate 2
99.9% quantile
High – Low Population
0.5
0.0
−0.5
I
II
III
IV
V
VI
VII
VIII
IX
X
XI
XII
XIII
XIV
XV
XVI
Chromosome
replicate 1
replicate 2
significance threshold
Multipool LOD
25
20
15
10
5
0
I
II
III
IV
V
VI
VII
VIII
IX
X
XI
XII
XIII
XIV
XV
XVI
Chromosome

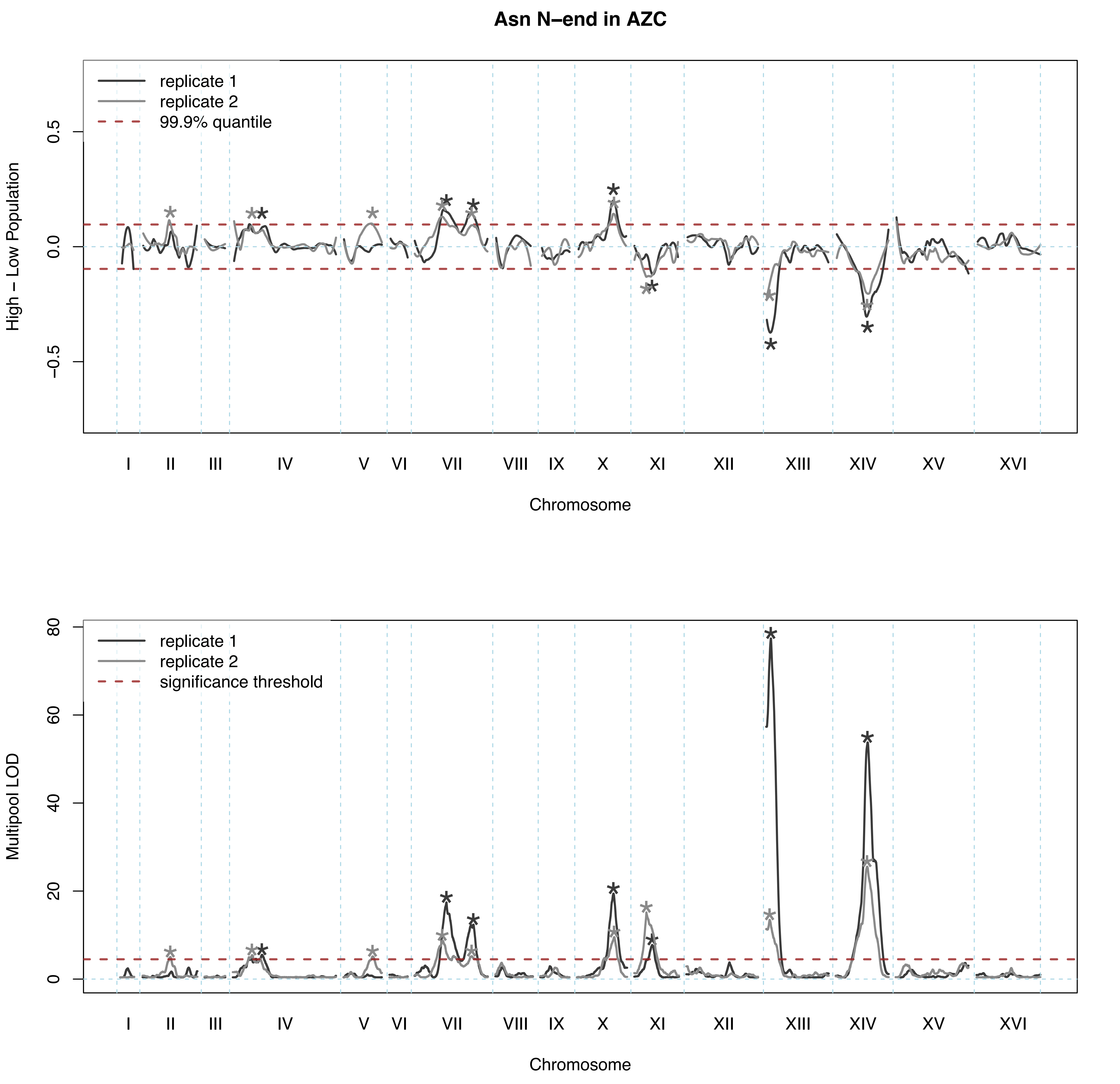
Asn N–end in AZC
replicate 1
replicate 2
99.9% quantile
High – Low Population
0.5
0.0
−0.5
I
II
III
IV
V
VI
VII
VIII
IX
X
XI
XII
XIII
XIV
XV
XVI
Chromosome
replicate 1
replicate 2
significance threshold
Multipool LOD
80
60
40
20
0
Chromosome

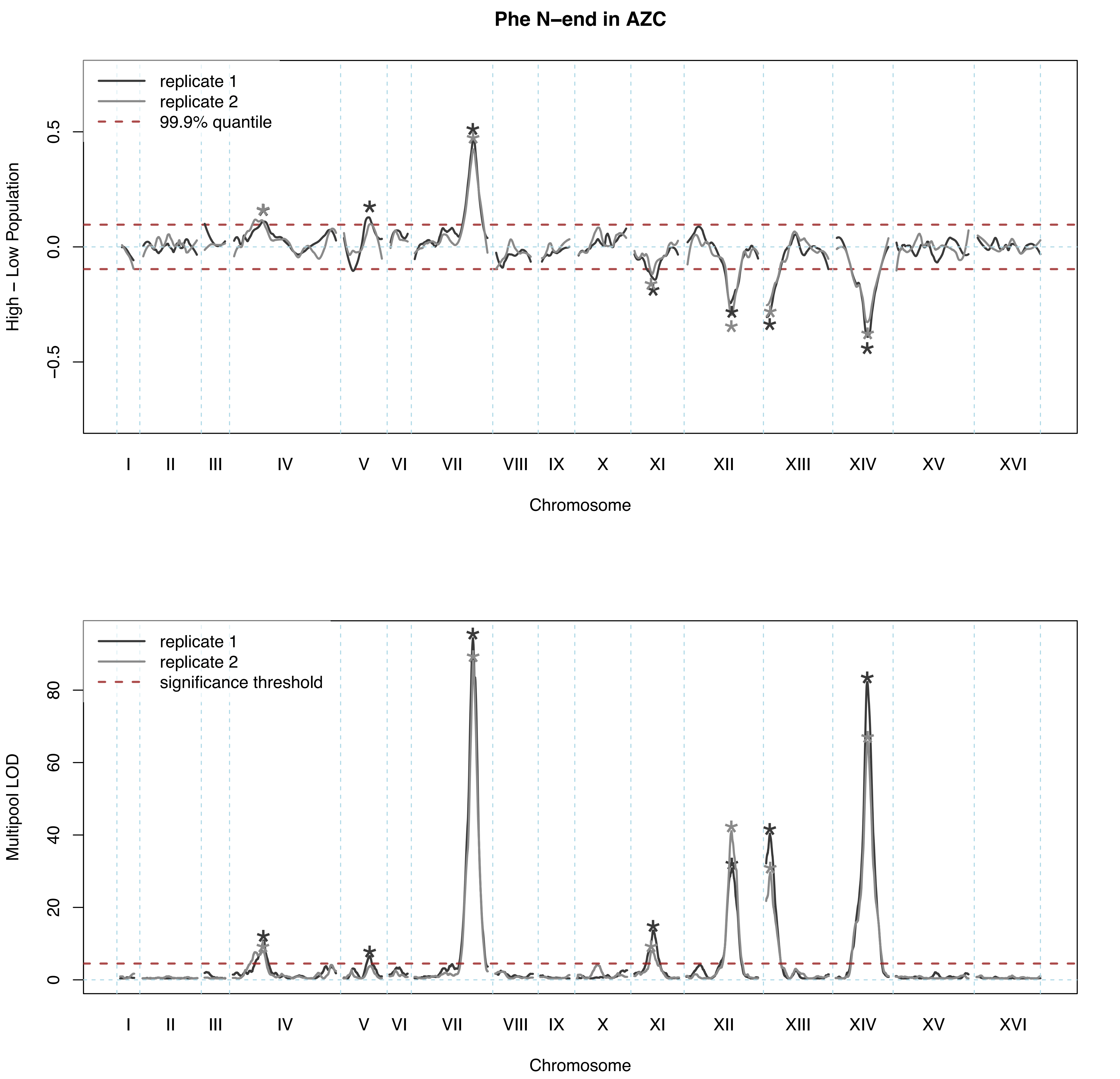

Phe N–end in AZC
replicate 1
replicate 2
99.9% quantile
High – Low Population
0.5
0.0
−0.5
I
II
III
IV
V
VI
VII
VIII
IX
X
XI
XII
XIII
XIV
XV
XVI
Chromosome
replicate 1
replicate 2
significance threshold
Multipool LOD
80
60
40
20
0
I
II
III
IV
V
VI
VII
VIII
IX
X
XI
XII
XIII
XIV
XV
XVI
Chromosome

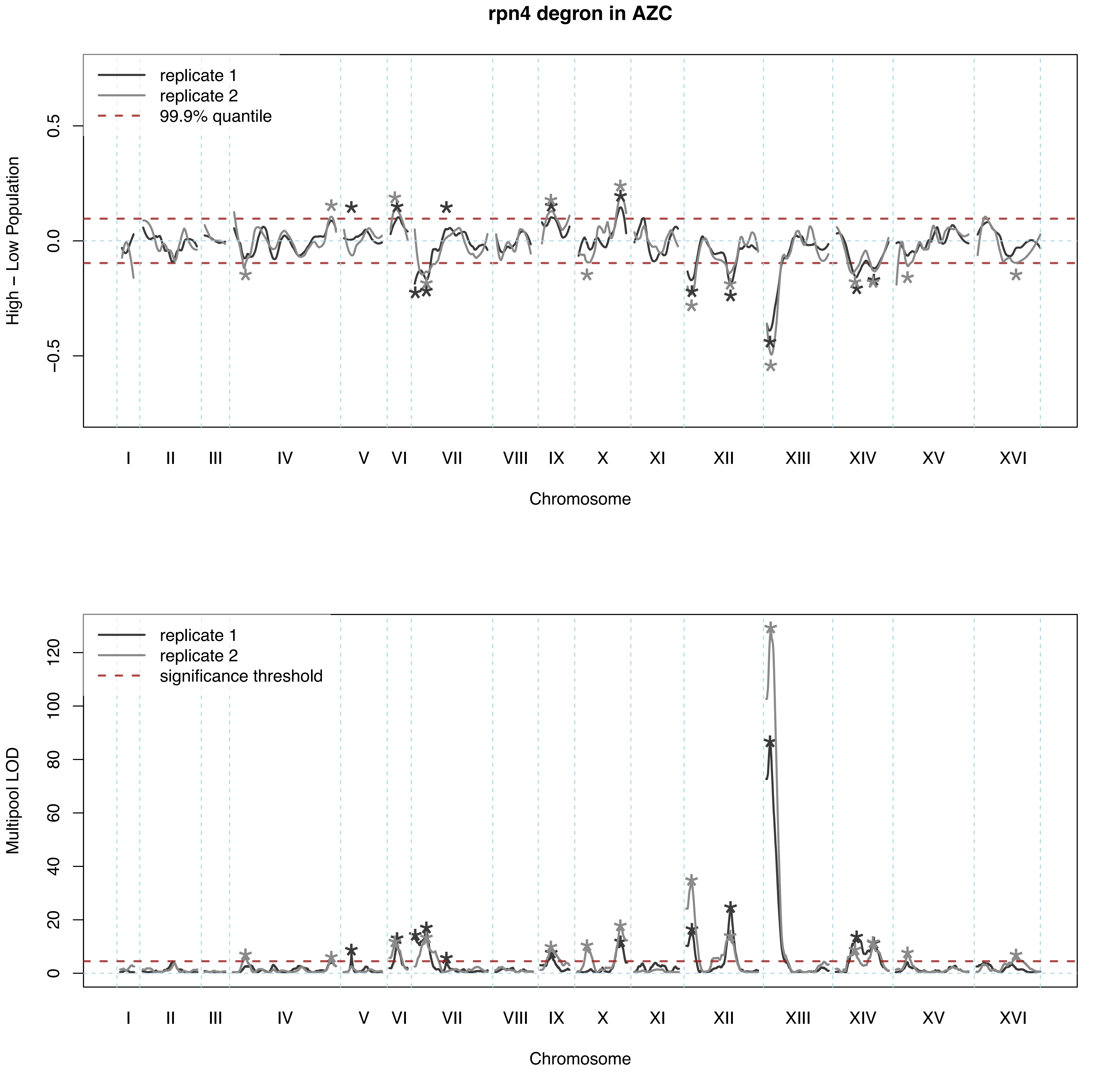
rpn4 degron in AZC
replicate 1
replicate 2
99.9% quantile
High – Low Population
0.5
0.0
−0.5
I
II
III
IV
V
VI
VII
VIII
IX
X
XI
XII
XIII
XIV
XV
XVI
Chromosome
replicate 1
replicate 2
significance threshold
Multipool LOD
120
100
80
60
40
20
0
I
II
III
IV
V
VI
VII
VIII
IX
X
XI
XII
XIII
XIV
XV
XVI
Chromosome

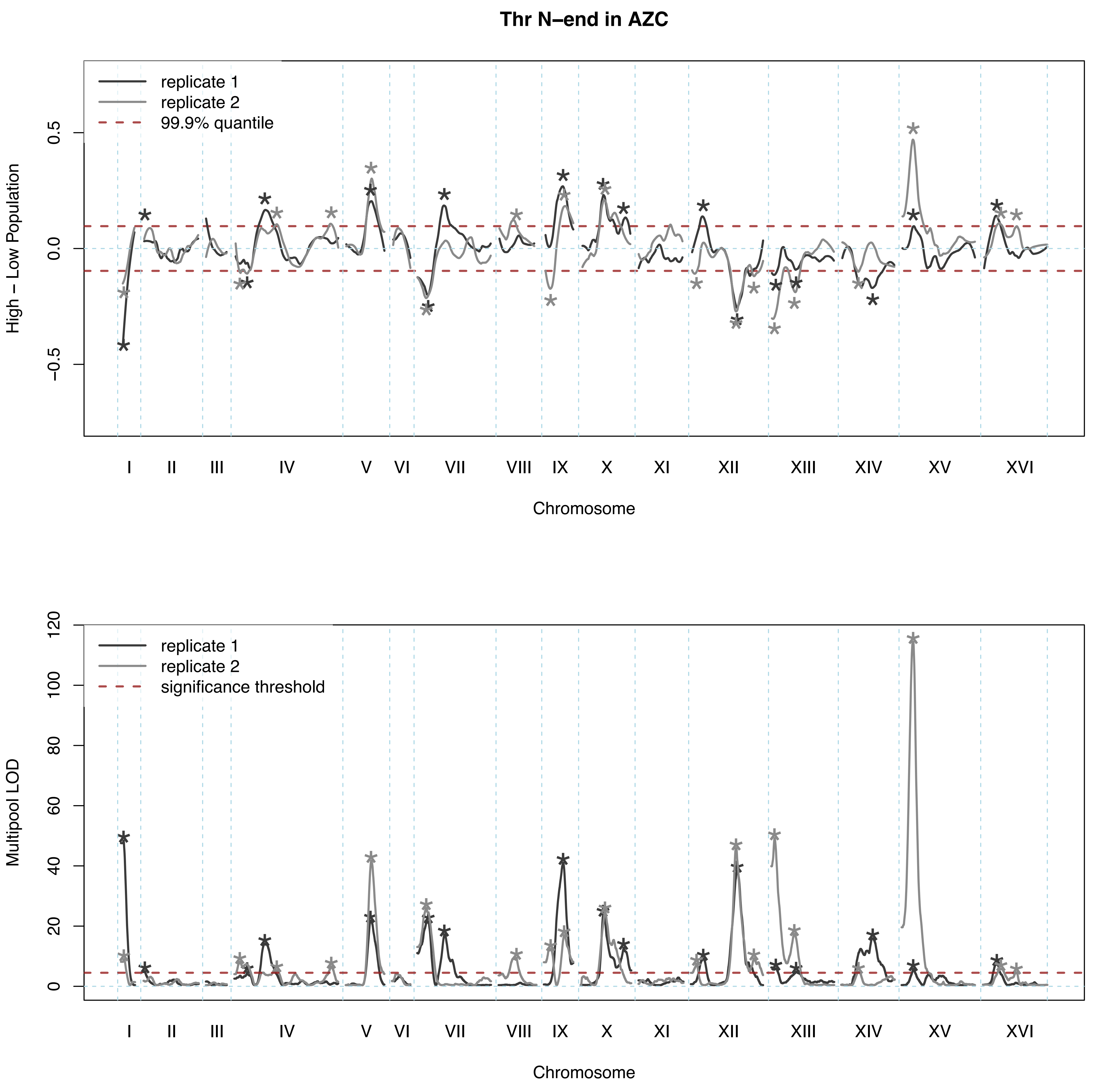
Thr N–end in AZC
replicate 1
replicate 2
99.9% quantile
High – Low Population
0.5
0.0
−0.5
I
II
III
IV
V
VI
VII
VIII
IX
X
XI
XII
XIII
XIV
XV
XVI
Chromosome
replicate 1
replicate 2
significance threshold
Multipool LOD
0
20
40
60
80
100
120
I
II
III
IV
V
VI
VII
VIII
IX
X
XI
XII
XIII
XIV
XV
XVI
Chromosome

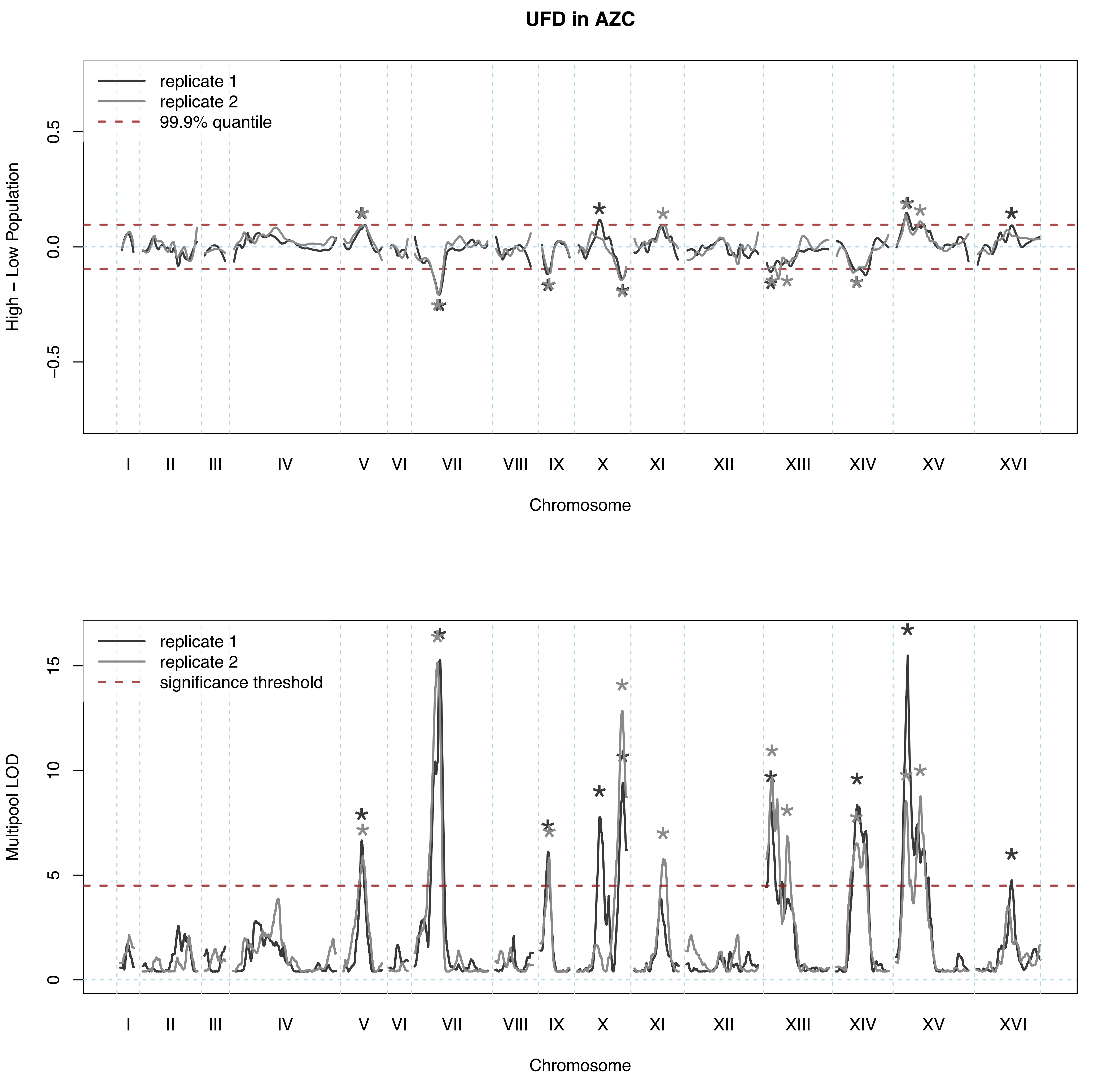
UFD in AZC
replicate 1
replicate 2
99.9% quantile
High – Low Population
0.5
0.0
−0.5
I
II
III
IV
V
VI
VII
VIII
IX
X
XI
XII
XIII
XIV
XV
XVI
Chromosome
replicate 1
replicate 2
significance threshold
Multipool LOD
15
10
5
0
I
II
III
IV
V
VI
VII
VIII
IX
X
XI
XII
XIII
XIV
XV
XVI
Chromosome

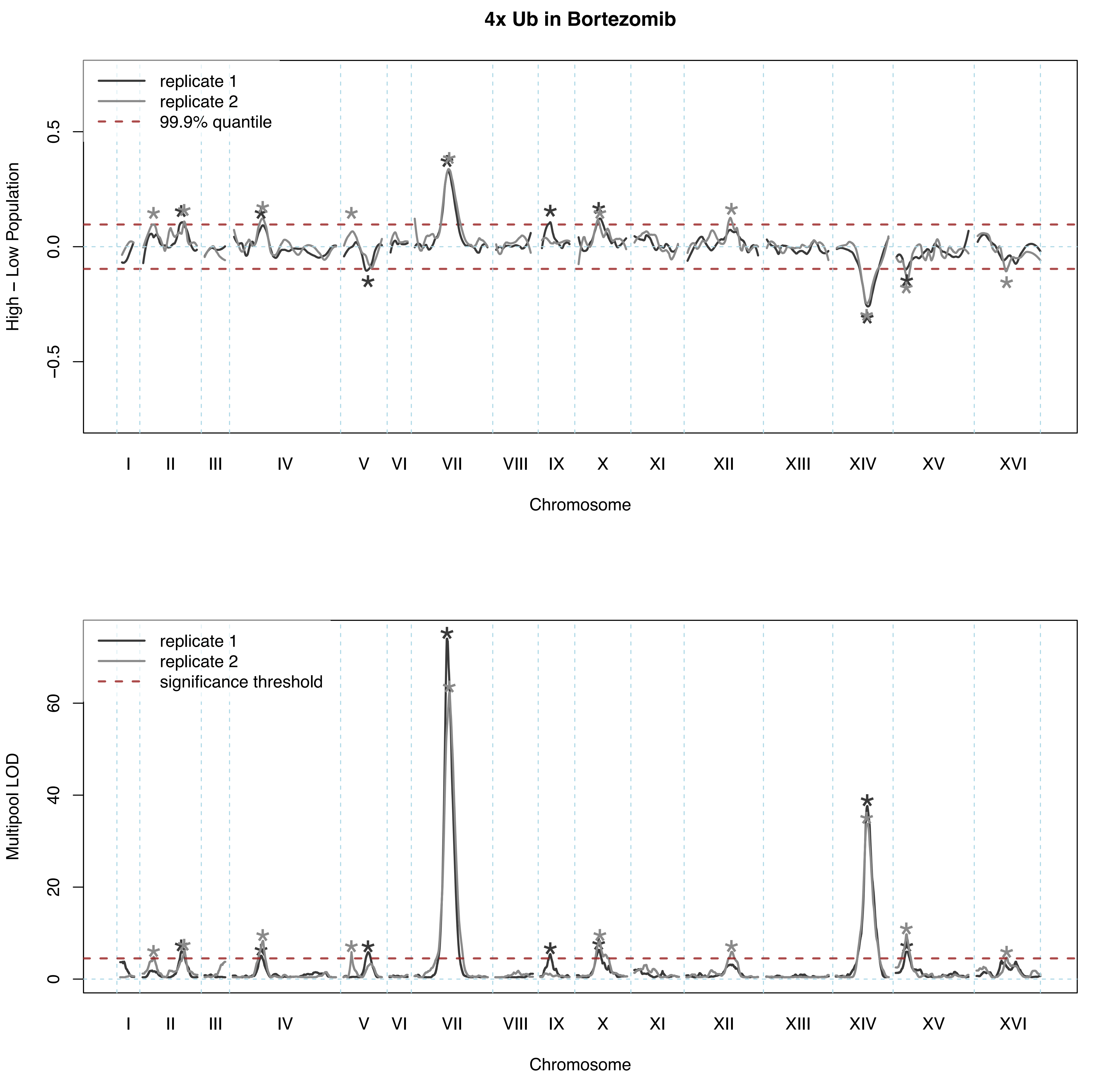
4x Ub in Bortezomib
replicate 1
replicate 2
99.9% quantile
High − Low Population
0.5
0.0
−0.5
I
II
III
IV
V
VI
VII
VIII
IX
X
XI
XII
XIII
XIV
XV
XVI
Chromosome
replicate 1
replicate 2
significance threshold
Multipool LOD
60
40
20
0
I
II
III
IV
V
VI
VII
VIII
IX
X
XI
XII
XIII
XIV
XV
XVI
Chromosome

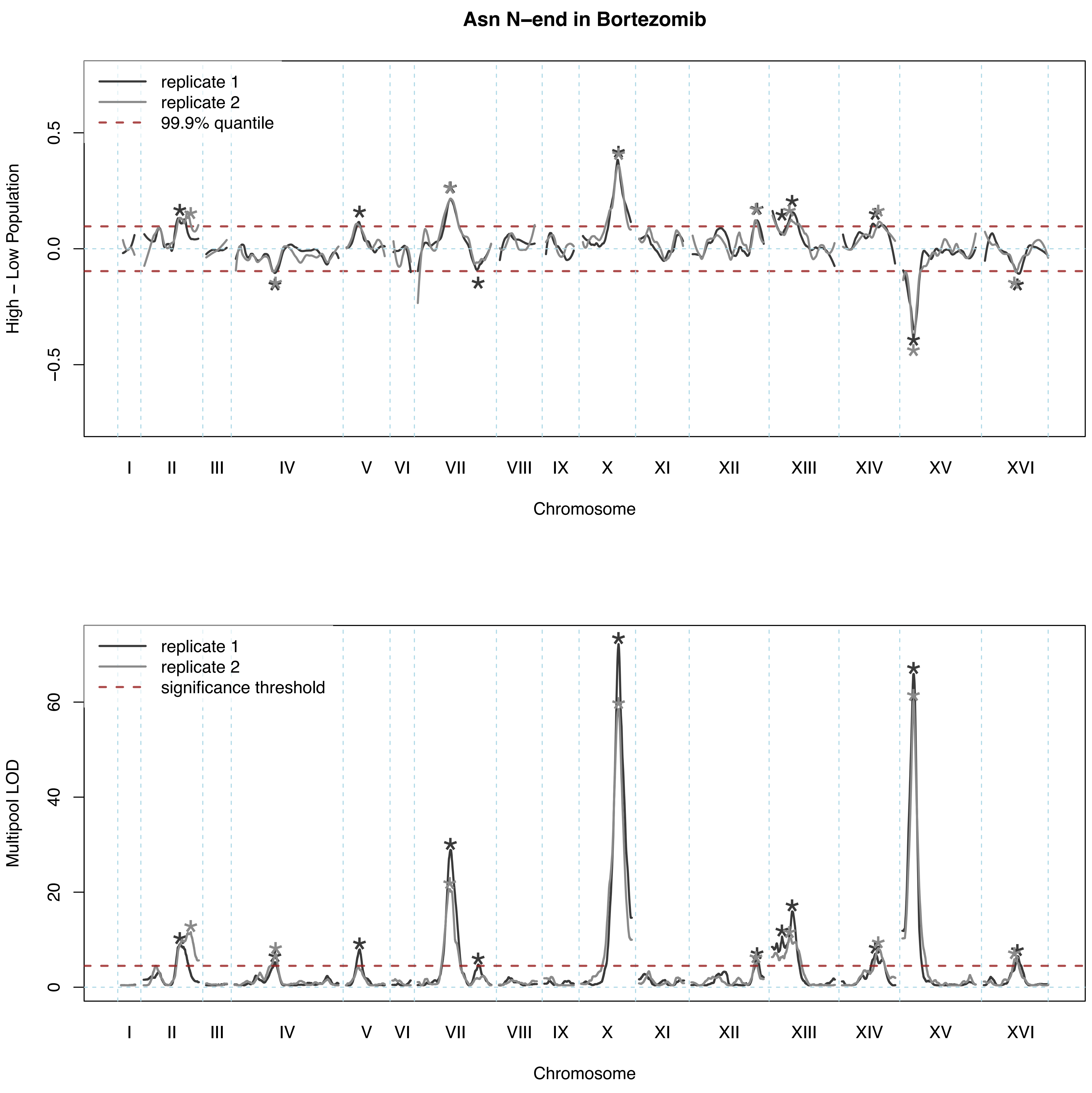
Asn N–end in Bortezomib
replicate 1
replicate 2
99.9% quantile
High – Low Population
0.5
0.0
−0.5
I
II
III
IV
V
VI
VII
VIII
IX
X
XI
XII
XIII
XIV
XV
XVI
Chromosome
replicate 1
replicate 2
significance threshold
Multipool LOD
60
40
20
0
I
II
III
IV
V
VI
VII
VIII
IX
X
XI
XII
XIII
XIV
XV
XVI
Chromosome

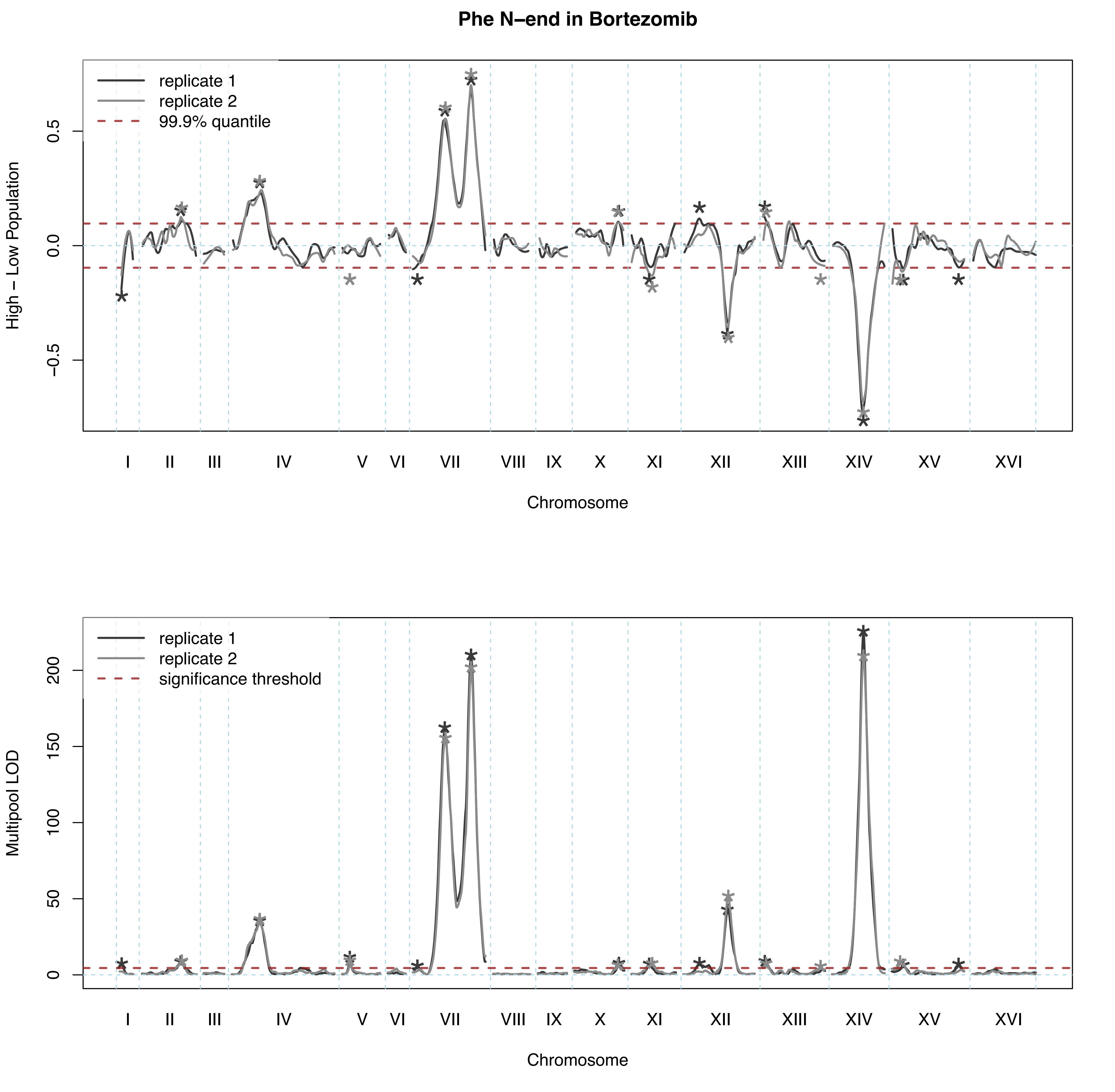

Phe N–end in Bortezomib
replicate 1
replicate 2
99.9% quantile
High – Low Population
0.5
0.0
−0.5
I
II
III
IV
V
VI
VII
VIII
IX
X
XI
XII
XIII
XIV
XV
XVI
Chromosome
replicate 1
replicate 2
significance threshold
Multipool LOD
0
50
100
150
200
I
II
III
IV
V
VI
VII
VIII
IX
X
XI
XII
XIII
XIV
XV
XVI
Chromosome

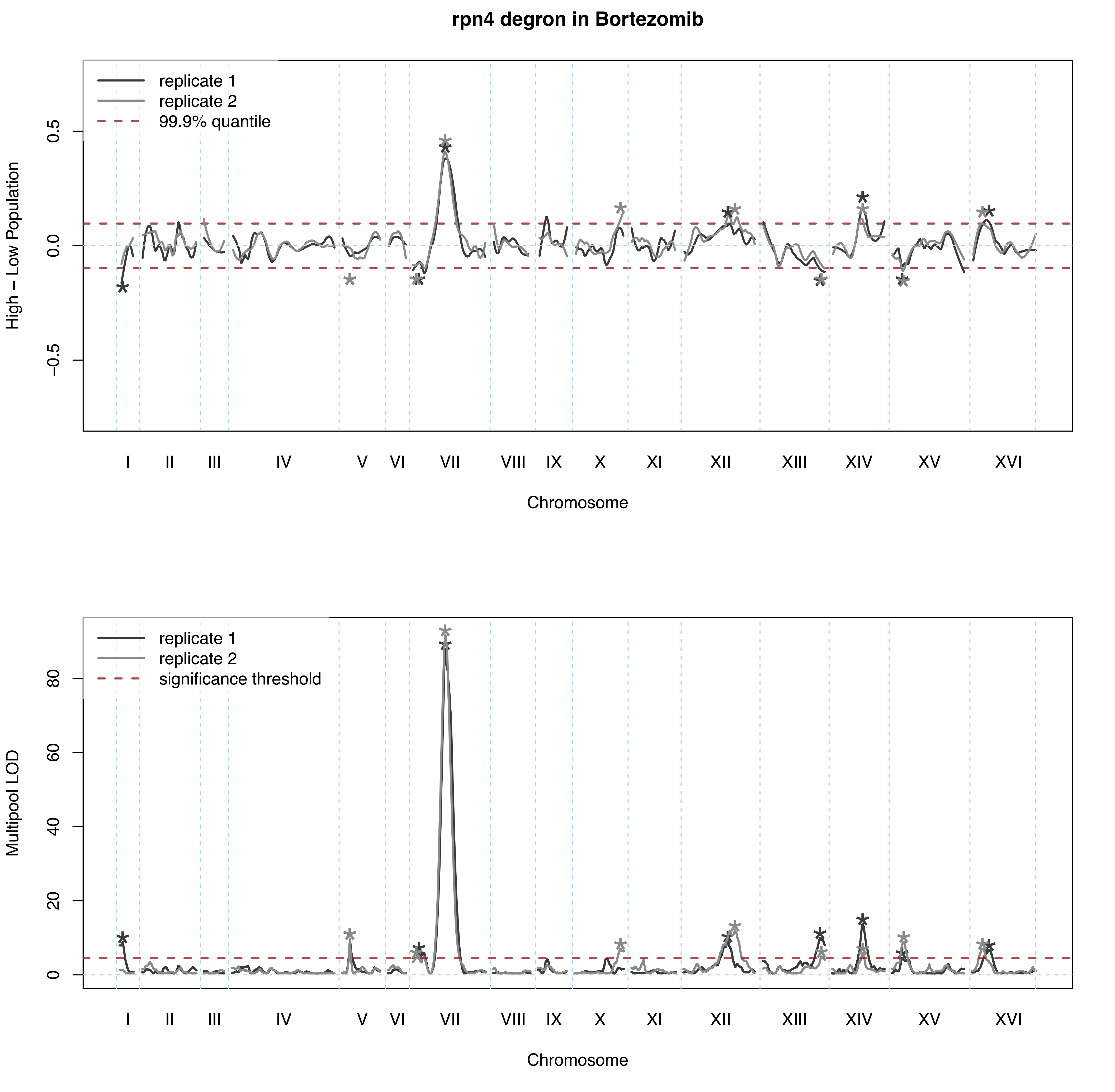

rpn4 degron in Bortezomib
replicate 1
replicate 2
99.9% quantile
High – Low Population
0.5
0.0
−0.5
I
II
III
IV
V
VI
VII
VIII
IX
X
XI
XII
XIII
XIV
XV
XVI
Chromosome
replicate 1
replicate 2
significance threshold
Multipool LOD
80
60
40
20
0
I
II
III
IV
V
VI
VII
VIII
IX
X
XI
XII
XIII
XIV
XV
XVI
Chromosome

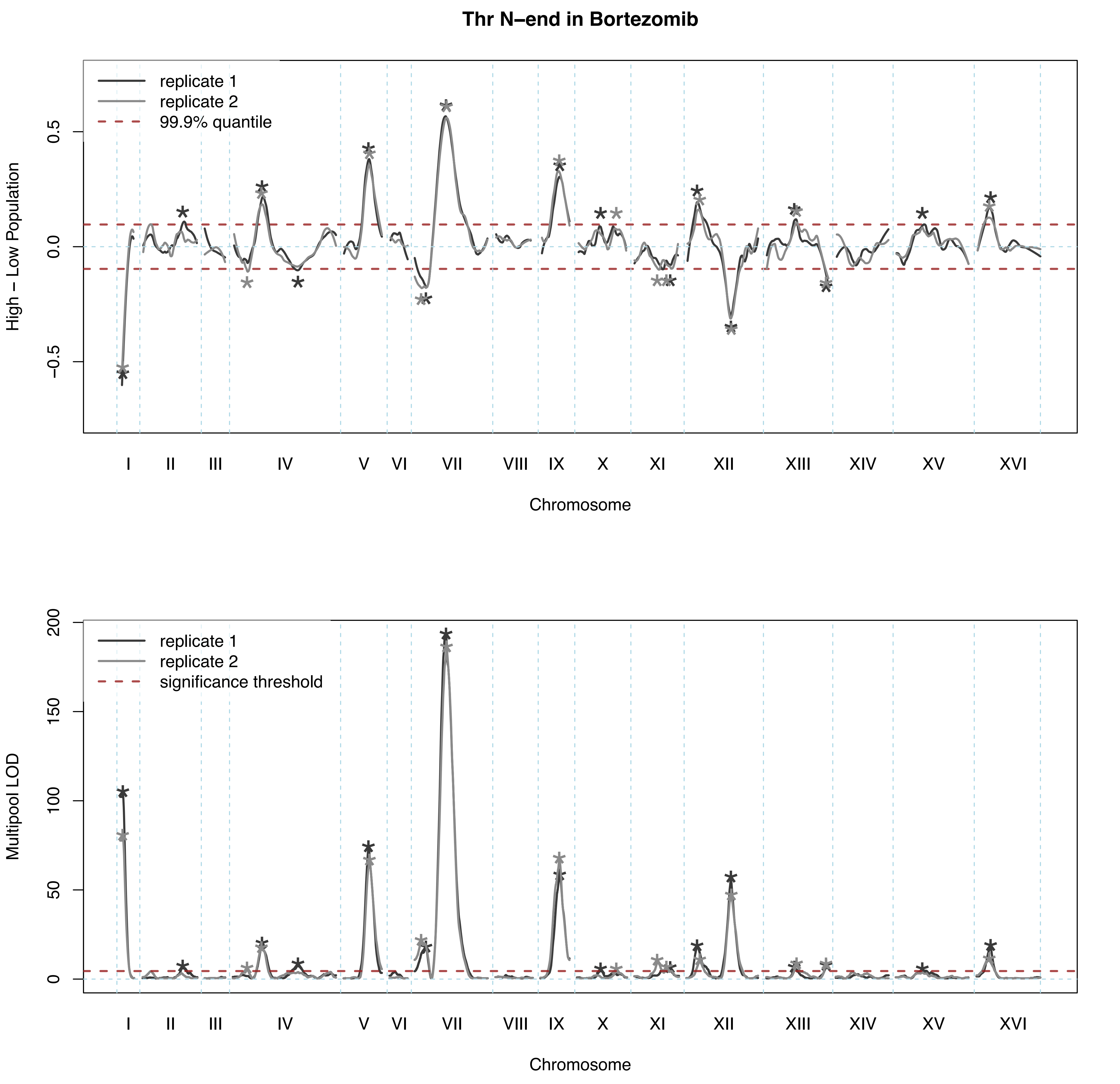

Thr N−end in Bortezomib
replicate 1
replicate 2
99.9% quantile
High − Low Population
0.5
0.0
−0.5
I
II
III
IV
V
VI
VII
VIII
IX
X
XI
XII
XIII
XIV
XV
XVI
Chromosome
replicate 1
replicate 2
significance threshold
Multipool LOD
200
150
100
50
0
I
II
III
IV
V
VI
VII
VIII
IX
X
XI
XII
XIII
XIV
XV
XVI
Chromosome

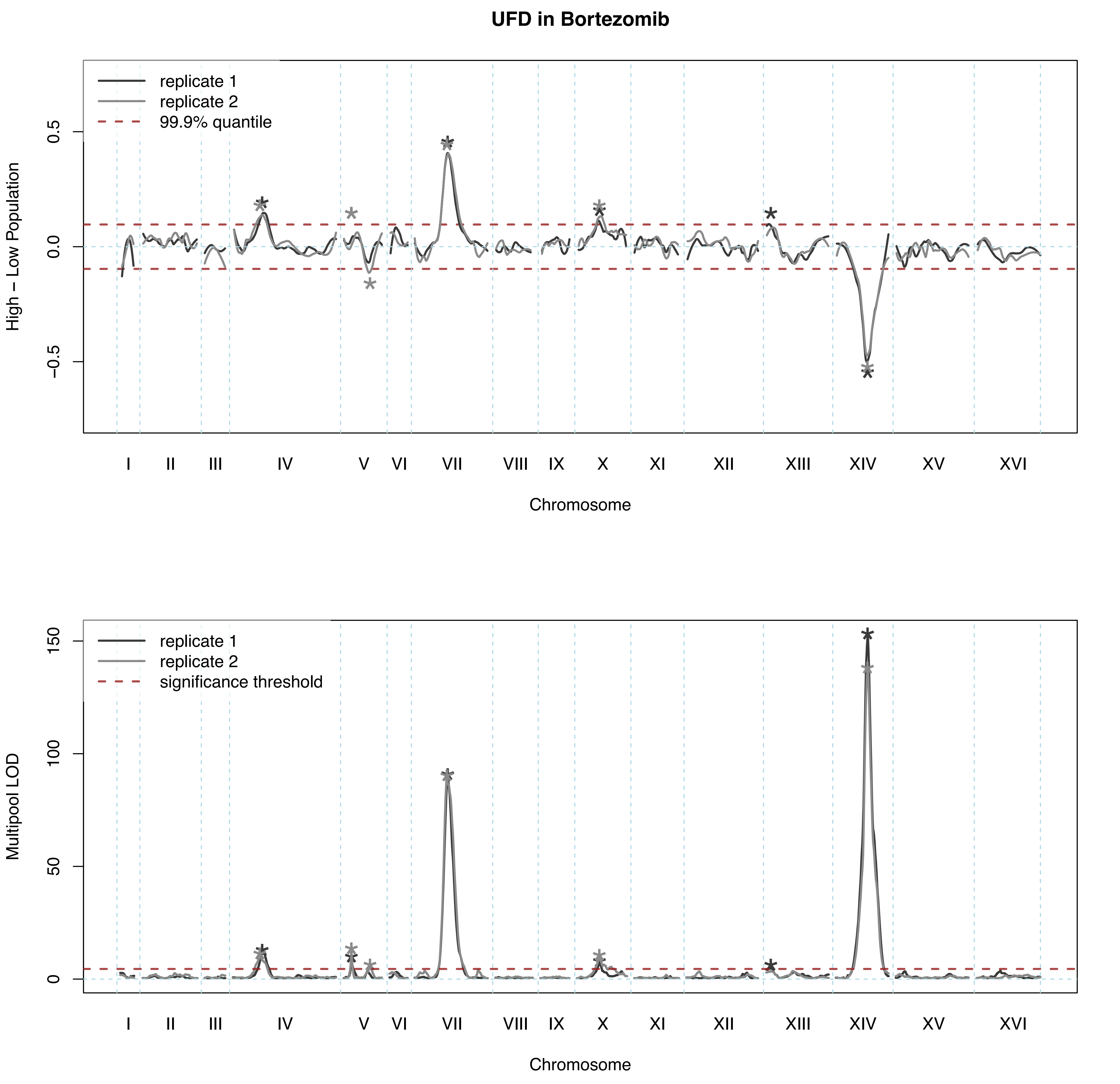
UFD in Bortezomib
replicate 1
replicate 2
99.9% quantile
High – Low Population
0.5
0.0
−0.5
I
II
III
IV
V
VI
VII
VIII
IX
X
XI
XII
XIII
XIV
XV
XVI
Chromosome
replicate 1
replicate 2
significance threshold
Multipool LOD
150
100
50
0
I
II
III
IV
V
VI
VII
VIII
IX
X
XI
XII
XIII
XIV
XV
XVI
Chromosome

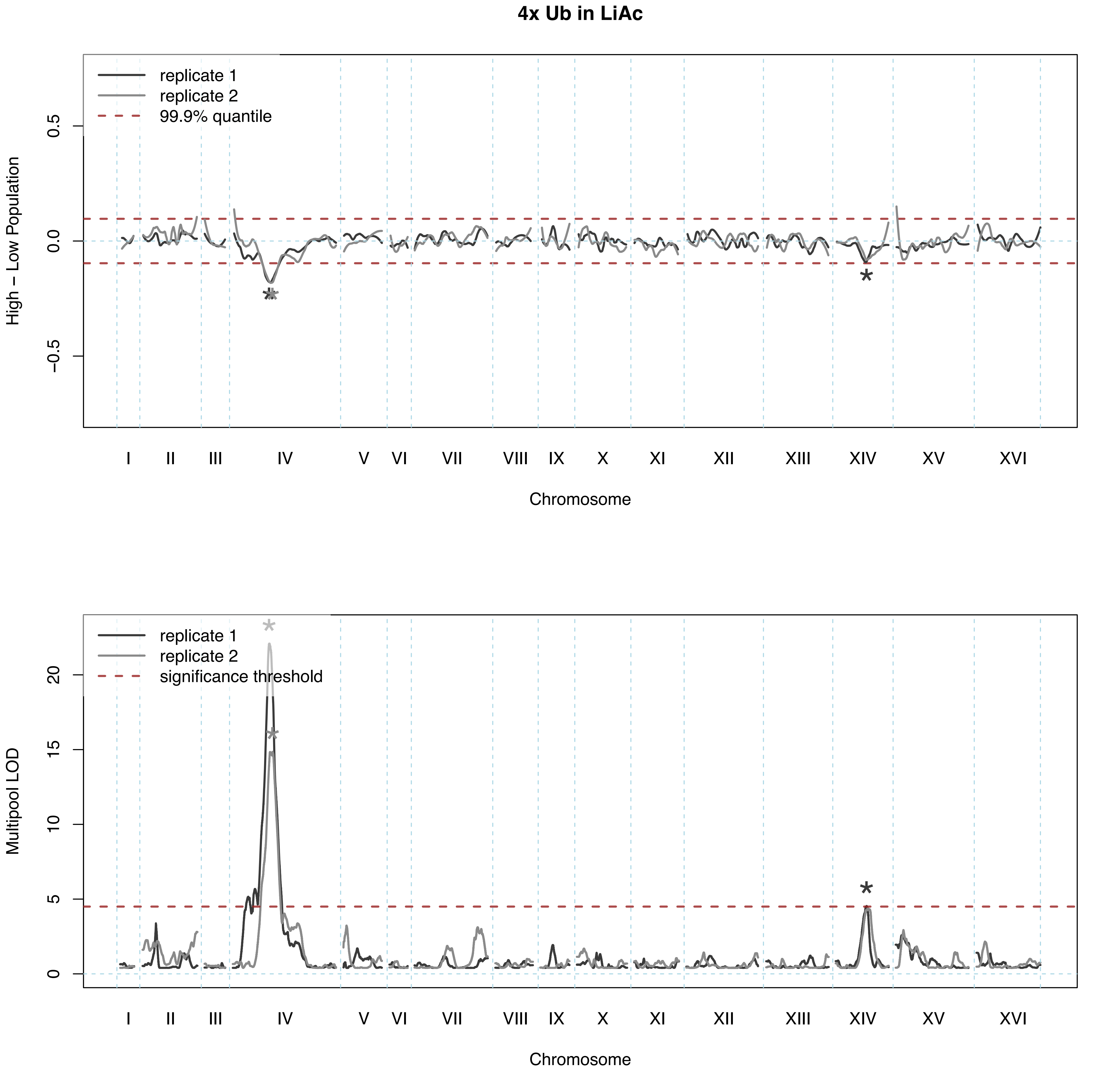
4x Ub in LiAc
replicate 1
replicate 2
99.9% quantile
High – Low Population
0.5
0.0
−0.5
I
II
III
IV
V
VI
VII
VIII
IX
X
XI
XII
XIII
XIV
XV
XVI
Chromosome
replicate 1
replicate 2
significance threshold
Multipool LOD
20
15
10
5
0
I
II
III
IV
V
VI
VII
VIII
IX
X
XI
XII
XIII
XIV
XV
XVI
Chromosome

**Asn N–end in LiAc**

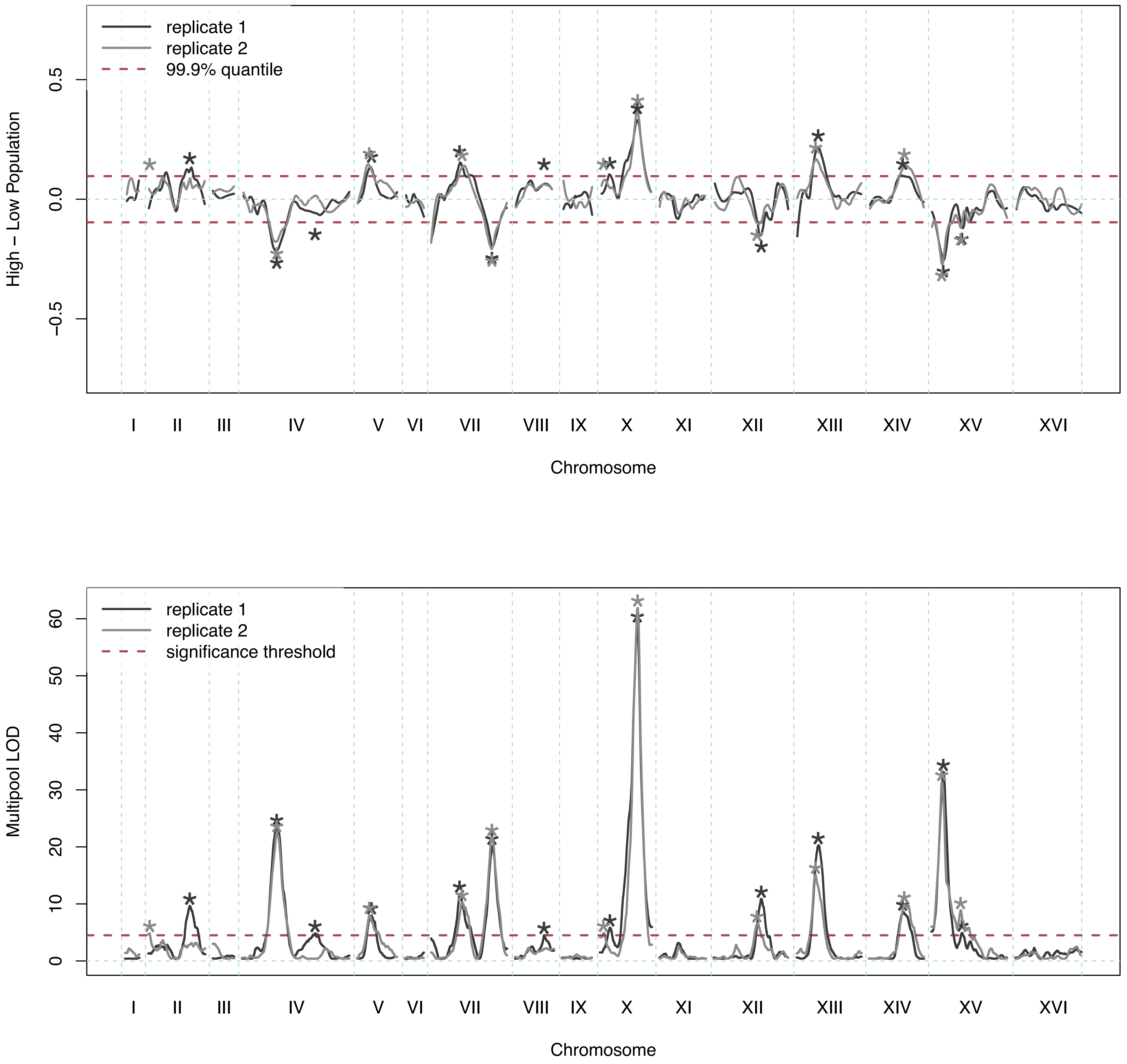

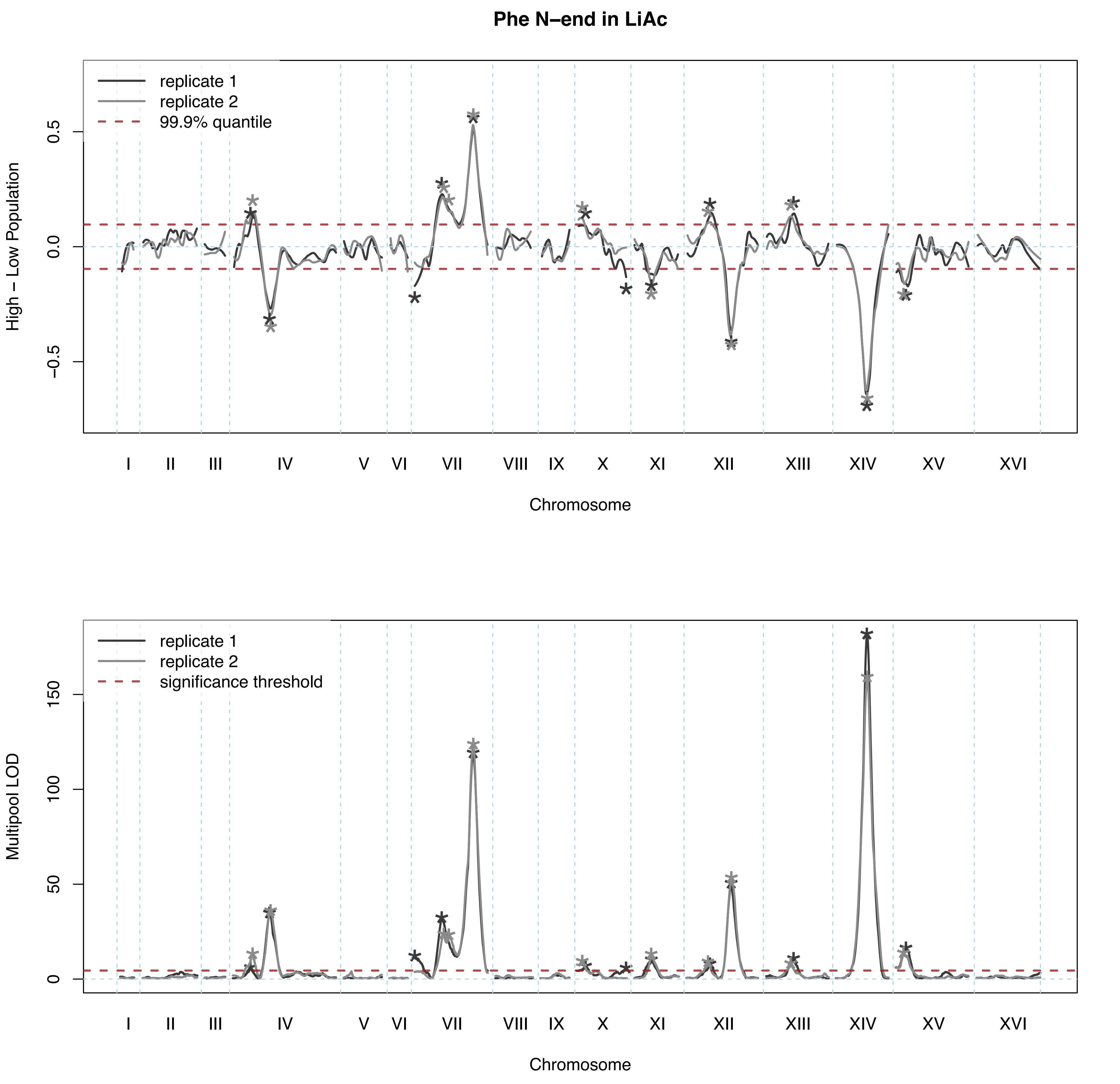

Phe N–end in LiAc
replicate 1
replicate 2
99.9% quantile
High – Low Population
0.5
0.0
−0.5
I
II
III
IV
V
VI
VII
VIII
IX
X
XI
XII
XIII
XIV
XV
XVI
Chromosome
replicate 1
replicate 2
significance threshold
Multipool LOD
0
50
100
150
I
II
III
IV
V
VI
VII
VIII
IX
X
XI
XII
XIII
XIV
XV
XVI
Chromosome

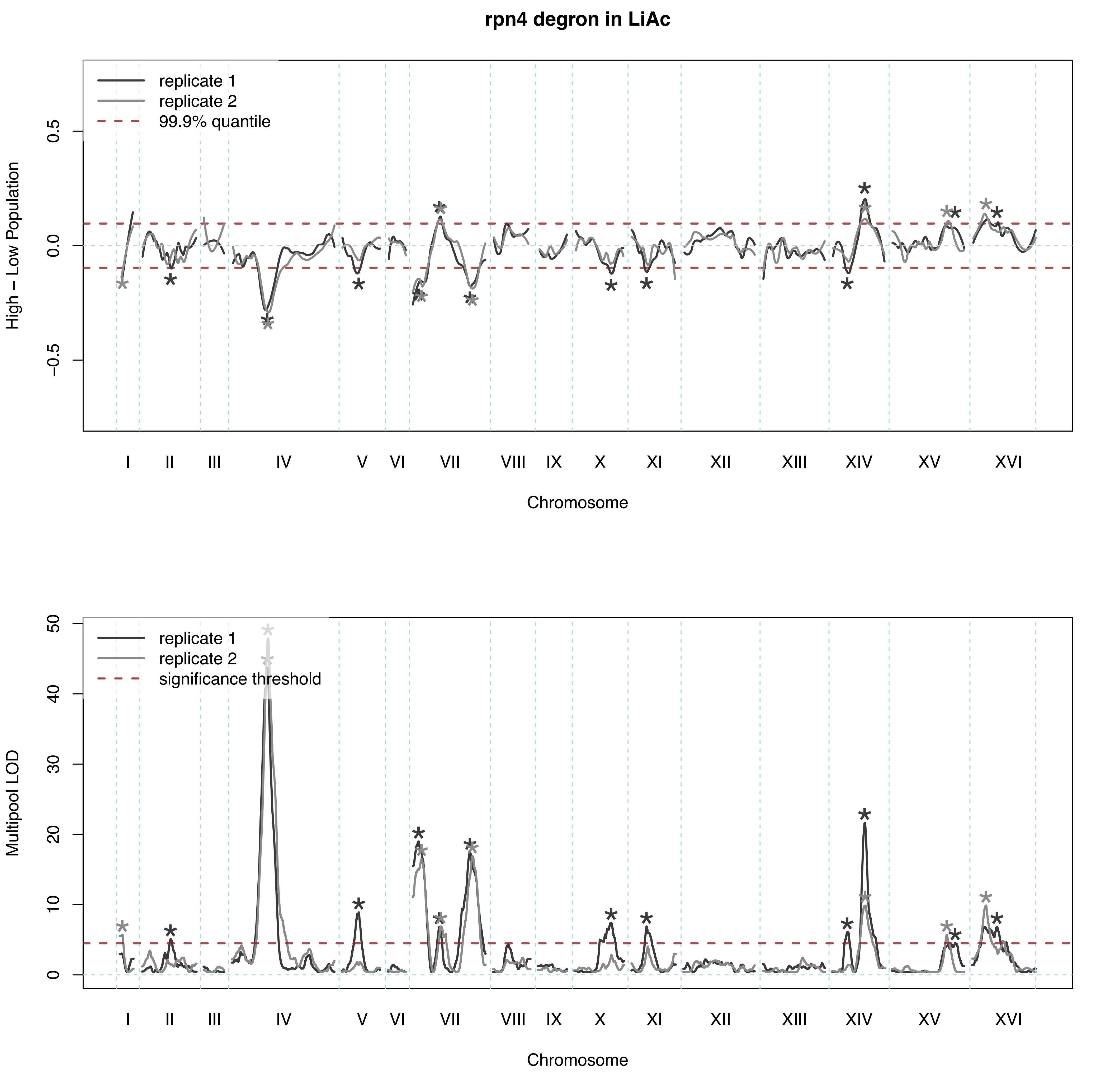
rpn4 degron in LiAc
replicate 1
replicate 2
99.9% quantile
High – Low Population
0.5
0.0
−0.5
I
II
III
IV
V
VI
VII
VIII
IX
X
XI
XII
XIII
XIV
XV
XVI
Chromosome
replicate 1
replicate 2
significance threshold
Multipool LOD
50
40
30
20
10
0
I
II
III
IV
V
VI
VII
VIII
IX
X
XI
XII
XIII
XIV
XV
XVI
Chromosome

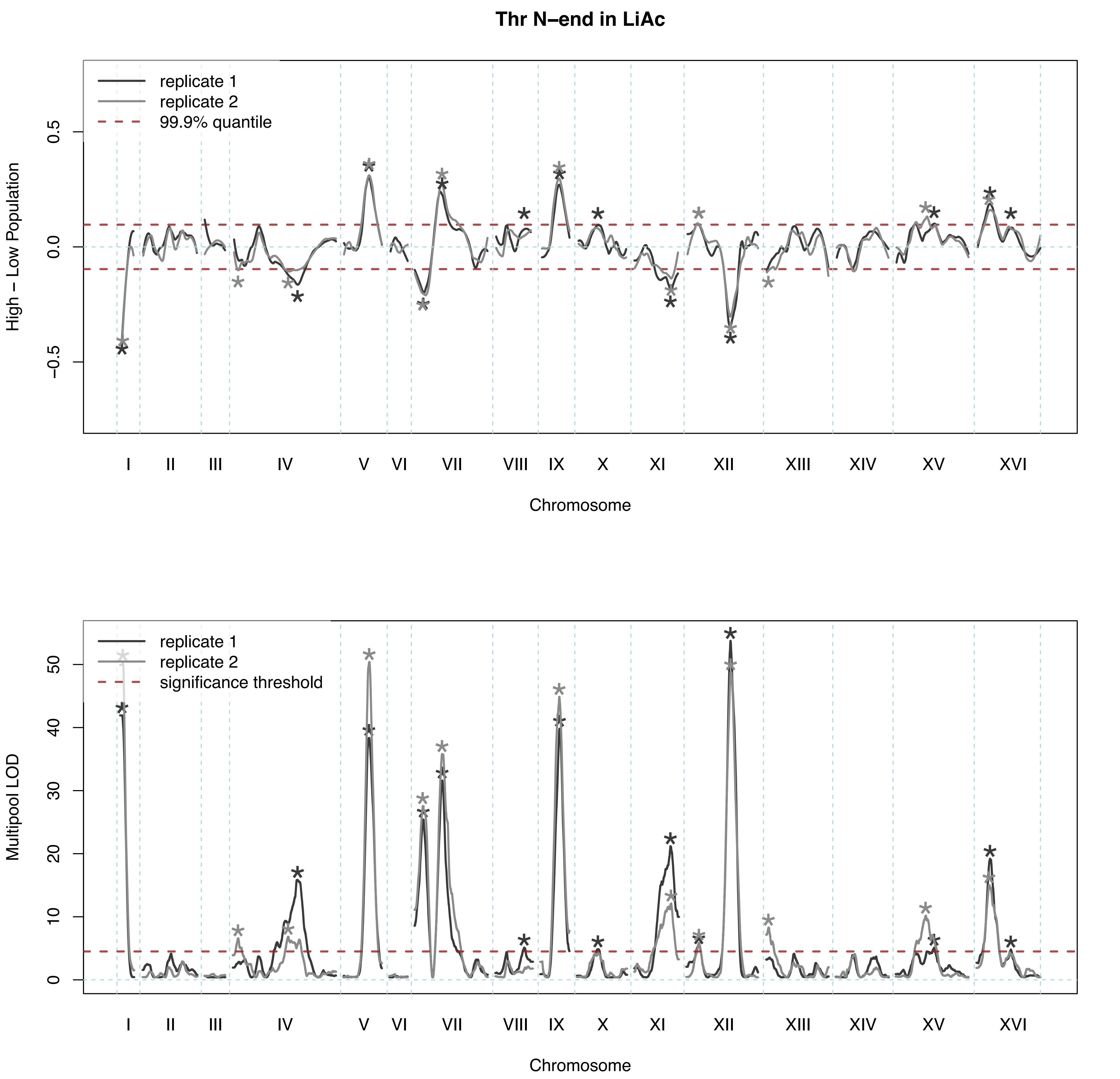

Thr N–end in LiAc
replicate 1
replicate 2
99.9% quantile
High – Low Population
0.5
0.0
−0.5
I II III IV V VI VII VIII IX X XI XII XIII XIV XV XVI
Chromosome
replicate 1
replicate 2
significance threshold
Multipool LOD
0
10
20
30
40
50
I II III IV V VI VII VIII IX X XI XII XIII XIV XV XVI
Chromosome

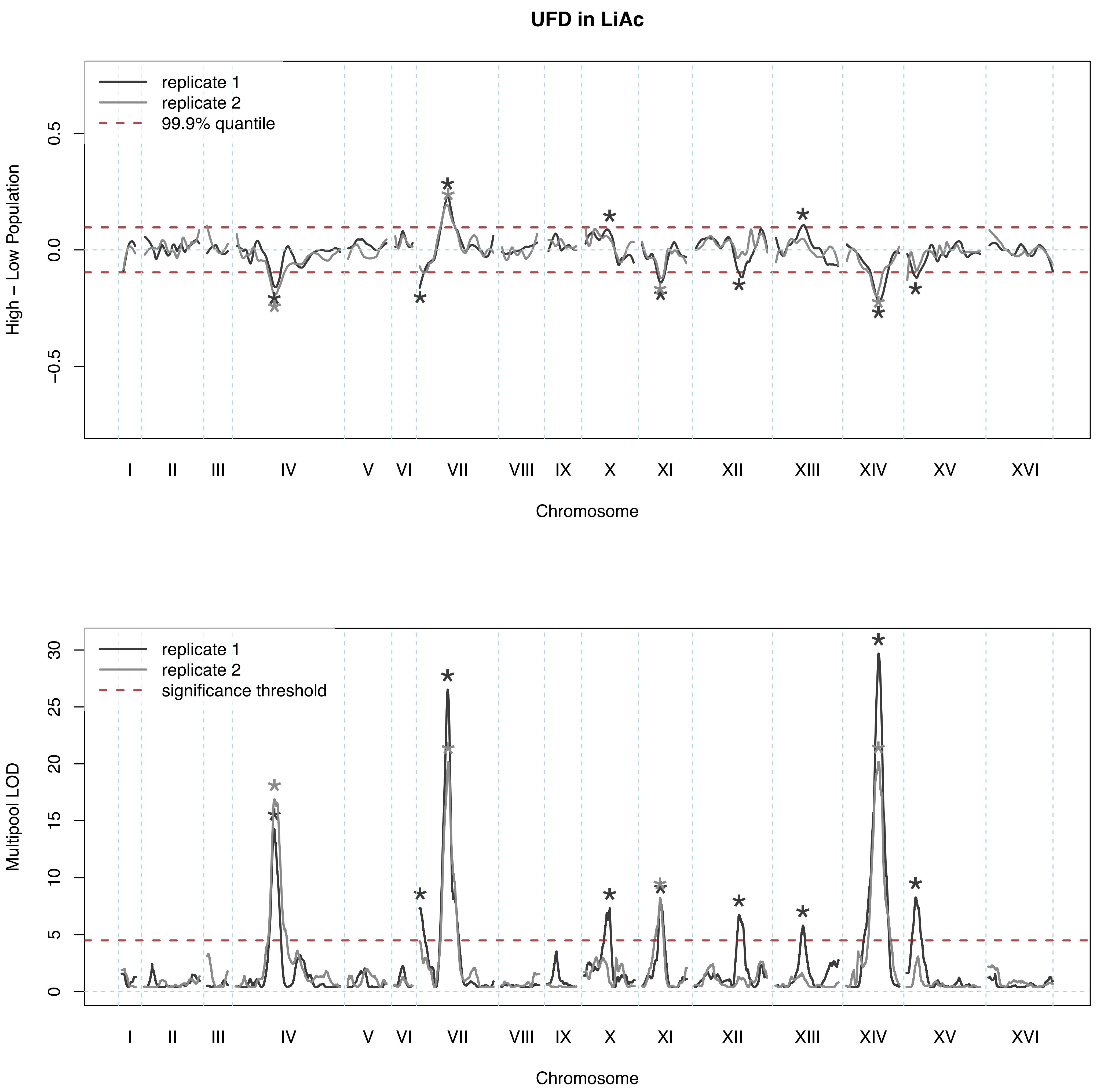
UFD in LiAc
replicate 1
replicate 2
99.9% quantile
High – Low Population
0.5
0.0
−0.5
I II III IV V VI VII VIII IX X XI XII XIII XIV XV XVI
Chromosome
replicate 1
replicate 2
significance threshold
Multipool LOD
30
25
20
15
10
5
0
I II III IV V VI VII VIII IX X XI XII XIII XIV XV XVI
Chromosome

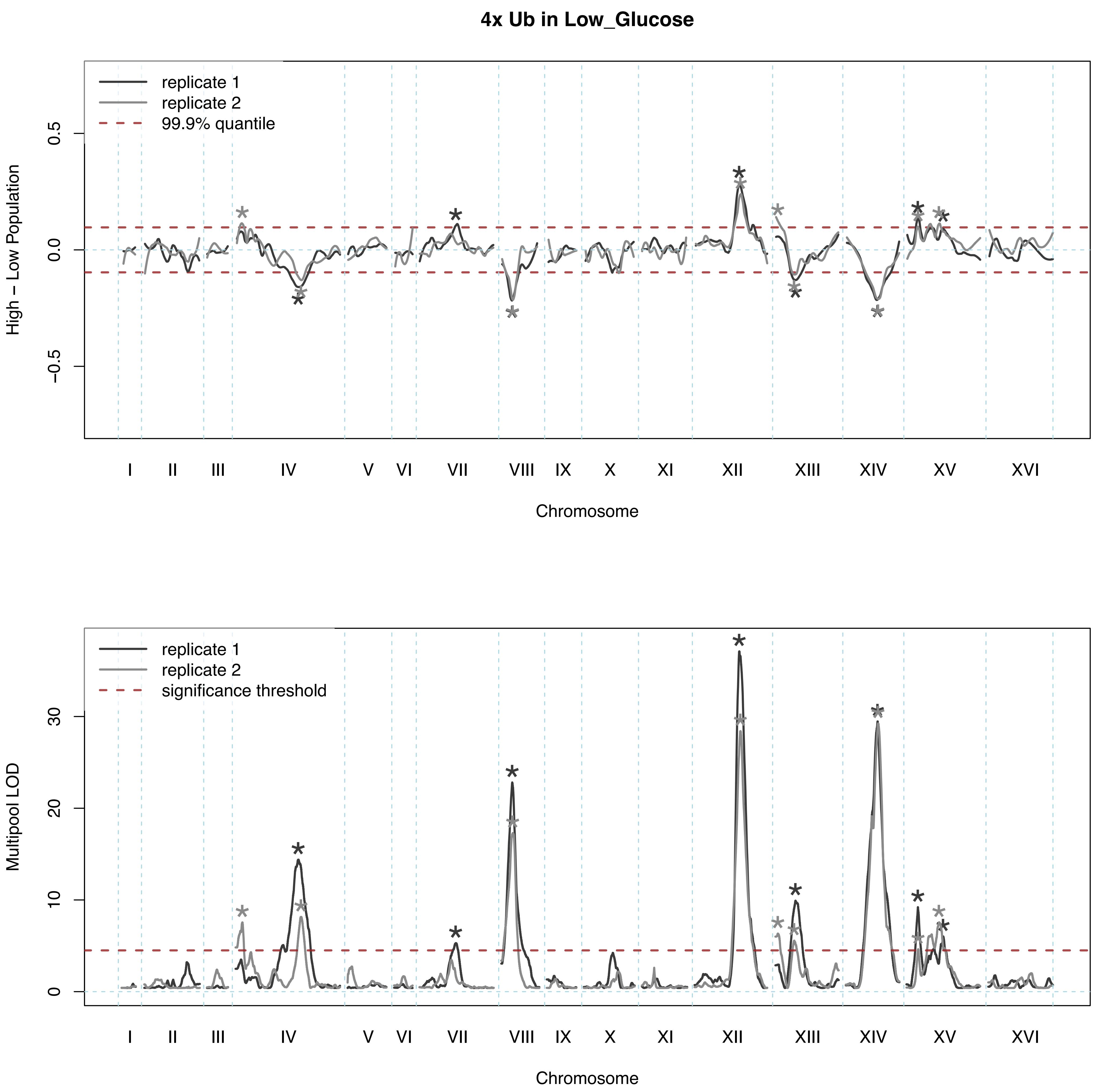
4x Ub in Low_Glucose
replicate 1
replicate 2
99.9% quantile
0.5
0.0
−0.5
High − Low Population
I
II
III
IV
V
VI
VII
VIII
IX
X
XI
XII
XIII
XIV
XV
XVI
Chromosome
replicate 1
replicate 2
significance threshold
0
10
20
30
Multipool LOD
I
II
III
IV
V
VI
VII
VIII
IX
X
XI
XII
XIII
XIV
XV
XVI
Chromosome

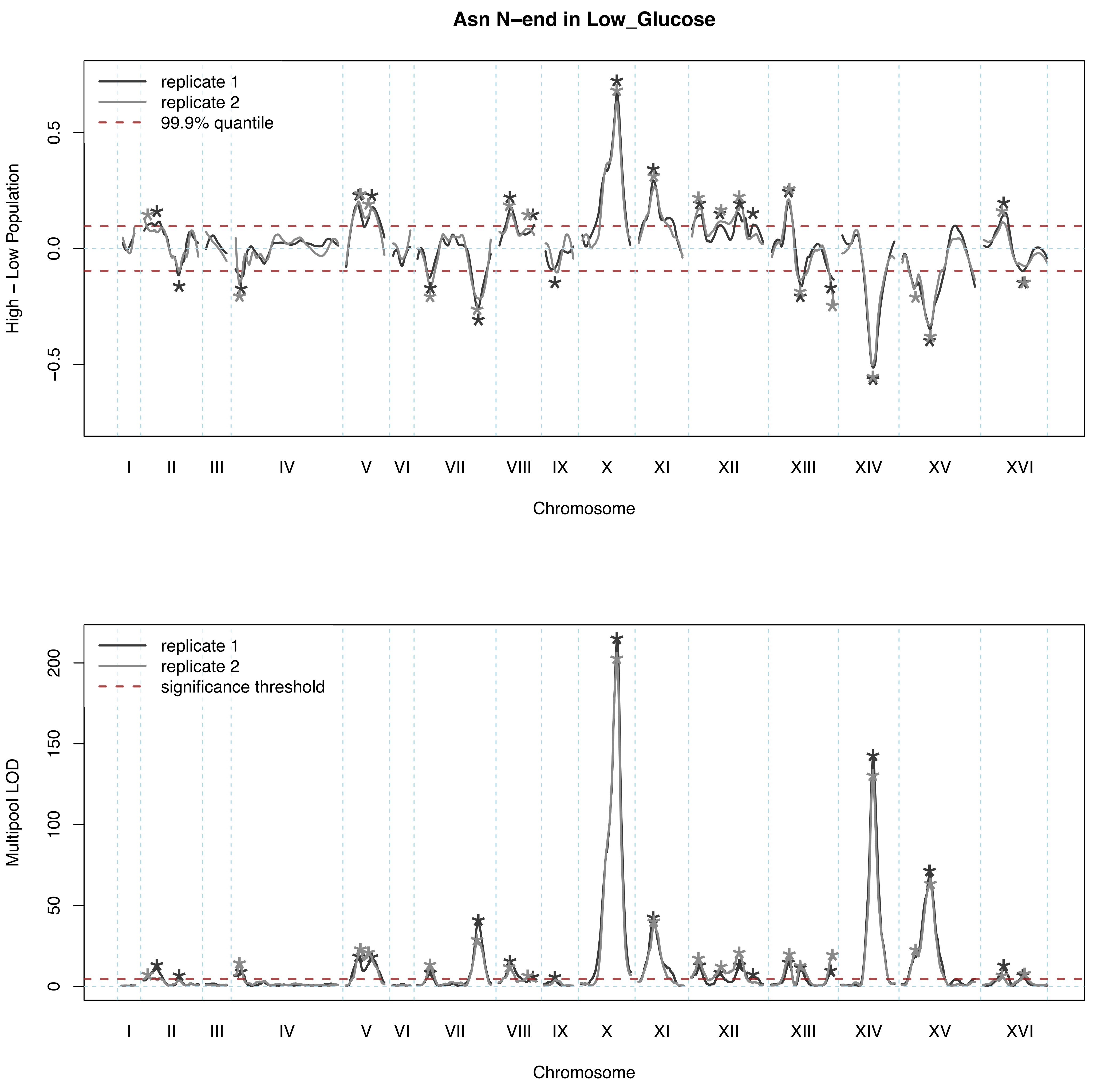
Asn N–end in Low_Glucose
replicate 1
replicate 2
99.9% quantile
High – Low Population
0.5
0.0
−0.5
I
II
III
IV
V
VI
VII
VIII
IX
X
XI
XII
XIII
XIV
XV
XVI
Chromosome
replicate 1
replicate 2
significance threshold
Multipool LOD
200
150
100
50
0
I
II
III
IV
V
VI
VII
VIII
IX
X
XI
XII
XIII
XIV
XV
XVI
Chromosome

# Phe N–end in Low_Glucose

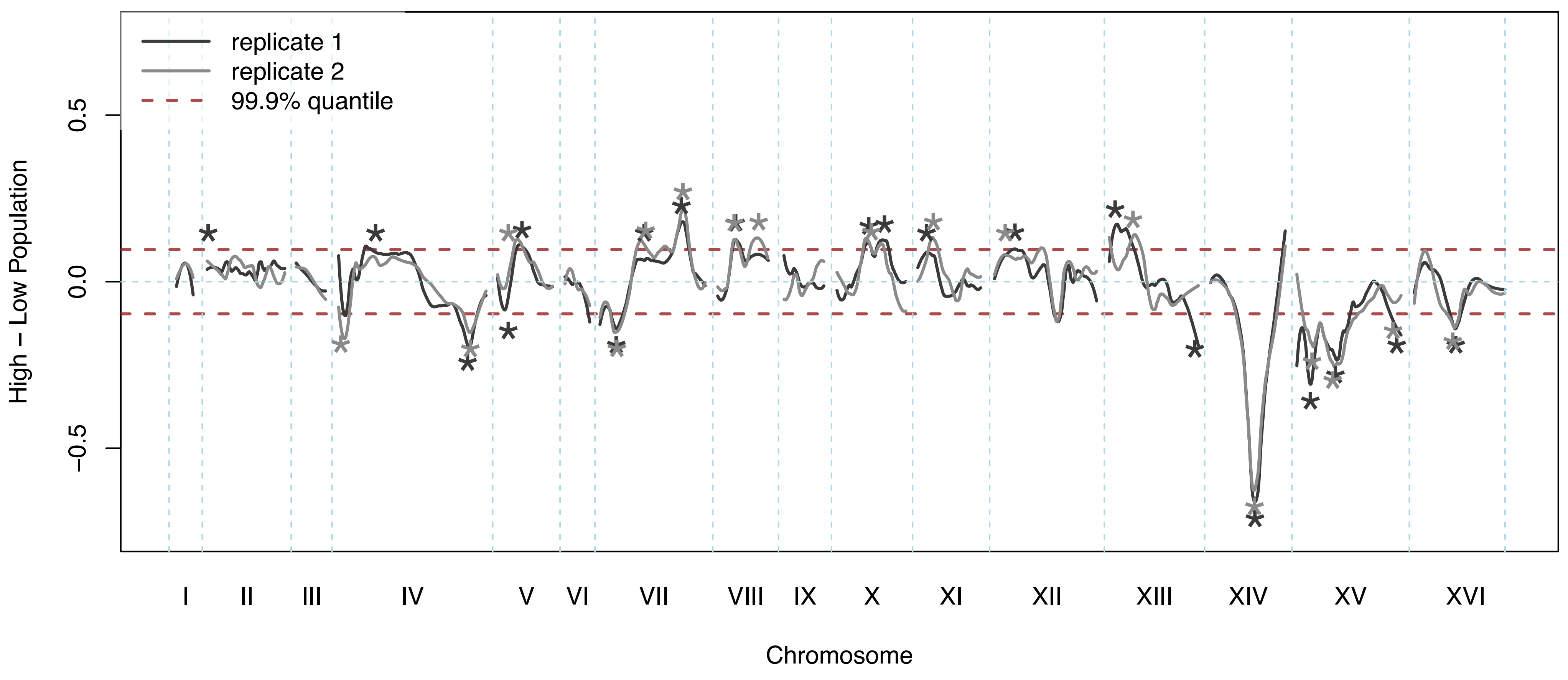


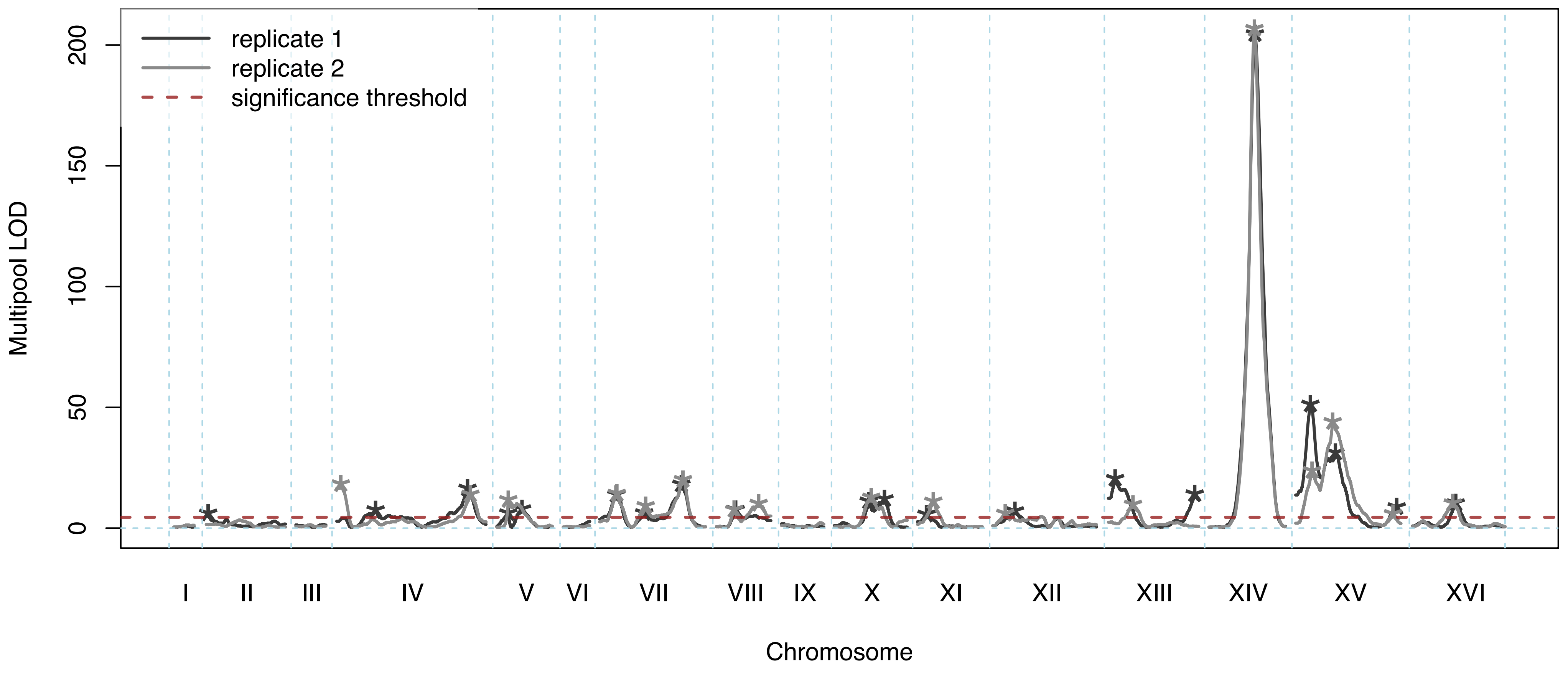

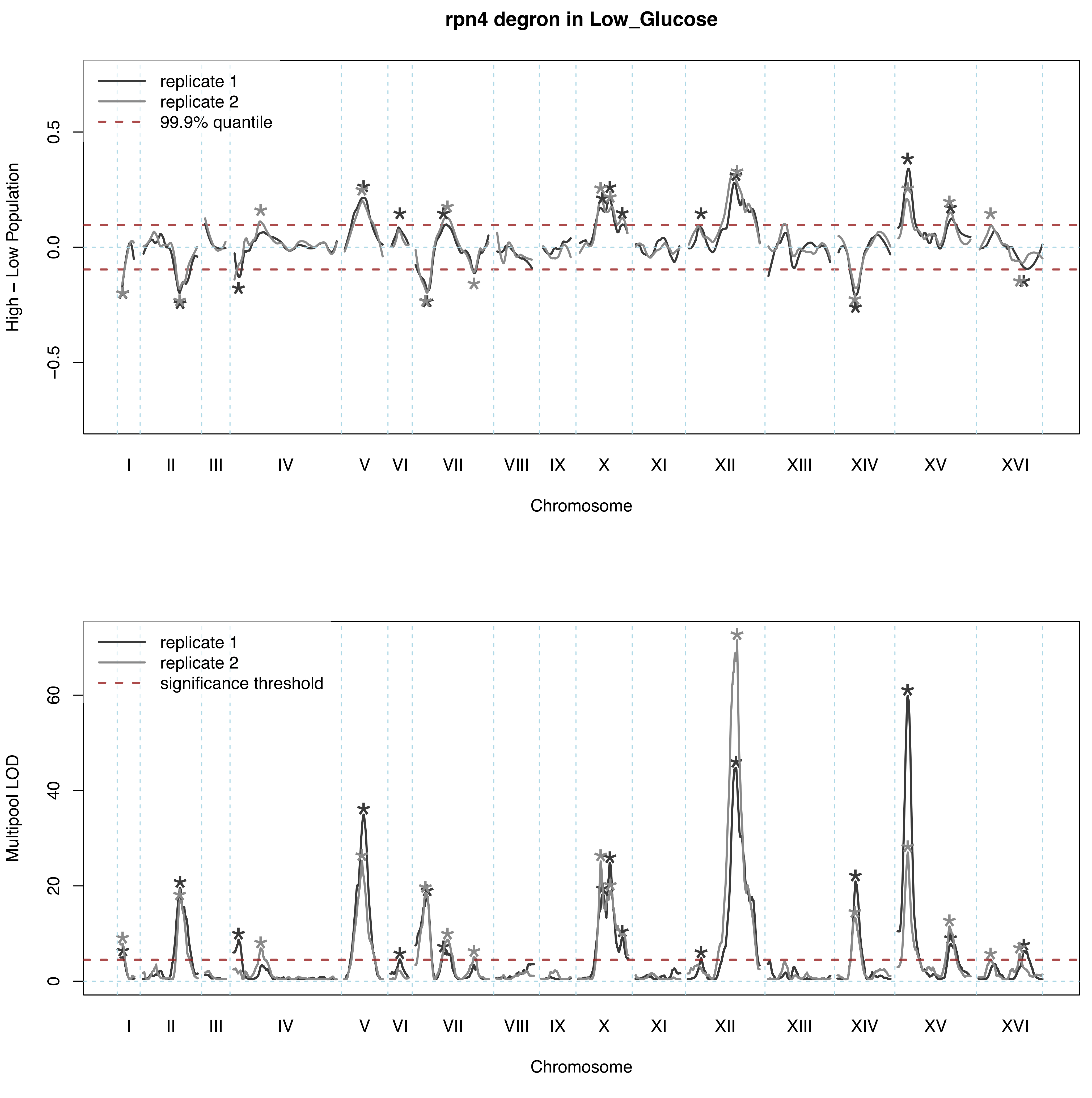
rpn4 degron in Low_Glucose
replicate 1
replicate 2
99.9% quantile
High − Low Population
0.5
0.0
−0.5
I
II
III
IV
V
VI
VII
VIII
IX
X
XI
XII
XIII
XIV
XV
XVI
Chromosome
replicate 1
replicate 2
significance threshold
Multipool LOD
0
20
40
60
I
II
III
IV
V
VI
VII
VIII
IX
X
XI
XII
XIII
XIV
XV
XVI
Chromosome

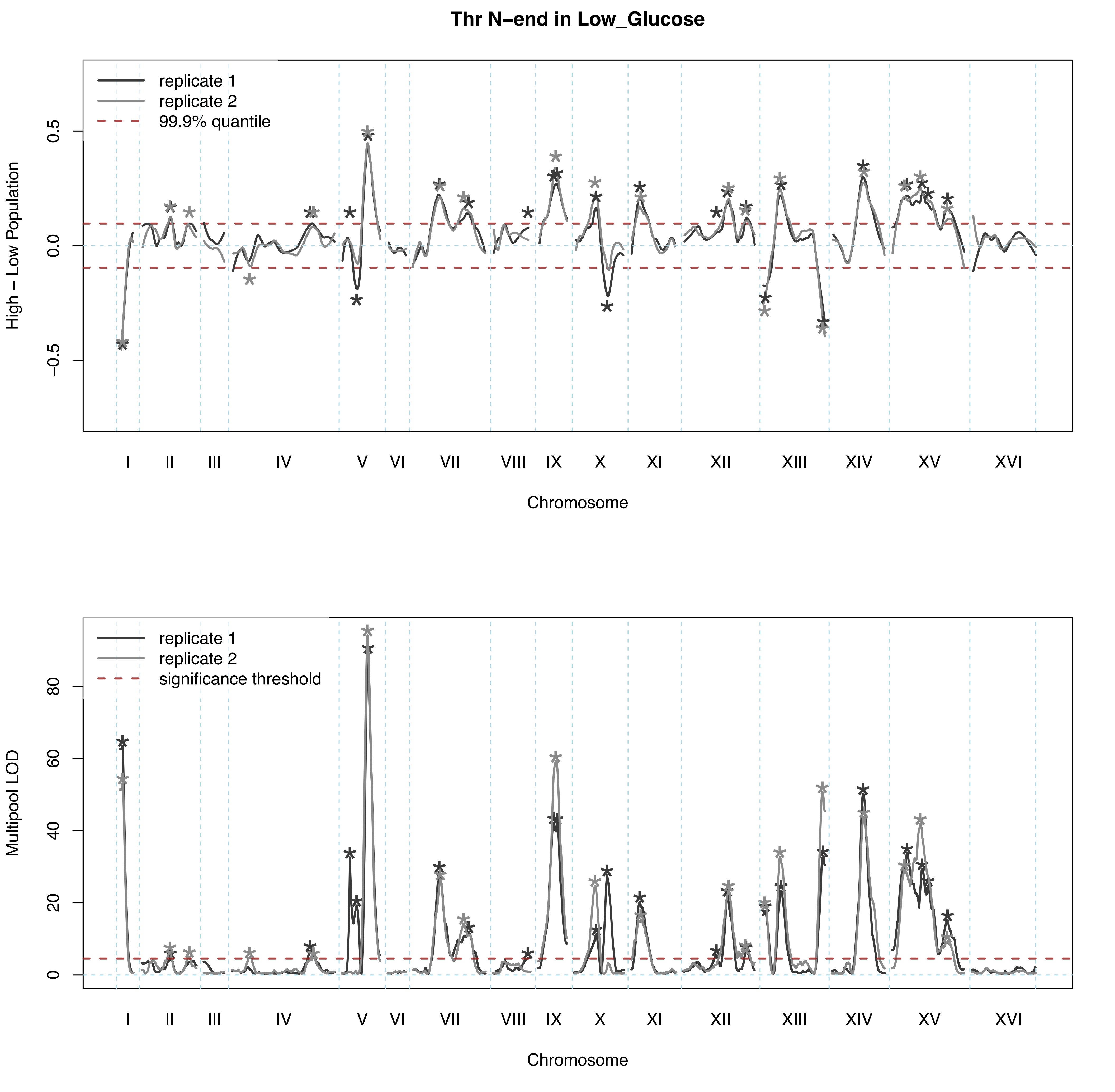

Thr N–end in Low_Glucose
replicate 1
replicate 2
99.9% quantile
High – Low Population
0.5
0.0
−0.5
I
II
III
IV
V
VI
VII
VIII
IX
X
XI
XII
XIII
XIV
XV
XVI
Chromosome
replicate 1
replicate 2
significance threshold
Multipool LOD
80
60
40
20
0
I
II
III
IV
V
VI
VII
VIII
IX
X
XI
XII
XIII
XIV
XV
XVI
Chromosome

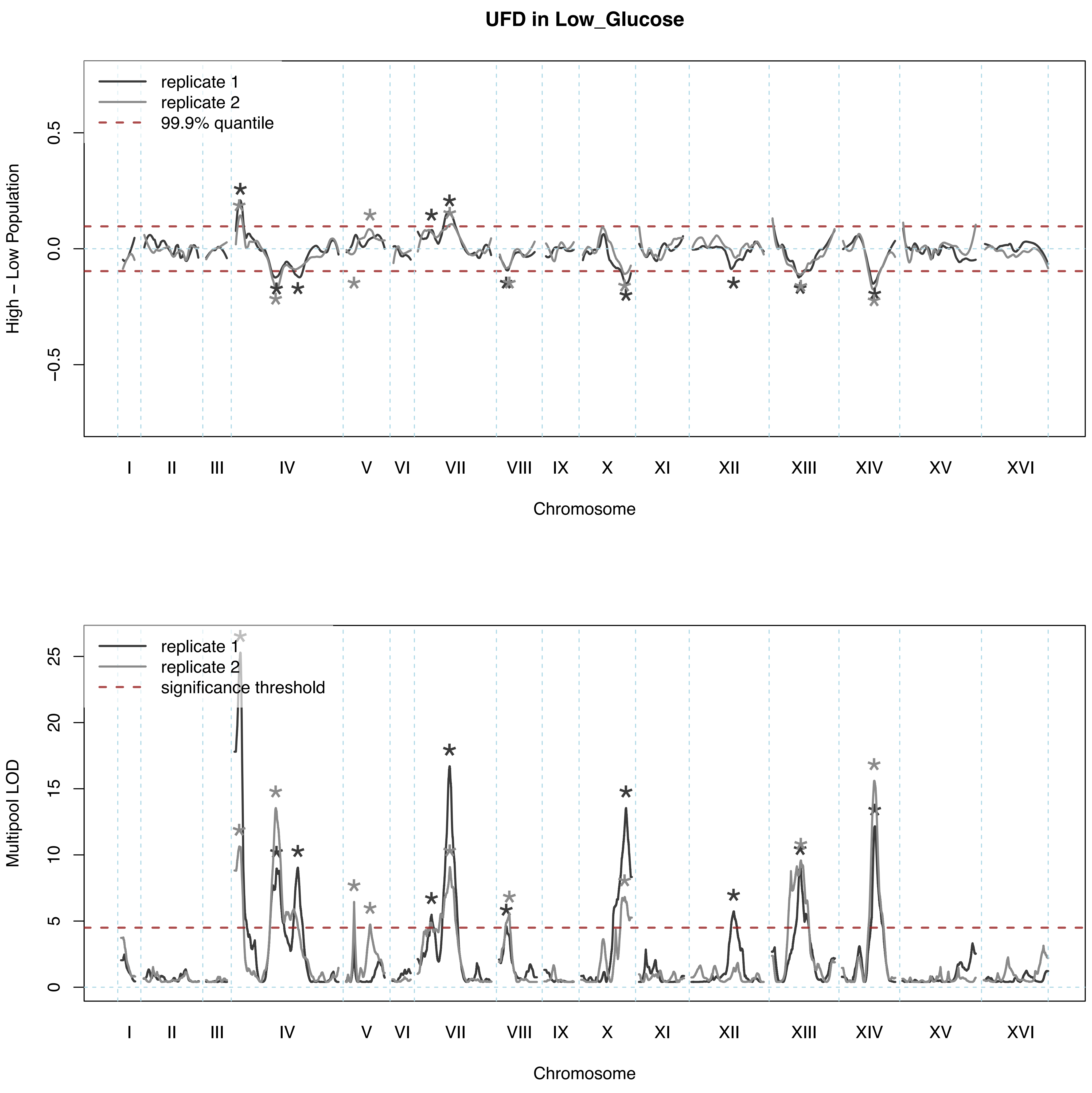
UFD in Low_Glucose
replicate 1
replicate 2
99.9% quantile
High – Low Population
0.5
0.0
−0.5
I
II
III
IV
V
VI
VII
VIII
IX
X
XI
XII
XIII
XIV
XV
XVI
Chromosome
replicate 1
replicate 2
significance threshold
Multipool LOD
25
20
15
10
5
0
I
II
III
IV
V
VI
VII
VIII
IX
X
XI
XII
XIII
XIV
XV
XVI
Chromosome

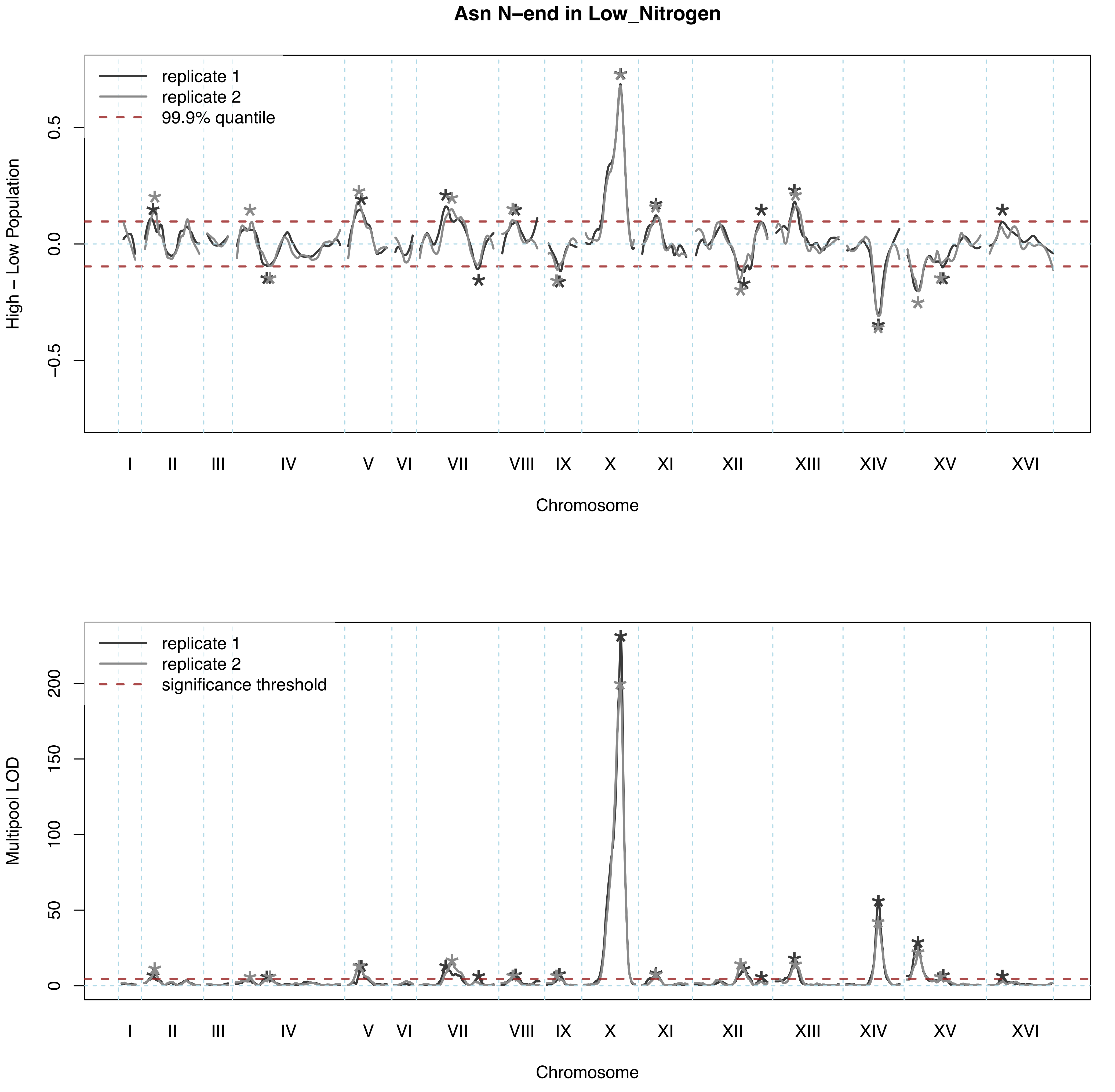

Asn N–end in Low_Nitrogen
replicate 1
replicate 2
99.9% quantile
High – Low Population
0.5
0.0
−0.5
I
II
III
IV
V
VI
VII
VIII
IX
X
XI
XII
XIII
XIV
XV
XVI
Chromosome
replicate 1
replicate 2
significance threshold
Multipool LOD
200
150
100
50
0
I
II
III
IV
V
VI
VII
VIII
IX
X
XI
XII
XIII
XIV
XV
XVI
Chromosome

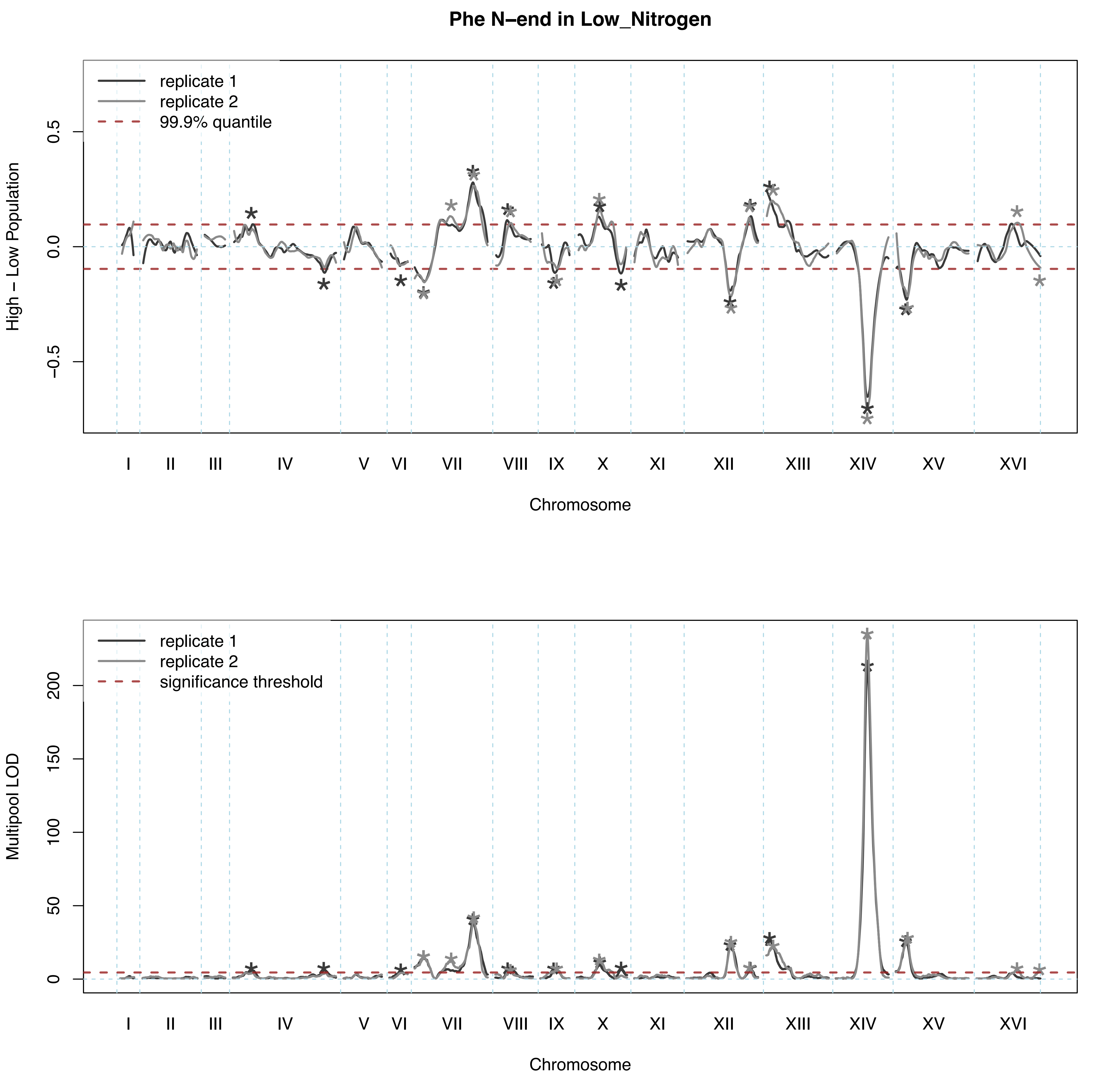
Phe N–end in Low_Nitrogen
replicate 1
replicate 2
99.9% quantile
High – Low Population
0.5
0.0
−0.5
I
II
III
IV
V
VI
VII
VIII
IX
X
XI
XII
XIII
XIV
XV
XVI
Chromosome
replicate 1
replicate 2
significance threshold
Multipool LOD
200
150
100
50
0
Chromosome

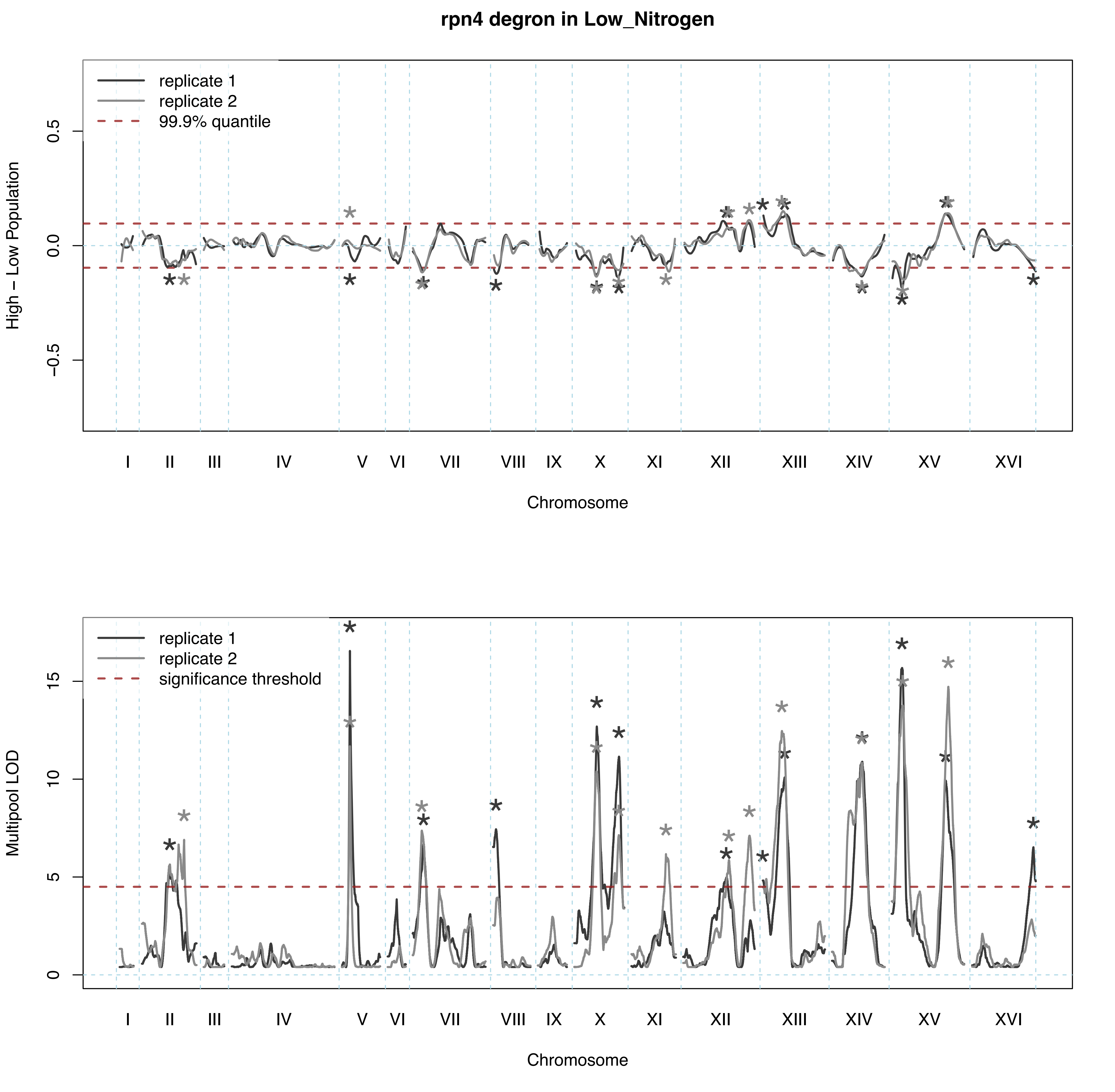

rpn4 degron in Low_Nitrogen
replicate 1
replicate 2
99.9% quantile
High – Low Population
0.5
0.0
−0.5
I
II
III
IV
V
VI
VII
VIII
IX
X
XI
XII
XIII
XIV
XV
XVI
Chromosome
replicate 1
replicate 2
significance threshold
Multipool LOD
0
5
10
15
Chromosome

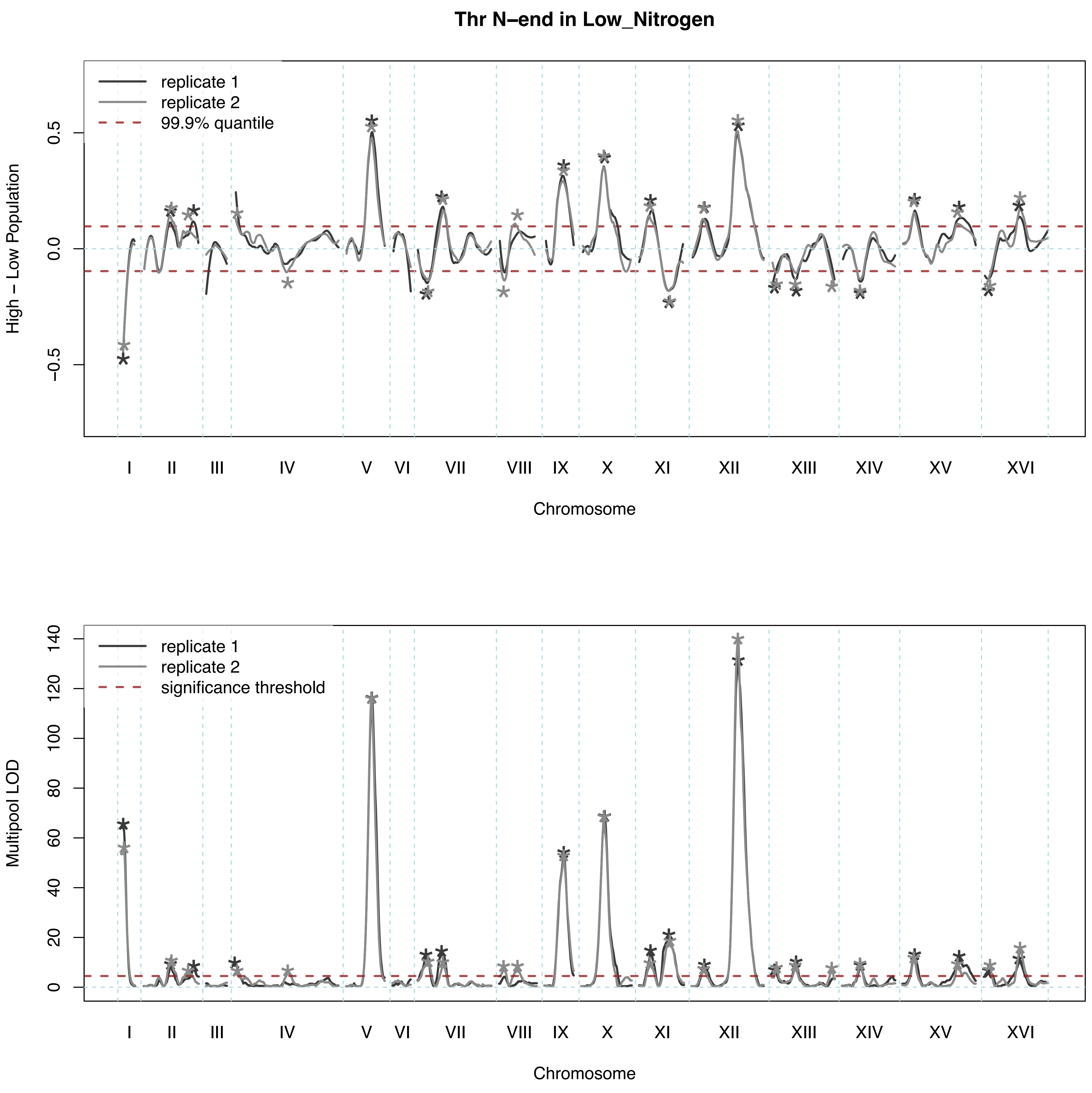

Thr N–end in Low_Nitrogen
replicate 1
replicate 2
99.9% quantile
High – Low Population
0.5
0.0
−0.5
I
II
III
IV
V
VI
VII
VIII
IX
X
XI
XII
XIII
XIV
XV
XVI
Chromosome
replicate 1
replicate 2
significance threshold
Multipool LOD
140
120
100
80
60
40
20
0
I
II
III
IV
V
VI
VII
VIII
IX
X
XI
XII
XIII
XIV
XV
XVI
Chromosome

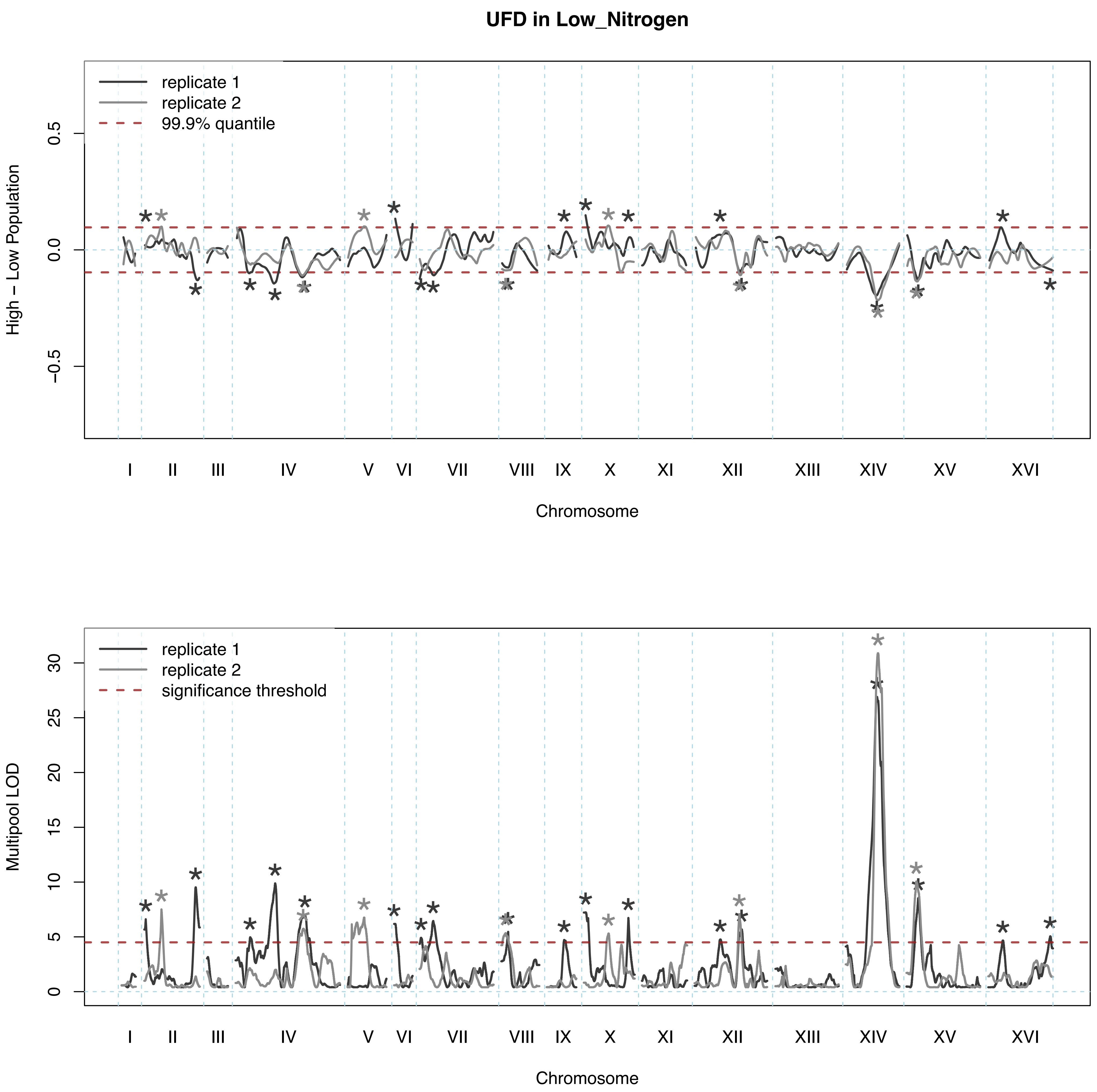

UFD in Low_Nitrogen
replicate 1
replicate 2
99.9% quantile
High – Low Population
0.5
0.0
−0.5
I II III IV V VI VII VIII IX X XI XII XIII XIV XV XVI
Chromosome
replicate 1
replicate 2
significance threshold
Multipool LOD
0
5
10
15
20
25
30
I II III IV V VI VII VIII IX X XI XII XIII XIV XV XVI
Chromosome

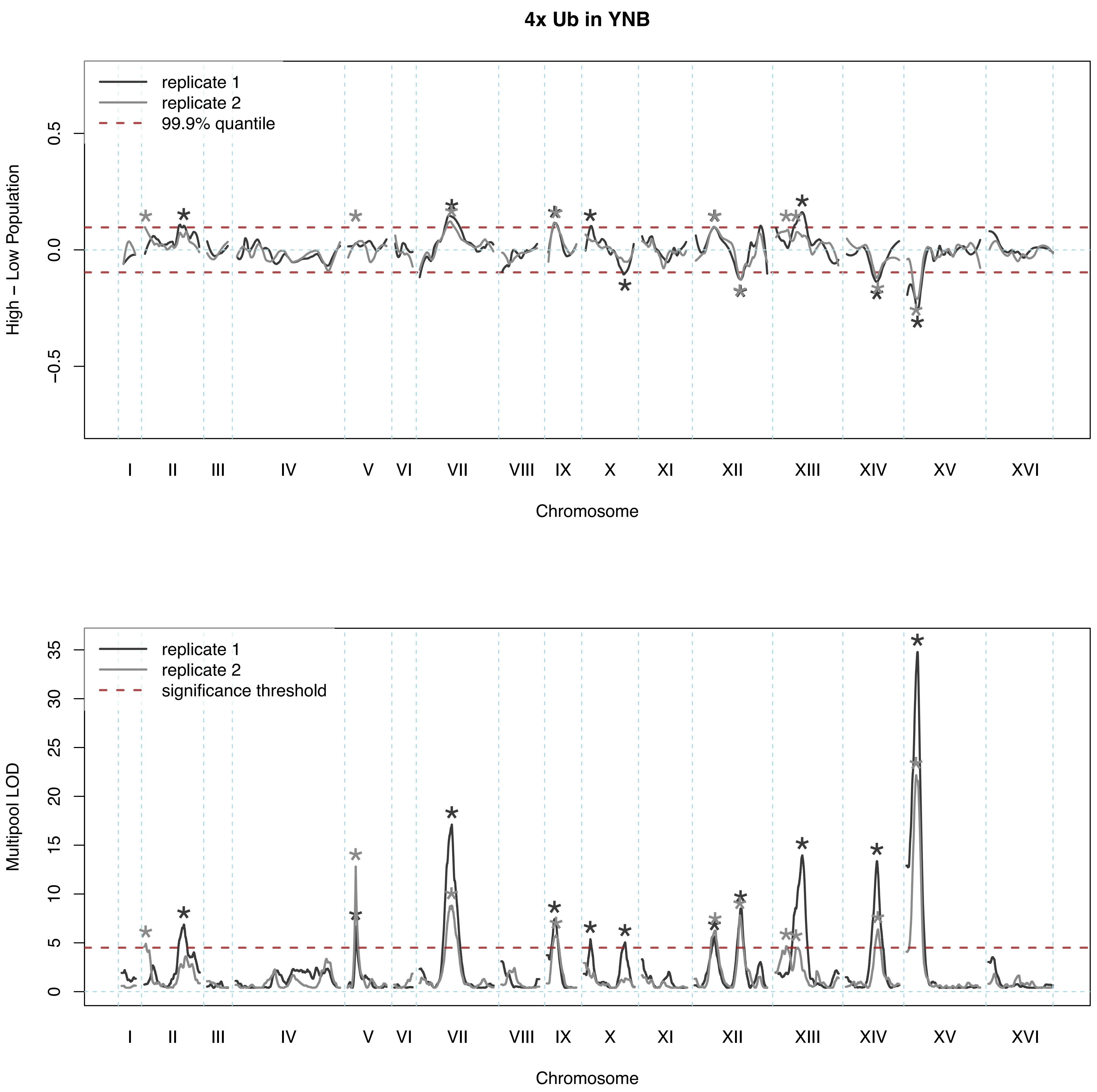
4x Ub in YNB
replicate 1
replicate 2
99.9% quantile
High – Low Population
0.5
0.0
−0.5
I
II
III
IV
V
VI
VII
VIII
IX
X
XI
XII
XIII
XIV
XV
XVI
Chromosome
replicate 1
replicate 2
significance threshold
Multipool LOD
35
30
25
20
15
10
5
0
I
II
III
IV
V
VI
VII
VIII
IX
X
XI
XII
XIII
XIV
XV
XVI
Chromosome

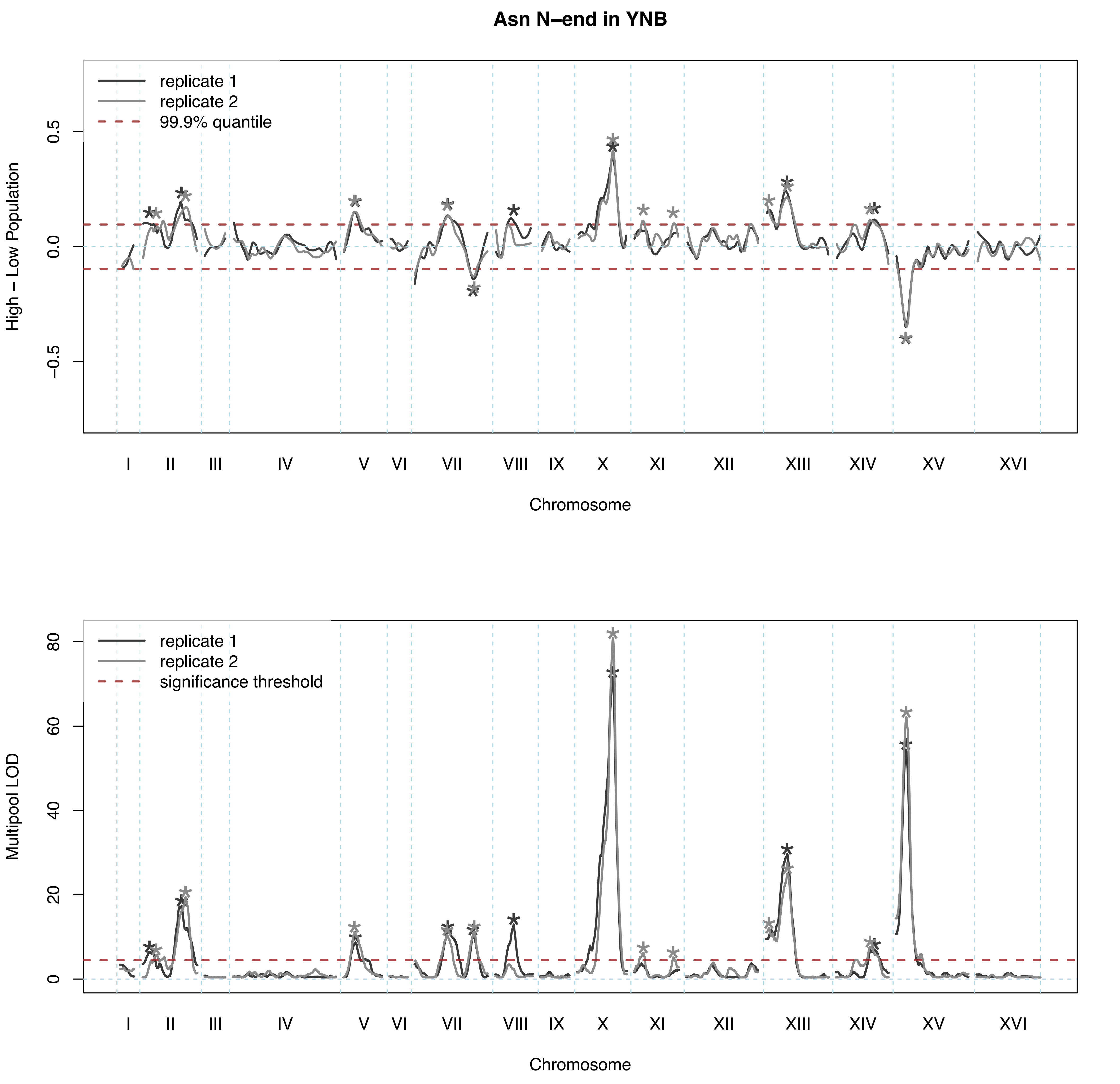
Asn N–end in YNB
replicate 1
replicate 2
99.9% quantile
High – Low Population
0.5
0.0
−0.5
I
II
III
IV
V
VI
VII
VIII
IX
X
XI
XII
XIII
XIV
XV
XVI
Chromosome
replicate 1
replicate 2
significance threshold
Multipool LOD
80
60
40
20
0
I
II
III
IV
V
VI
VII
VIII
IX
X
XI
XII
XIII
XIV
XV
XVI
Chromosome

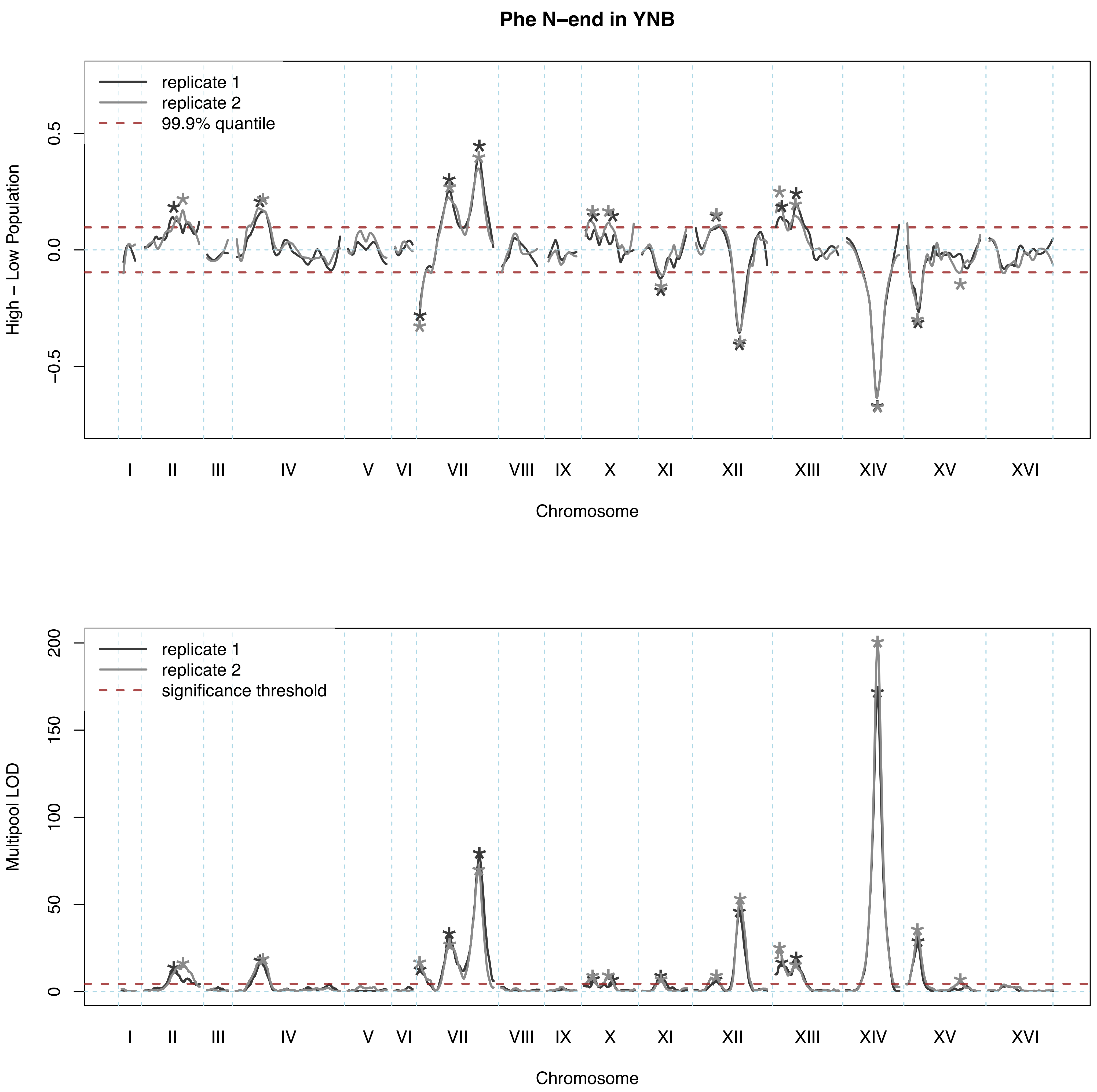

Phe N–end in YNB
replicate 1
replicate 2
99.9% quantile
High – Low Population
0.5
0.0
−0.5
I
II
III
IV
V
VI
VII
VIII
IX
X
XI
XII
XIII
XIV
XV
XVI
Chromosome
replicate 1
replicate 2
significance threshold
Multipool LOD
200
150
100
50
0
Chromosome

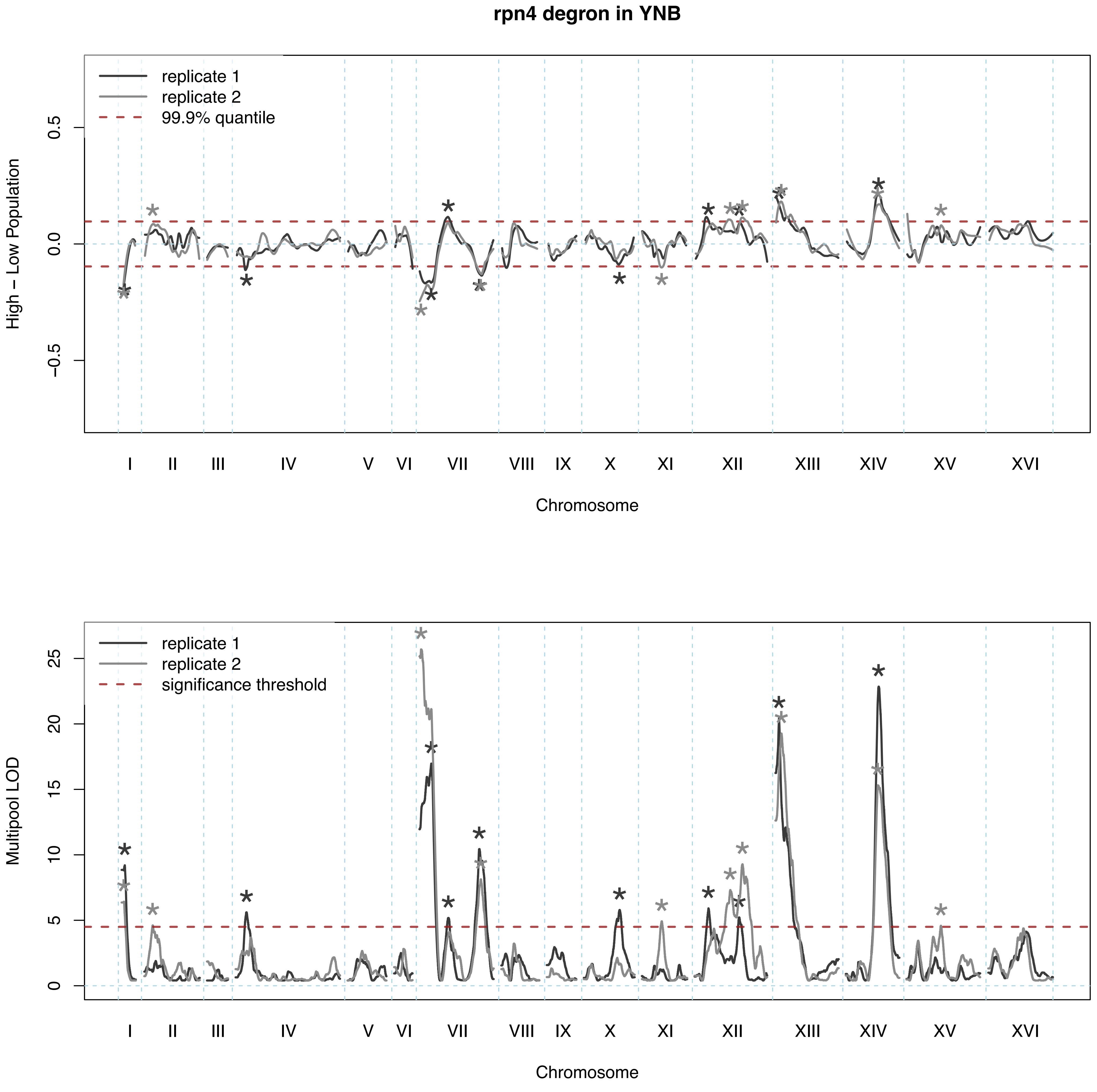

rpn4 degron in YNB
replicate 1
replicate 2
99.9% quantile
High – Low Population
0.5
0.0
−0.5
I
II
III
IV
V
VI
VII
VIII
IX
X
XI
XII
XIII
XIV
XV
XVI
Chromosome
replicate 1
replicate 2
significance threshold
Multipool LOD
25
20
15
10
5
0
I
II
III
IV
V
VI
VII
VIII
IX
X
XI
XII
XIII
XIV
XV
XVI
Chromosome

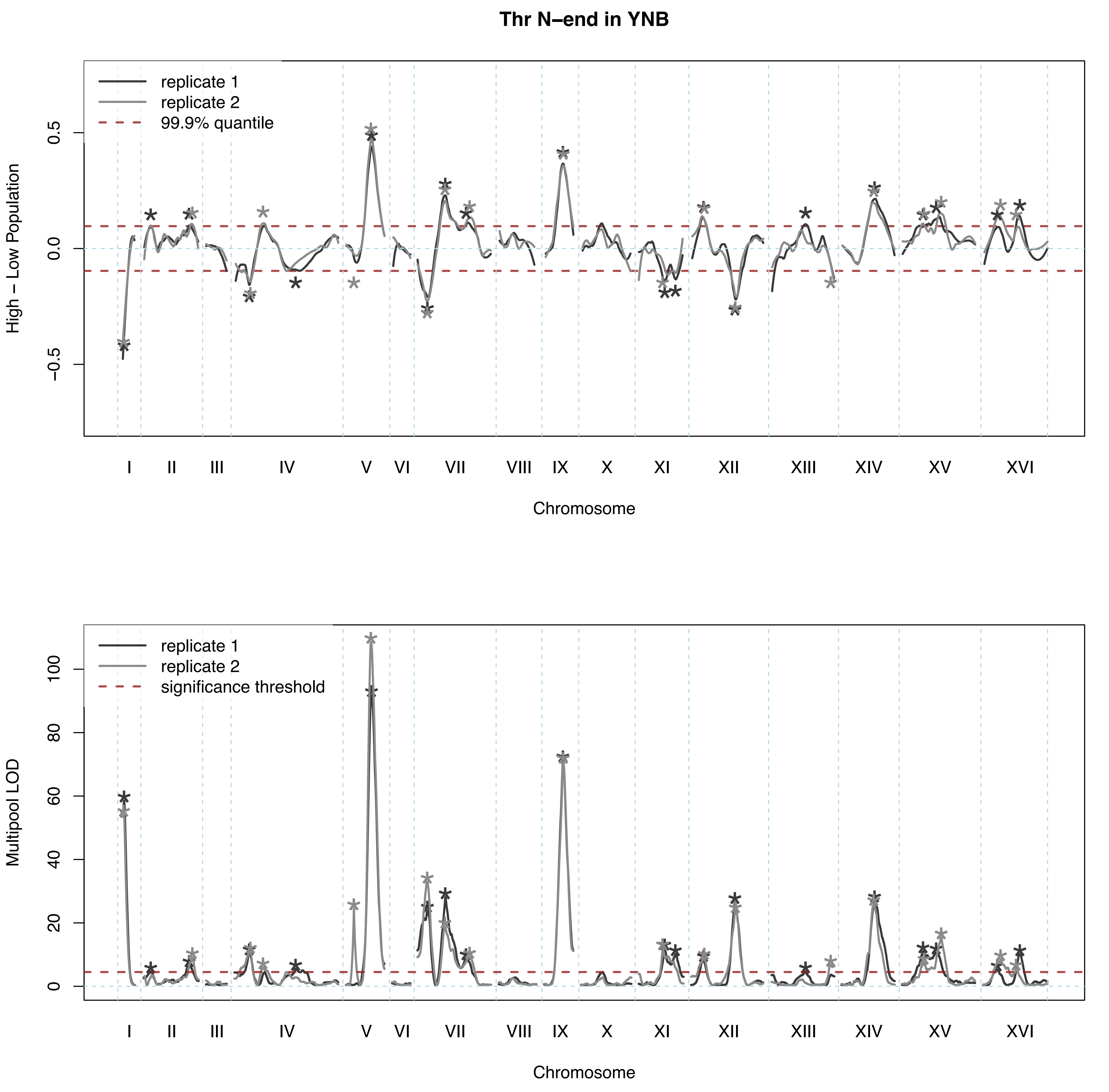
Thr N–end in YNB
replicate 1
replicate 2
99.9% quantile
High − Low Population
0.5
0.0
−0.5
I
II
III
IV
V
VI
VII
VIII
IX
X
XI
XII
XIII
XIV
XV
XVI
Chromosome
replicate 1
replicate 2
significance threshold
Multipool LOD
100
80
60
40
20
0
I
II
III
IV
V
VI
VII
VIII
IX
X
XI
XII
XIII
XIV
XV
XVI
Chromosome

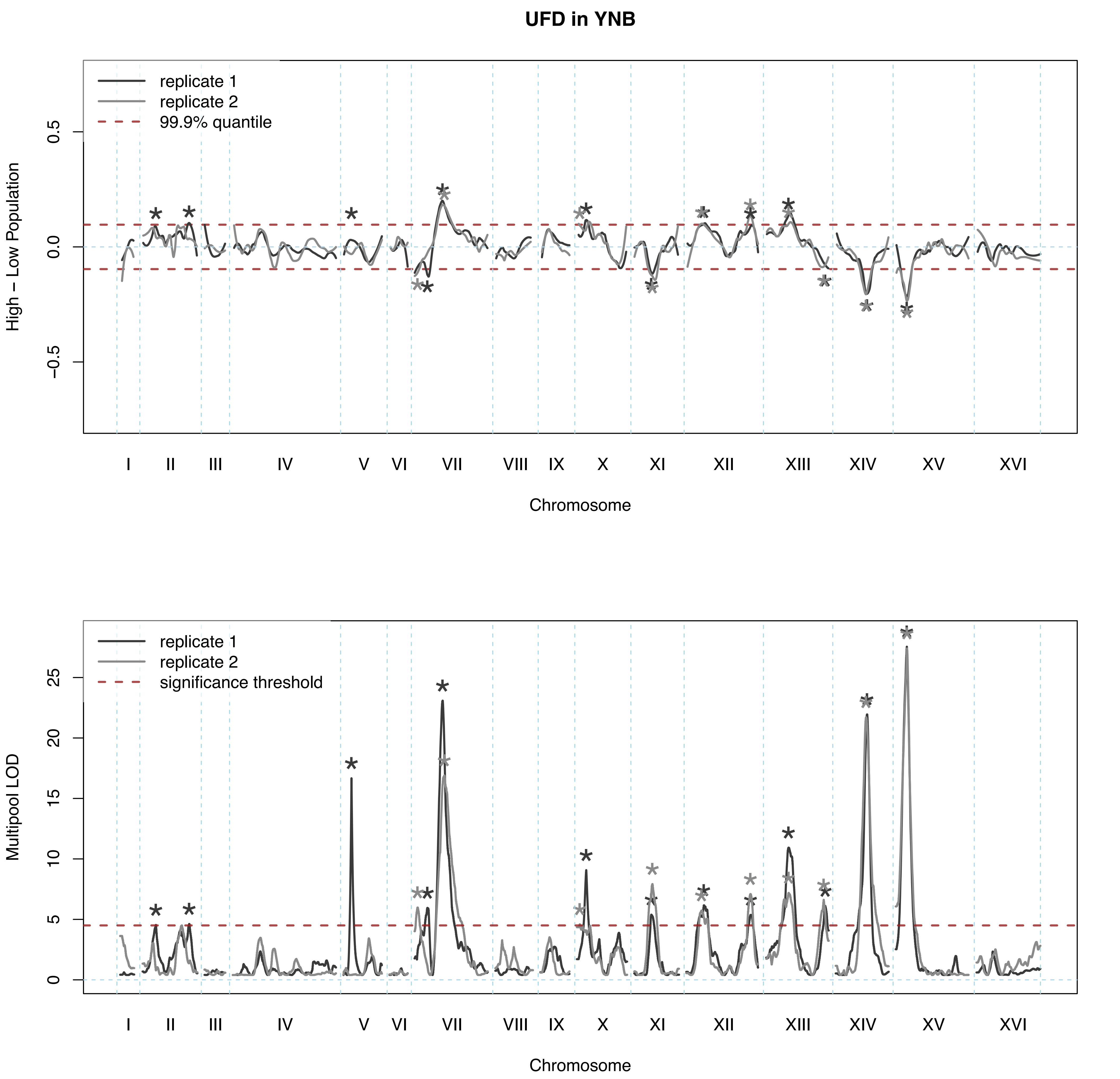

UFD in YNB
replicate 1
replicate 2
99.9% quantile
High − Low Population
0.5
0.0
−0.5
I II III IV V VI VII VIII IX X XI XII XIII XIV XV XVI
Chromosome
replicate 1
replicate 2
significance threshold
Multipool LOD
25
20
15
10
5
0
I II III IV V VI VII VIII IX X XI XII XIII XIV XV XVI
Chromosome
