# Supplementary material for: Genotype-by-environment interactions shape ubiquitin-proteasome system activity
Source: bioRxiv. 2024 Nov 21:2024.11.21.624644. Preprint. [Version 1] doi: 10.1101/2024.11.21.624644 (PMC11601593; doi:10.1101/2024.11.21.624644)
Supplement: Supplement 4 [file NIHPP2024.11.21.624644v1-supplement-4.pdf]

bioRxiv preprint doi: https://doi.org/10.1101/2024.11.21.624644; this version posted November 21, 2024. The copyright holder for this preprint (which was not certified by peer review) is the author/funder, who has granted bioRxiv a license to display the preprint in perpetuity. It is made available under aCC-BY 4.0 International license.

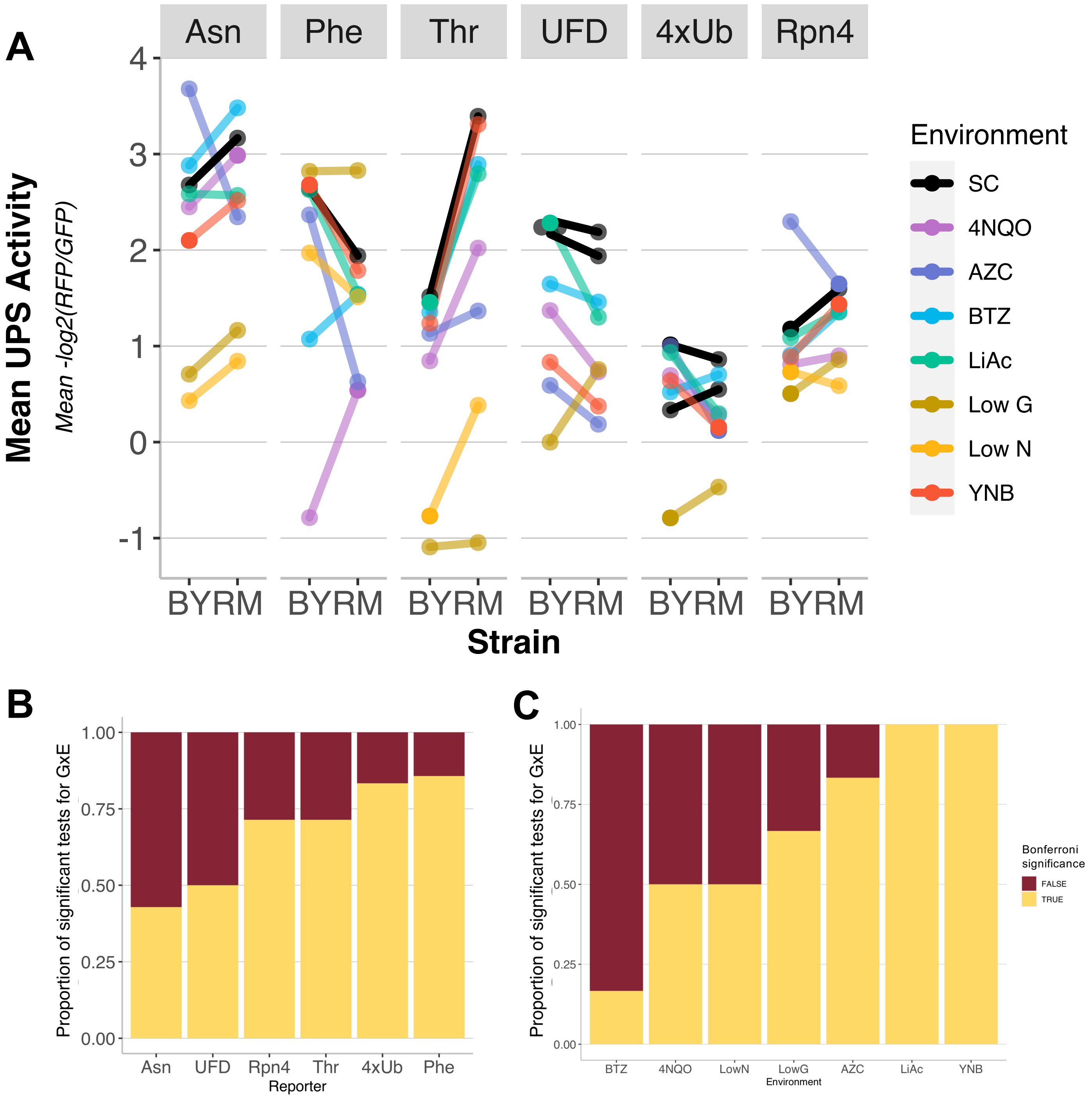

Supplementary Fig. 1: A. Mean UPS activity across eight replicates for each environment and reporter. **B.** Proportion of tests with significant (gold) and non-significant (maroon) GxE, by reporter. **C.** Data as in C, but rearranged by environment.

bioRxiv preprint doi: https://doi.org/10.1101/2024.11.21.624644; this version posted November 21, 2024. The copyright holder for this preprint (which was not certified by peer review) is the author/funder, who has granted bioRxiv a license to display the preprint in perpetuity. It is made available under aCC-BY 4.0 International license.

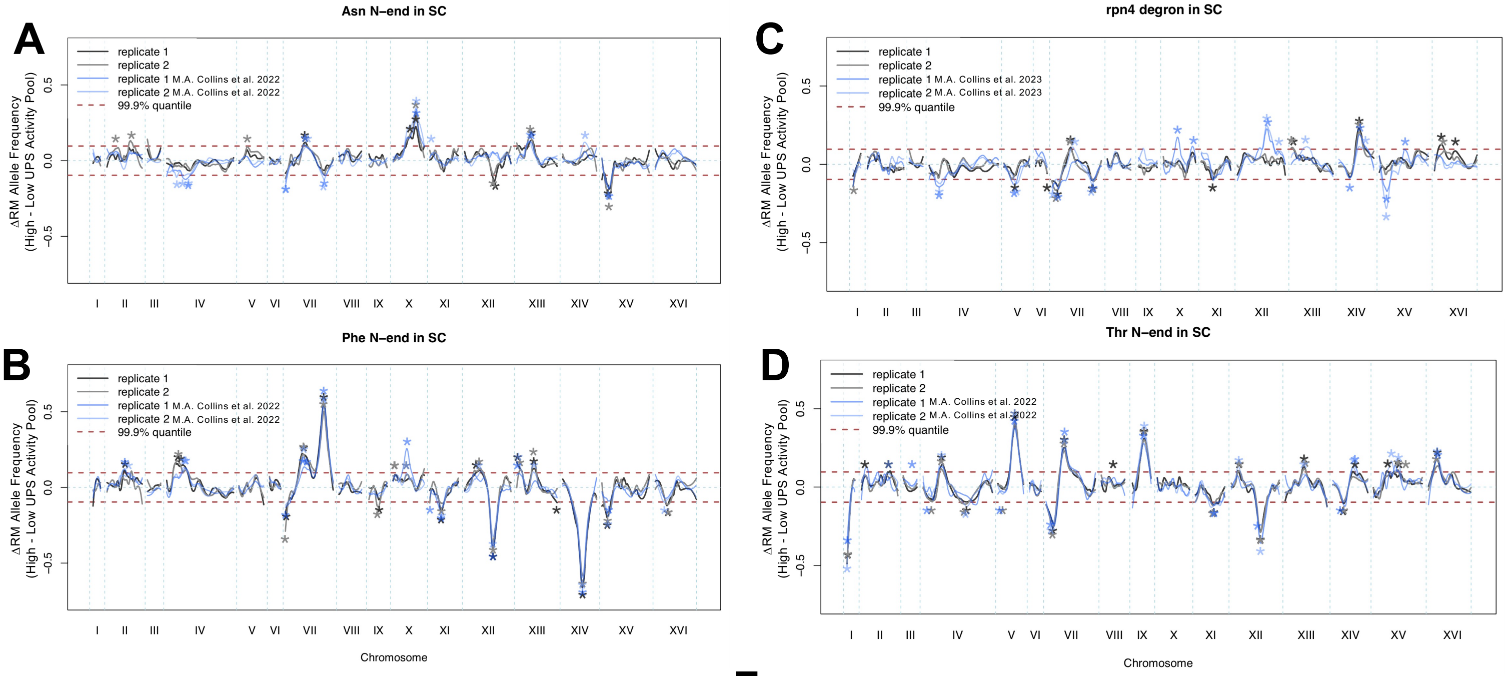

E

Reporter	Present in both studies	Total Comparisons	Percent Replicated
Asn	**4**	5	**80**
Phe	**11**	14	**79**
Rpn4	**3**	6	**50**
Thr	**12**	14	**86**

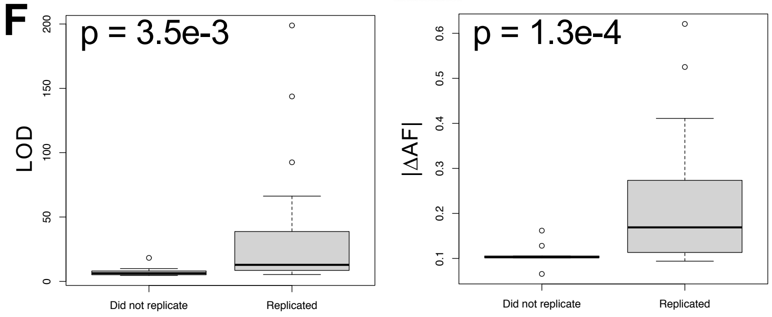

Supplementary Fig. 2: QTL reproducibility. A-D. QTL traces for the four reporters measured in SC in this study and M. A. Collins et al., 2022, 2023. **E.** Table summarizing the number of QTLs that replicated between studies. **F.** QTLs that did not replicate between studies had significantly lower LOD scores absolute delta allele frequencies.

bioRxiv preprint doi: https://doi.org/10.1101/2024.11.21.624644; this version posted November 21, 2024. The copyright holder for this preprint (which was not certified by peer review) is the author/funder, who has granted bioRxiv a license to display the preprint in perpetuity. It is made available under aCC-BY 4.0 International license.

A

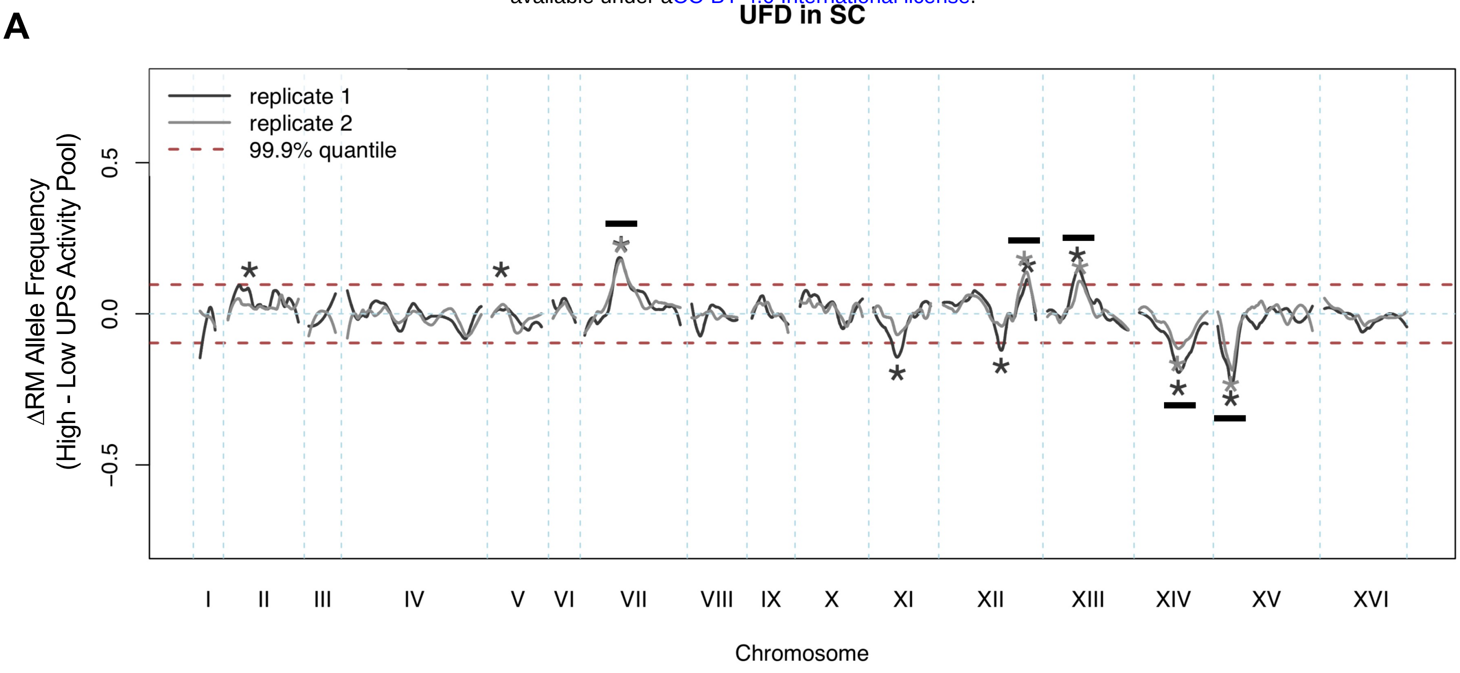

B

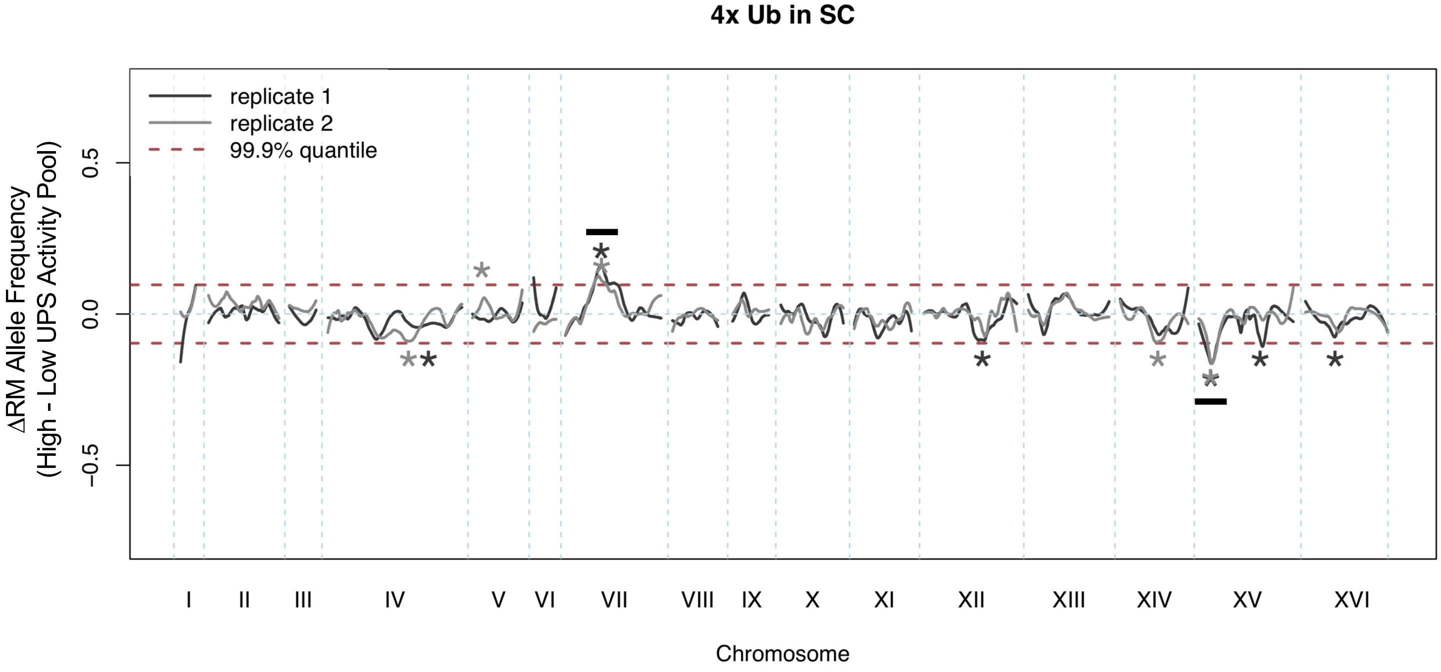

Supplementary Fig. 3: QTLs for 4xUb and UFD in SC. The plots show the loess-smoothed allele frequency difference between the high and low UPS activity pools across the genome for each of two independent biological replicates. Asterisks denote QTLs, defined by allele frequency differences that exceed an empirically-derived LOD score significance threshold in the given replicate. Horizontal black lines indicate QTLs that were present in both replicates. The dashed red horizontal lines denote an empirically-derived 99.9% quantile of the allele frequency difference. **A.** UFD reporter in SC. Five QTLs were present in both replicates. **B.** 4xUb reporter in SC. Two QTLs were present in both replicates.

bioRxiv preprint doi: https://doi.org/10.1101/2024.11.21.624644; this version posted November 21, 2024. The copyright holder for this preprint (which was not certified by peer review) is the author/funder, who has granted bioRxiv a license to display the preprint in perpetuity. It is made available under aCC-BY 4.0 International license.

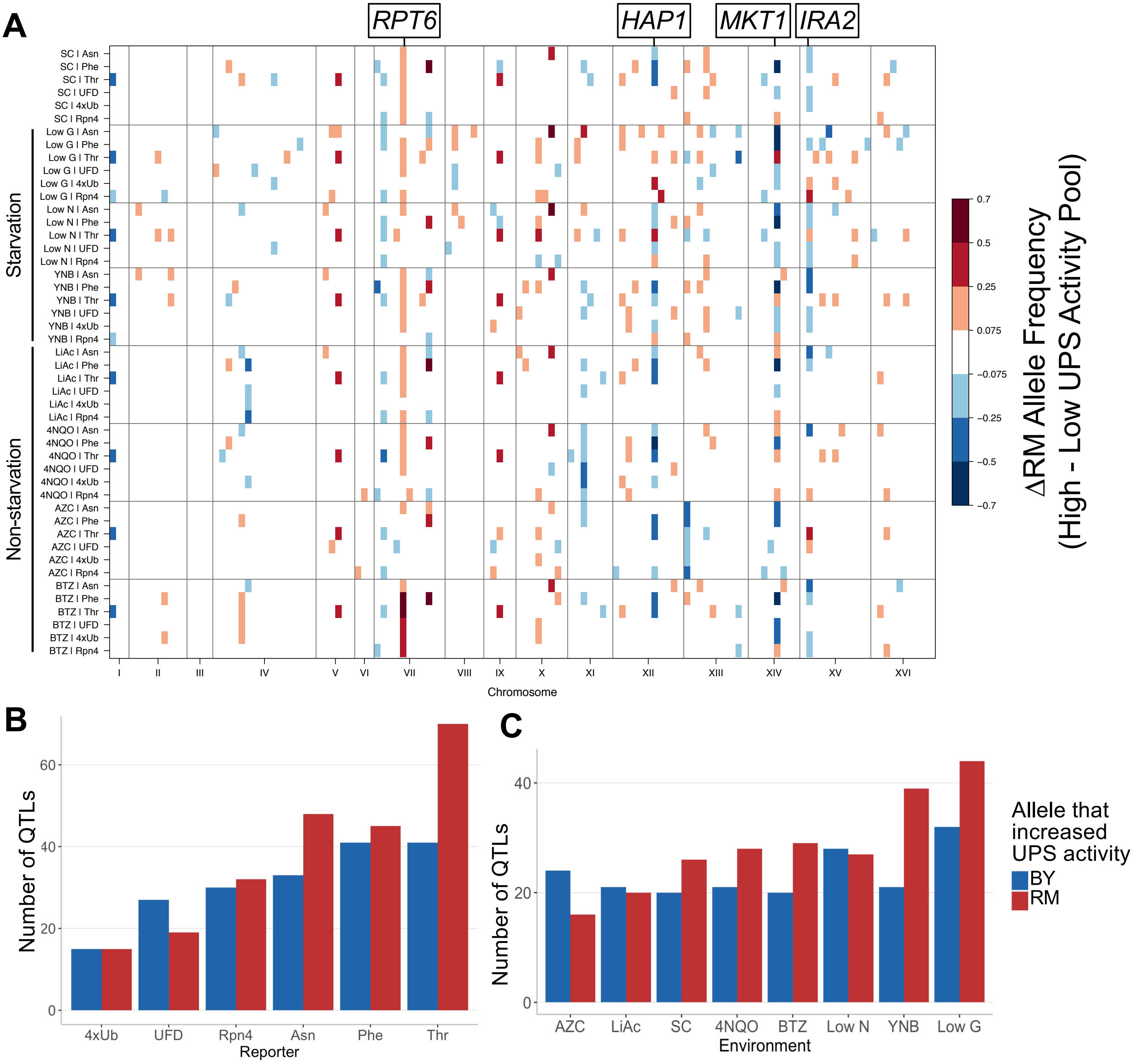

Supplementary Fig. 4: UPS activity QTLs across environments and reporters. A. QTLs for each reporter / environment combination. Data as in Fig. 3A, but reorganized according to environment. Colored blocks denote genome bins that contain QTLs detected in each of two independent biological replicates, colored according to the direction and magnitude of the effect size, expressed as the RM allele frequency difference between high and low UPS activity pools. Candidate causal genes discussed in the text are indicated. No data was collected for 4xUb in low nitrogen. **B.** Barplot showing the number of times the BY or RM allele increased degradation in the 416 QTLs, by reporter. **C.** Data as in B, but reorganized by environment.

bioRxiv preprint doi: https://doi.org/10.1101/2024.11.21.624644; this version posted November 21, 2024. The copyright holder for this preprint (which was not certified by peer review) is the author/funder, who has granted bioRxiv a license to display the preprint in perpetuity. It is made available under aCC-BY 4.0 International license.

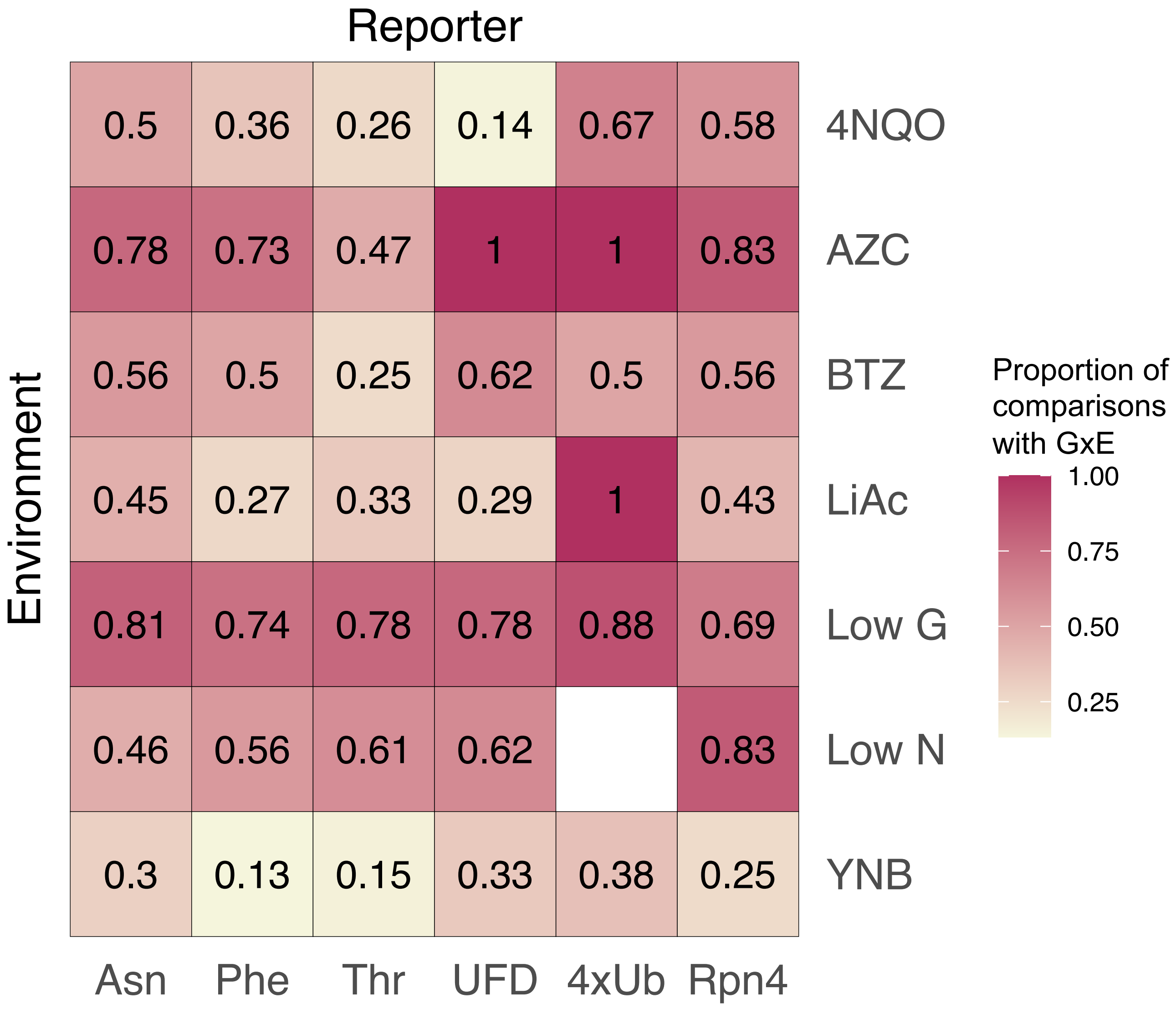

Supplementary Fig. 5: A heatmap showing the proportion of locus comparisons that showed GxE out of all comparisons, for combinations of reporters and environments. No data was collected for 4xUb in low nitrogen.

bioRxiv preprint doi: https://doi.org/10.1101/2024.11.21.624644; this version posted November 21, 2024. The copyright holder for this preprint (which was not certified by peer review) is the author/funder, who has granted bioRxiv a license to display the preprint in perpetuity. It is made available under aCC-BY 4.0 International license.

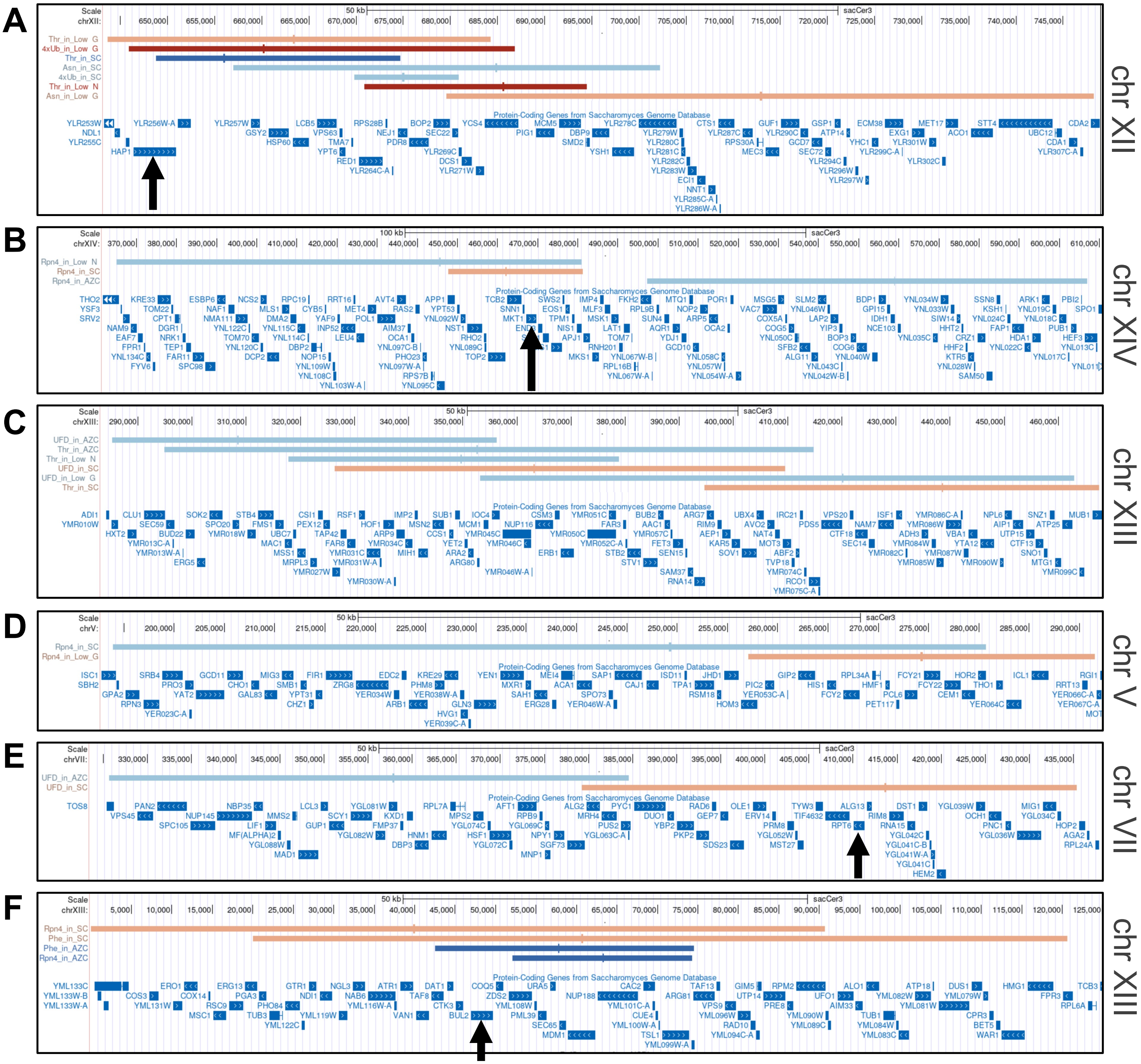

Supplementary Fig. 6: Locations of QTLs within sign change pairs: Locus plots of the 17 cases of sign change GxE (along with Fig. 5C). The confidence interval (horizontal bars) and peak position (short vertical lines) of the 29 unique QTLs involved in sign changes are shown along the *S. cerevisiae* genome (sacCer3) using UCSC Genome Browser (Nassar et al., 2023). For the QTLs, colors indicate direction and strength of effect as in Fig. 3A. Candidate causal genes are denoted with black arrows.
